# Novel COX-2 products of *n*-3 polyunsaturated fatty acid-ethanolamine-conjugates identified in RAW264.7 macrophages[S]

**DOI:** 10.1194/jlr.M094235

**Published:** 2019-08-27

**Authors:** Ian de Bus, Han Zuilhof, Renger Witkamp, Michiel Balvers, Bauke Albada

**Affiliations:** Laboratory of Organic Chemistry* Wageningen University and Research, Wageningen, The Netherlands; Nutritional Biology and Health Group, Division of Human Nutrition,† Wageningen University and Research, Wageningen, The Netherlands; School of Pharmaceutical Sciences and Technology,§ Tianjin University, Tianjin, People’s Republic of China and Department of Chemical and Materials Engineering, King Abdulaziz University, Jeddah, Saudi Arabia

**Keywords:** cyclooxygenase, fatty acid amides, fatty acid oxidation, high-performance liquid chromatography, inflammation, mass spectrometry, prostaglandins, cyclooxygenase 2

## Abstract

Cyclooxygenase 2 (COX-2) plays a key role in the regulation of inflammation by catalyzing the oxygenation of PUFAs to prostaglandins (PGs) and hydroperoxides. Next to this, COX-2 can metabolize neutral lipids, including endocannabinoid-like esters and amides. We developed an LC-HRMS-based human recombinant (h)COX-2 screening assay to examine its ability to also convert *n*-3 PUFA-derived *N*-acylethanolamines. Our assay yields known hCOX-2-derived products from established PUFAs and anandamide. Subsequently, we proved that eicosapentaenoylethanolamide (EPEA), the *N*-acylethanolamine derivative of EPA, is converted into PGE_3_-ethanolamide (PGE_3_-EA), and into 11-, 14-, and 18-hydroxyeicosapentaenoyl-EA (11-, 14-, and 18-HEPE-EA, respectively). Interestingly, we demonstrated that docosahexaenoylethanolamide (DHEA) is converted by hCOX-2 into the previously unknown metabolites, 13- and 16-hydroxy-DHEA (13- and 16-HDHEA, respectively). These products were also produced by lipopolysaccharide-stimulated RAW267.4 macrophages incubated with DHEA. No oxygenated DHEA metabolites were detected when the selective COX-2 inhibitor, celecoxib, was added to the cells, further underlining the role of COX-2 in the formation of the novel hydroxylated products. This work demonstrates for the first time that DHEA and EPEA are converted by COX-2 into previously unknown hydroxylated metabolites and invites future studies toward the biological effects of these metabolites.

Cyclooxygenase 2 (COX-2) is a nonconstitutional enzyme that is upregulated upon inflammation in order to generate inflammatory regulators (1–3). It is known to convert arachidonic acid (AA) into prostaglandin (PG)H_2_, the precursor of various inflammation-regulating PGs and thromboxanes (1, 2, 4). Although AA is considered the prototypical COX-2 substrate, the enzyme has a broad substrate specificity that includes other PUFAs and their derivatives, such as the endocannabinoid, arachidonoylethanolamide (AEA; also known as anandamide) (2, 5–11). Previous studies revealed that COX-2 converts AEA into PG ethanolamides (EAs), also called prostamides, and potent anti-inflammatory monohydroxylated AEAs (5, 6, 11–14). Although various amide- and ester-bound derivatives of AA are converted by COX-2 (5, 6, 13–17), the interaction between COX-2 and endocannabinoid-like molecules derived from PUFAs other than AA is barely investigated.

Previous work revealed that docosahexaenoylethanolamide (DHEA), an *n*-3 PUFA endocannabinoid-like metabolite, has potent anti-inflammatory properties in vitro (18). DHEA is present in human blood and a variety of animal tissues in a concentration that depends on the dietary intake of *n*-3 PUFAs as present in fish oil (19–22). Several studies showed interesting biological properties of DHEA. For example, DHEA is able to inhibit head and neck squamous cell carcinoma proliferation, the formation of pro-inflammatory cytokines, such as interleukin (IL)-6, and to induce the production of the anti-inflammatory cytokine, IL-10, which all results in strong anti-inflammatory effects (23–26). In addition, DHEA stimulates neurite growth, hippocampal development, and synaptogenesis in developing neurons in the central nervous system (27–31), hence the alternative name synaptamide for DHEA (29, 32). Recently, it was suggested that the neural and hippocampal stimulating roles are at least partly caused by the interaction of DHEA with the G protein-coupled receptor 110, stimulating a cAMP-dependent signal transduction pathway (28, 30). Previously, our group has demonstrated that DHEA reduces the production of the pro-inflammatory mediator PGE_2_ in lipopolysaccharide (LPS)-stimulated RAW264.7 macrophages, which could not be fully explained by the modest reduction of COX-2 protein expression (18). This observation suggests that DHEA may act as a COX-2 substrate, which reduces PG formation through competitive inhibition.

Several studies aimed at unraveling the metabolic fate of DHEA. Although the interactions of DHEA and 15-lipooxygenase (15-LOX) or cytochrome P450 (CYP450) were reported (22–24, 28), the possible interaction between COX-2 and DHEA is poorly understood. For instance, Serhan and coworkers reported that 15-LOX yields di-hydroxylated and epoxidated DHEA products. Two of these, i.e., 10,17-diHDHEA and 15-HEDPEA, were found to reduce platelet leukocyte aggregation, and 15-HEDPEA was even shown to be protective in vivo in murine hind limb ischemia and second organ reperfusion injury (23). Similarly, several CYP450-derived epoxide products of DHEA increased the formation of anti-inflammatory cytokines and exerted anti-angiogenic effects in human microvascular endothelial cells, vasodilatory actions on bovine coronary arteries, and reciprocally regulated platelet aggregation in washed human platelets (24). In view of the prominent role of COX-2 in inflammation regulation, we studied the interaction between DHEA and COX-2.

To this end, *n*-3 PUFA-ethanolamine-conjugates were applied in a cell-free enzymatic human recombinant (h)COX-2 assay, and a LC separation coupled to a high-resolution (HR) MS (LC-HRMS) method was used to identify yet unidentified COX-2 metabolites of eicosapentaenoylethanolamide (EPEA), i.e., PGE_3_-EA and 11-HEPE-EA (and the postulated products 14- and 18-HEPE-EA), and DHEA, i.e., 13- and 16-HDHEA. Then, we developed an ultrahigh-performance (UP)LC-MS/MS-based method to quantify the products and studied the role of COX-2 metabolism of DHEA in cells by measuring the COX-2-related production of 13- and 16-HDHEA in LPS-stimulated RAW264.7 macrophages. We show that formation of these products is inhibited upon addition of the selective COX-2 inhibitor, celecoxib, proving a COX-2-dependent metabolism.

## MATERIALS AND METHODS

### Chemicals and materials

COX-2 (hCOX-2), AA (≥98% purity), EPA (≥98% purity), DHA (≥98% purity), EPEA (≥98% purity), DHEA (≥98% purity), DHEA-*d*_4_ [≥99% purity deuterated forms (*d*_1_–*d*_4_)], PGE_2_-EA-*d*_4_ [≥99% purity deuterated forms (*d*_1_–*d*_4_)], PGE_3_ (≥98% purity), (±)11-HEPE (≥98% purity), (±)13-hydroxydocosahexaenoic acid (HDHA) (≥98% purity), (±)16-HDHA (≥98% purity), and celecoxib (≥98% purity) were purchased at Cayman Chemicals (supplied by Sanbio B.V., Uden, The Netherlands). The lactate dehydrogenase (LDH) cytotoxicity kit was purchased at Roche Applied Science (Almere, The Netherlands). Anandamide (100% purity from HPLC) was obtained from Tocris Chemicals (Abingdon, UK). DMEM was purchased from Corning (supplied by VWR Chemicals, Amsterdam, The Netherlands); FBS, streptomycin, and penicillin were obtained from Lonza (Verviers, Belgium). Hematin porcine, Triton™ X-100, LPS, butylated hydroxytoluene (BHT) (99%), isobutyl chloroformate (98%), triethylamine (99.5%), ethanolamine (≥98%), ethanol-1,1,2,2-*d*_4_-amine (98 atom % D), and ethanol (EtOH; absolute for analysis) were purchased at Sigma-Aldrich (Zwijndrecht, The Netherlands). Ethyl acetate and *n*-heptane were acquired from VWR Chemicals. Trizma base (99%), phenol (>99%, for biochemistry), DMSO (>99.7%), and methanol (MeOH) (99.99%, LC/MS grade) were obtained from Fisher Scientific (Landsmeer, The Netherlands). Formic acid (99%, ULC/MS-CC/SFC) and acetonitrile (ACN) (ULC/MS-CC/SFC) were purchased at Biosolve Chemicals (Valkenswaard, The Netherlands). The HLB solid phase extraction columns (Oasis; 60 mg, 3cc) were obtained from Waters (Etten-Leur, The Netherlands). Ultrapure water was filtered by a MilliQ Integral 3 system from Millipore (Molsheim, France).

### Enzymatic cell-free COX-2 assay

A cell-free enzymatic hCOX-2 assay was performed similar to previously reported protocols (33–35). A 0.1 M Tris/HCl buffer (pH 8.0) was prepared containing 50 μM of Na_2_EDTA to stabilize the enzyme in solution. For the enzymatic incubations, 179 μl of buffer were mixed with 2 μl of 100 μM hematin solution in DMSO, 10 μl of 25 mM phenol solution in buffer and 5 μl of hCOX-2 in buffer giving a total amount of 1.0 μg of enzyme per reaction. In the negative control, 5 μl of buffer were added instead of 5 μl of hCOX-2 solution. The mixtures were preincubated for 2 min at room temperature before adding 4 μl of a 1 mM substrate solution in EtOH, giving a final substrate concentration of 20 μM. The reaction mixture was heated at 37°C for 20 min before quenching the reaction with 5 μl of formic acid. Eight hundred microliters of ethyl acetate:heptane (1:1, v/v) were used to extract the substrates and metabolites, after which the organic solvent was evaporated under reduced pressure at 40°C. The dried extract was reconstituted with 200 μl of EtOH and analyzed on the LC-HRMS system (see below). For the UPLC-MS/MS-based quantification of the EPEA and DHEA metabolites, the reactions were quenched with 5 μl of formic acid followed by the addition of 2 ml of MeOH containing 806 pg/mL product (11-HEPE-EA-*d*_4_ or PGE_2_-EA-*d*_4_ or 13-HDHEA-*d*_4_) and 1,000 pg/mL starting material (DHEA-*d*_4_) as internal standards. Thereafter, the metabolites were collected by SPE using HLB columns (see below).

### LC-HRMS analysis

LC-HRMS analyses were performed using a Finnigan Surveyor Plus HPLC from Thermo Fischer Scientific (Breda, The Netherlands) coupled to a Q-Exactive Focus Quadrupole Orbitrap high-resolution mass spectrometer equipped with a higher-energy collisional dissociation chamber, also from Thermo Fischer Scientific. The machine was operated at a mass resolution of 70,000 to allow the chemical characterization of the products. Ionization was performed using a heated electrospray ionization source applying a spray voltage of 3.2 kV and a capillary temperature of 300°C using polarity switching (1 s for a cycle of one positive and one negative scan). For the fragmentation analysis, the collision energies were optimized per ion. Chromatography was performed on a Reprosil Gold 120 C8, 3 μm column of 250 × 3 mm (Dr. Maisch GmbH, Germany), using the following eluents and gradient. Eluent A consisted of water/ACN (95/5) with 0.1% formic acid; eluent B consisted of 100% ACN with 0.1% formic acid. For elution of the various lipids and oxylipins, the program started isocratically with 50% B in A. From 3 to 8 min, the concentration was linearly increased to 100% B, and the run was continued with 100% B until 15 min. Then the gradient was decreased to 50% B in A in 2 min, and run with 50% B in A until 21 min. During the run, column temperature was kept at 30°C and the sample tray was cooled to 4°C to limit auto-oxidation. For the runs, 25 μl or 10 μl of sample were injected.

Data analysis of the mass spectra and the extracted ion chromatograms were performed using Thermo Xcalibur software version 3.0 from Thermo Fischer Scientific. All extracted ion chromatograms displayed are within a range of 0.03 Da. All chromatograms were processed with peak smoothening using boxcar smoothening to give representative peaks, unless stated otherwise. The displayed fragmentation spectra were obtained after background subtraction and were reproducible in multiple experiments.

### In vitro RAW264.7 macrophage incubations

To verify whether DHEA is also metabolized in COX-2-expressing cells, RAW264.7 macrophages (American Type Culture Collection, Teddington, UK) were cultured in DMEM containing 10% FCS and 1% penicillin and streptomycin at 37°C in a 5% CO_2_ humidified incubator. For the in vitro experiments, 2 ml of 250,000 cells/ml were seeded and incubated for 24 h in 6-well plates. The medium of the adherent cells was discarded and replaced by new fresh medium, and the various compounds were subsequently added for the experiments. To investigate COX-2-mediated conversion of DHEA, RAW macrophages were preincubated for 30 min with 10 μM of DHEA, followed by stimulation with 1.0 μg/ml LPS. In the celecoxib control experiments, the RAW macrophages were preincubated with 0.3 μM of celecoxib before DHEA incubation. After 24 h, the lipids were extracted for metabolite identification (see below).

### Cell viability and cytotoxicity determination

To assess the viability of the macrophages after the various treatments, microscopic evaluation of the wells was performed after incubation. Pictures were taken with a LEICA DFS450C microscope from Leica Microsystems (Amsterdam, The Netherlands), and white balancing was applied to optimize the color brightness of each picture.

Cell cytotoxicity was tested in the medium based on the LDH concentration. The LDH assay was performed according to the manufacturer’s instructions.

### PGE_2_ quantification

Quantification of PGE_2_ was done by performing PGE_2_ EIA assays. A PGE_2_ ELISA kit (monoclonal) was purchased at Cayman Chemical (supplied by Sanbio B.V.) and the ELISA was performed according to the supplied instructions.

### Intracellular lipid extraction from LPS-stimulated RAW264.7 cells

Extraction of intracellular lipids from incubated RAW264.7 macrophages was performed using an adapted version of a previously described oxylipin extraction protocol, which was optimized for adherent cells. (19). In short, the medium was replaced by 1 ml of fresh medium and the macrophages were scraped from the culture plates in order to suspend them in the fresh medium. The cells were centrifuged (330 *g* at 4°C) for 5 min and the supernatant was discarded. Subsequently, the macrophages were treated with 1 ml of MeOH containing 806 pg/ml 13-HDHEA-*d*_4_ as an internal standard. This suspension was sonicated for 5 min and centrifuged (330 *g* at 4°C) for 5 min, after which the supernatant was stored in a clean 15 ml falcon tube. The pellet was treated a second time with 1 ml of fresh MeOH containing 806 pg/ml 13-HDHEA-*d*_4_. After 5 min sonication and 5 min centrifugation (330 *g* at 4°C), this supernatant was also transferred to the 15 ml falcon tube containing the supernatant of the first extraction. The combined extracts were diluted with 8 ml of ultrapure water containing 0.125% formic acid before solid-phase extraction of the metabolites on HLB columns. The columns were preactivated using 2 ml of MeOH and 2 ml of ultrapure water containing 0.1% formic acid, respectively. Then the columns were loaded with the extract and rinsed using 2 ml of 20% MeOH in ultrapure water containing 0.1% formic acid. The lipids were eluted using 1 ml of MeOH containing 0.1% formic acid and collected in tubes filled with 20 μl of 10% glycerol and 500 μM of BHT in EtOH (BHT was present to prevent auto-oxidation of the products). The samples were evaporated to dryness using a speedyvac concentrator (Salm and Kipp, Breukelen, The Netherlands) and were redissolved in 100 μl of EtOH. The samples were directly analyzed by UPLC-MS/MS or stored at −80°C and analyzed at a later time point.

### UPLC-MS/MS analysis

For improved chromatographic separation and sensitivity, an UPLC-MS/MS method was developed to quantify the hydroxylated fatty acid-ethanolamine-conjugates. The standards used for the quantification were either commercially available or synthesized as described in the supplemental information. The analyses were performed on an I-class fixed-loop UPLC coupled to a Xevo TQ-S triple-quadrupole mass spectrometer (Waters). The electron spray interface was operated with a capillary voltage of 4.50 kV, a cone voltage of 50 V, and a desolvation temperature of 600°C. The mass spectrometer was operated in multiple reaction mode (MRM), with mass transitions and collision energies that were optimized per component (supplemental Table S1). Chromatographic separation was performed on a Zorbax Eclipse Plus C18 Rapid Resolution HD, 1.8 μ column of 2.1 × 150 mm (Agilent, Amstelveen, The Netherlands), using an elution profile identical to a method reported by Balvers et al. (19). In short, eluent A consisted of water/ACN (95/5) with 0.1% formic acid; eluent B consisted of 100% ACN with 0.1% formic acid. The program started with 5% B in A followed by a linear increase to 30% B in A, which was achieved at 5.0 min. This was followed by a linear increase toward 50% B in A, which was achieved at 11.25 min and maintained until 13.25 min. The system was subsequently switched to 100% B, which was achieved at 15.75 min and maintained until 16.75 min, after which the column was left to equilibrate at 5% B in A for approximately 3 min. During the run, the column temperature was set to 60°C and the sample tray was cooled to 4°C to limit auto-oxidation. For each run, 3 μl of sample was injected.

The results were analyzed using MassLynx 4.1 software (Waters). Quantification of the HDHEA levels was performed using linear regression of the response ratios (peak area HDHEA/peak area internal standard) from the calibration curve as a function of the corresponding HDHEA concentration. Depending on the experiment, the data were weighted 1/x or 1/x^2^ to obtain high accuracy of the back-calculated concentrations throughout the calibrated range. The accuracy and precision of the method were determined by quality control samples.

## RESULTS

### Validation of the hCOX-2 assay using AA, EPA, DHA, and AEA

The validity of our developed cell-free hCOX-2 assay was shown using the COX-2-dependent oxidation of the PUFAs, AA, EPA, DHA, and AEA. As expected, AA was converted into PGE_2_ and PGD_2_ (supplemental Fig. S1) (2, 35, 36). In addition, monohydroxylation on C11 or C15 was proven using mass fragmentation, resulting in 11- and 15-HETE (supplemental Fig. S2) (2, 35, 36); a nonspecific auto-oxidation product was also observed, which is a common feature for PUFAs (37). Similarly, EPA oxidized to PGE_3_, PGD_3_, 11-HEPE, and 14-HEPE, which is in line with COX-2 products reported in the literature (supplemental Figs. S3, S4) (35, 38). Lastly, our assay and mass fragmentation confirmed that DHA was converted into 13-HDHA (supplemental Fig. S5) (35, 39, 40).

When the neutral endocannabinoid derivative, AEA, was added to our assay, we found that it was converted into PGE_2_-EA and PGD_2_-EA. These structures are consistent with other reports (5, 11, 13–16), demonstrating that our in vitro hCOX-2 assay is capable of identifying these metabolites. Specifically, PGE_2_-EA and PGD_2_-EA were found as [M+H]^+^ with *m/z* = 396.2741 and with lesser intensity as [M+Na]^+^ with *m/z* = 418.2559. Fragmentation of the [M+H]^+^ ion of the prostamides using a collision energy of 20.0 electronvolts (eV) in the higher-energy collisional dissociation chamber showed identical fragmentation products as reported in the literature (supplemental Fig. S6) (13, 14, 36). In addition to the prostamides, we also observed two monohydroxylated HETE-EAs (14) as the sodium adduct [M+Na]^+^ at *m/z* = 386.266 (supplemental Fig. S7) and, in the negative mode, as the formate adduct [M+HCO_2_]^–^ at *m/z* = 408.2757. Mass fragmentation on both species provided evidence for the formation of 11- and 15-HETE-EA (supplemental Fig. S7, **Table 1**) (7).

**TABLE 1.** Overview of COX-2-derived monohydroxylated products of *N*-acylethanolamides derived from PUFAs and their observed ions in the mass spectrometer

Product	[M+H–H_2_O]^+^^+^	[M+Na]^+^^+^	[M+CHO_2_]^–^
11-HETE-EA	*m/z* = 346.27432	*m/z* = 386.26647	*m/z* = 408.27537
		243.14639 [M+Na–C_10_H_16_O]^+^^+^	362.27059 [M–H]^–^
		(γ-ene rearrangement)	344.25942 [M–H–H_2_O]^–^
		192.09961 [M+Na–C_13_H_22_O]^+^^+^	210.15000 [M–H–C_10_H_16_O]^+^^+^
			(γ-ene rearrangement)
15-HETE-EA	*m/z* = 346.27432	*m/z* = 386.26679	*m/z* = 408.27506
		286.17900 [M+Na–C_6_H_12_O]^+^^+^	344.25939 [M–H–H_2_O]^–^
		(β-ene rearrangement)	262.18108 [M–H–C_6_H_12_O]^–^
			(β-ene rearrangement)
11-HEPE-EA	*m/z* = 344.26808	*m/z* = 384.26235	*m/z* = 406.25990
		234.14837 [M+Na–C_10_H_14_O]^+^^+^	360.25456 [M–H]^–^
		(γ-ene rearrangement)	342.24351 [M–H–H_2_O]^–^
		192.09903 [M+Na–C_13_H_20_O]^+^^+^	238.14488 [M–H–C_9_H_14_]^–^
			(β-ene rearrangement)
			210.15000 [M–H–C_10_H_14_O]^–^
			(γ-ene rearrangement)
14-HEPE-EA	*m/z* = 344.26808	*m/z* = 384.26236	*m/z* = 406.25990
		274.18165 [M+Na–C_7_H_10_O]^+^^+^	360.25449 [M–H]^–^
		(γ-ene rearrangement)	342.24350 [M–H–H_2_O]^–^
		232.13275 [M+Na–C_10_H_16_O]^+^^+^	250.18128 [M–H–C_7_H_10_O]^–^
			(γ-ene rearrangement)
18-HEPE-EA	*m/z =* 344.26808	*m/z =* 384.26237	*m/z* = 406.25973
		No fragment ions observed	342.24318 [M–H–H_2_O]^–^
			255.21163 [C_19_H_27_]^–^
			215.17966 [C_16_H_23_]^–^
			(β-ene rearrangement)
13-HDHEA	*m/z* = 370.27433	*m/z* = 410.26642	*m/*z = 432.27555
		392.25680 [M+Na–H_2_O]^+^^+^	368.25959 [M–H–H_2_O]^–^
		260.16186 [M+Na–C_10_H_14_O]^+^^+^	264.16037 [M–H–C_9_H_14_]^–^
		(γ-ene rearrangement)	(β-ene rearrangement)
		218.11530 [M+Na–C_13_H_20_O]^+^^+^	236.16560 [M–H–C_10_H_14_O]^–^
			(γ-ene rearrangement)
16-HDHEA	*m/z* = 370.27433	*m/z* = 410.26657	*m/*z = 432.27553
		392.25485 [M+Na–H_2_O]^+^^+^	368.25904 [M–H–H_2_O]^–^
		300.19316 [M+Na–C_7_H_10_O]^+^^+^	276.19677 [M–H–C_7_H_10_O]^–^
		(γ-ene rearrangement)	(γ-ene rearrangement)
		258.14638 [M+Na–C_10_H_16_O]^+^^+^	

+ For the [M+Na]^+^ and [M+CHO_2_]^–^ adducts, the observed fragment ions and proposed rearrangements are given.

After validation of our hCOX-2 assay, we studied EPEA and DHEA as two potentially new substrates for COX-2.

### hCOX-2-derived metabolites of the *n*-3 fatty acid EAs, EPEA and DHEA

**Fig. 1.**
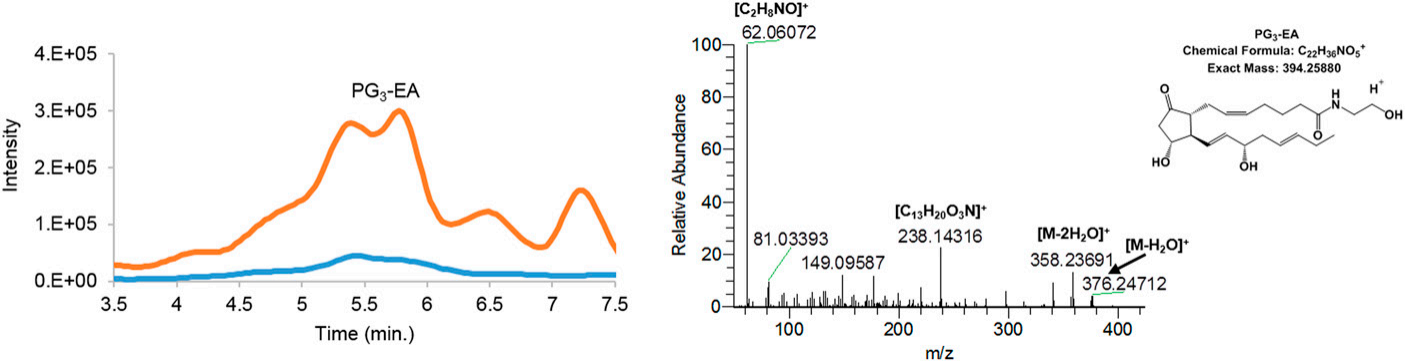
Extracted ion chromatogram of *m/z* = 394.24–394.27 of PG_3_-EA [M+H]^+^. The orange trace in the chromatogram depicts the products of EPEA formed by hCOX-2; the blue trace shows the control assay without hCOX-2. The mass fragmentation spectrum corresponds to the product eluting at 5.10–5.42 min using 20.0 eV collision energy.

**Fig. 2.**
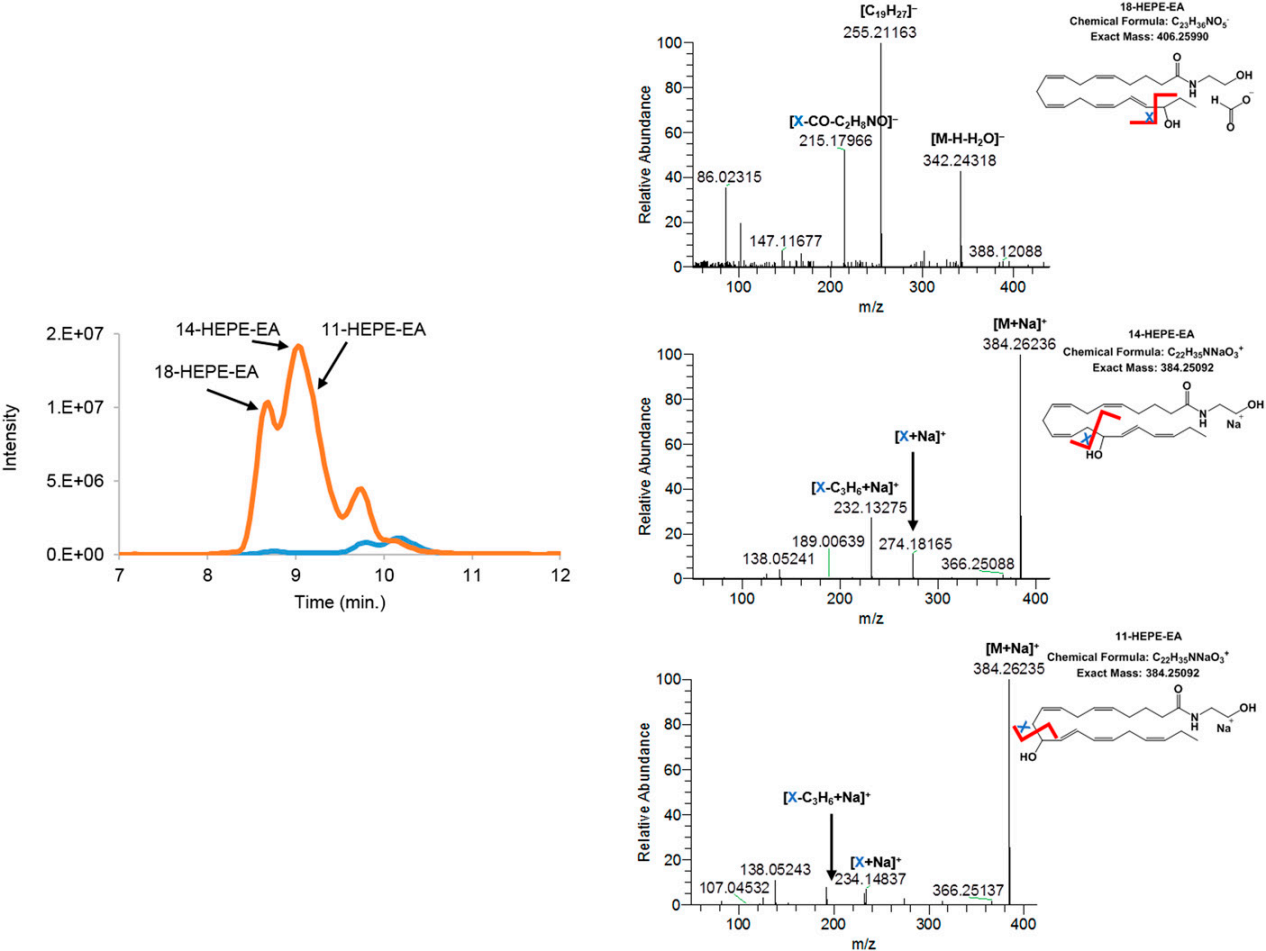
Extracted ion chromatogram of *m/z* = 344.25–344.27 of HEPE-EAs [M+H–H_2_O]^+^. The orange trace in the chromatogram depicts the products of EPEA formed by hCOX-2; the blue trace shows the control assay without hCOX-2. Mass fragmentation spectra of hCOX-2 products were obtained by fragmenting the [M+HCO_2_]^–^ adduct using 20.0 eV and/or the [M+Na]^+^ adduct using 30.0 eV. The fragmentation spectra of the postulated 18-HEPE-EA is averaged from 8.43 to 8.82 min, 14-HEPE-EA from 8.81 to 9.01 min, and 11-HEPE-EA from 9.04 to 9.41 min.

Incubation of hCOX-2 with EPEA led to the formation of PG_3_-EA and HEPE-EA products. Fragmentation of the *m/z* = 394.2589 [M+H]^+^ parent mass of the prostamide product of EPEA showed a fragmentation pattern corresponding with prostamide structures (**Fig. 1**). The elution profile suggested production of at least two different PG_3_-EA products, most likely PGE_3_-EA and PGD_3_-EA (see below). Evidence for the formation of three monohydroxylated products of EPEA was also found, i.e., 11-, 14-, and 18-HEPE-EA. As was observed for the AEA-derived products, the [M+H–H_2_O]^+^ ion with *m/z* = 344.26808 was most abundant in the positive ionization mode, together with [M+Na]^+^ with *m/z* = 384.26236. In negative ionization mode, [M+HCO_2_]^–^ was detected at *m/z* = 406.25990 (7, 36). Based on the fragmentation pattern of the formate adduct or the sodium adduct, we postulated formation of 11-, 14-, and 18-HEPE-EA (**Fig. 2**, Table 1). Of these products, 18-HEPE-EA eluted earlier than 14-HEPE-EA and 11-HEPE-EA, which is analogous to the elution of HEPE products described by Smith and coworkers (35). Fragmentation of the formate adduct resulted in a mass fragment with *m/z =* 215.17966 (chemical composition of C_16_H_23_). This product is most likely the result of a β-ene rearrangement leading to formation of one of the main fragments of 18-HEPE (35, 41). The sodium adduct of 14-HEPE-EA fragmented between C13 and C14, resulting in a mass ion of *m/z* = 274.18165 (chemical composition of C_15_H_25_NNaO_2_) that most likely resulted from a γ-ene rearrangement. Fragmentation of 11-HEPE-EA resulted in a fragment ion of *m/z* = 234.14837 (chemical composition of C_12_H_21_NNaO_2_), which resulted from a γ-ene rearranged cleavage between C10 and C11 (41). The 11- and 14-HEPE-EA fragments were found to undergo a sequential neutral loss of C_3_H_6_ (Fig. 2), as was also observed for 11-HETE-EA (supplemental Fig. S7). Fragmentation analysis on the formate adduct of the two latter products again showed bond fission between C10–C11 and C13–C14, supporting the formation of 11- and 14-HEPE-EA (Table 1). Further evidence was obtained using a chemically synthesized 11-HEPE-EA standard that showed identical retention time and mass fragmentation pattern as the postulated 11-HEPE-EA product (supplemental Fig. S8).

**Fig. 3.**
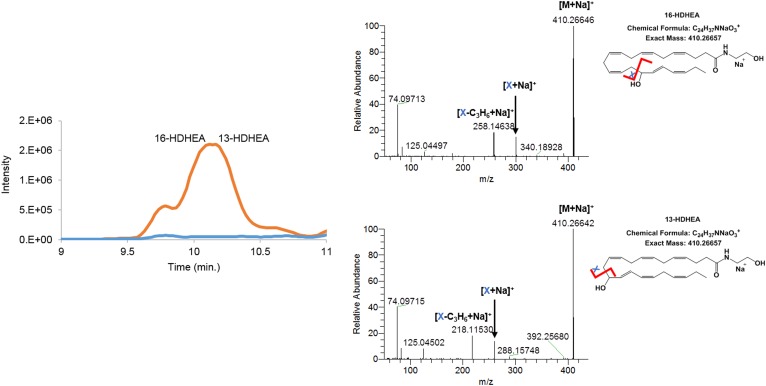
Extracted ion chromatogram of *m/z* = 370.26–370.28 HDHEAs [M+H–H_2_O]^+^. The orange trace in the chromatogram depicts the products of DHEA formed by hCOX-2; the blue trace shows the control assay without hCOX-2. Mass fragmentation spectra of both hCOX-2 products were obtained by fragmenting the [M+Na]^+^ adduct from 9.91 to 10.04 min for 16-HDHEA and from 10.19 to 10.24 min for 13-HDHEA using 30.0 V collision energy.

For DHEA, only monohydroxylated products and no cyclooxygenated products were identified. Again, an in-source fragment with the loss of water of *m/z* = 370.27433 and a sodium adduct of *m/z* = 410.26642 for 13-HDHEA and *m/z* = 410.26657 for 16-HDHEA were observed for the hydroxylated product in the positive ionization mode. The negative ionization mode showed a formate adduct of *m/z* = 432.2755. Based on the fragmentation spectra of the [M+Na]^+^ ion, using a collision energy of 30.0 eV, it was concluded that 13- and 16-HDHEA are the main enzymatic products of DHEA (**Fig. 3**). Both products were not chromatographically resolved. Mass fragmentation resulted in a fragment ion of *m/z* = 260.16186 corresponding to a chemical structure of C_14_H_23_NNaO_2_^+^. This fragment matched with the proposed γ-ene rearranged product of 13-HDHEA. A second fragment of *m/z* = 300.19316 corresponded to a chemical structure of C_17_H_27_NNaO_2_^+^, which matched with the γ-ene rearranged product of 16-HDHEA. Again, the HDHEA fragments were prone to a neutral loss of C_3_H_6_ (Fig. 3). Also, in the negative ionization mode, the fragmentation performed on the formate adduct gave rise to the expected loss of a water fragment (*m/z* = 368.25959), a γ-ene-rearranged fragment (*m/z* = 236.16560), and a β-ene rearranged fragment (*m/z* = 264.16037) for 13-HDHEA. Similarly, the expected loss of a water fragment (*m/z* = 368.25904) and a γ-ene-rearranged fragment (*m/z* = 276.19677) were observed for 16-HDHEA (Table 1). To confirm our observations, we synthesized 13- and 16-HDHEA as analytical standards and observed that these standards eluted, ionized, and fragmented identically (supplemental Figs. S9, S10).

### UPLC-MS/MS analysis of hCOX-2 enzymatic incubation of DHEA and EPEA

**Fig. 4.**
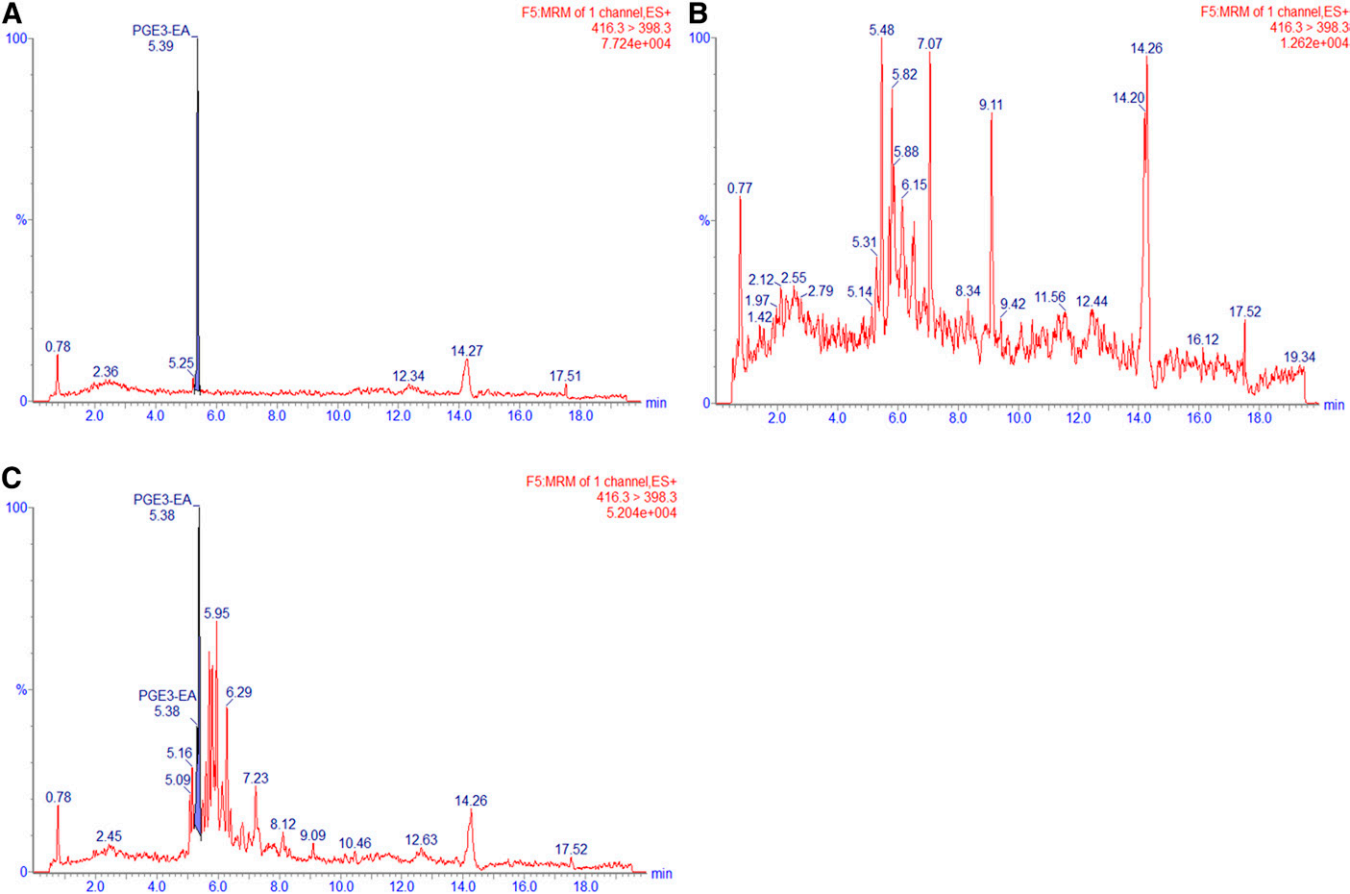
MRM chromatograms of *m/z* = 416 > 398 for the 10 ng spiked PGE_3_-EA standard (A), control incubation of EPEA without hCOX-2 (B), and hCOX-2 incubation of EPEA (C).

**Fig. 5.**
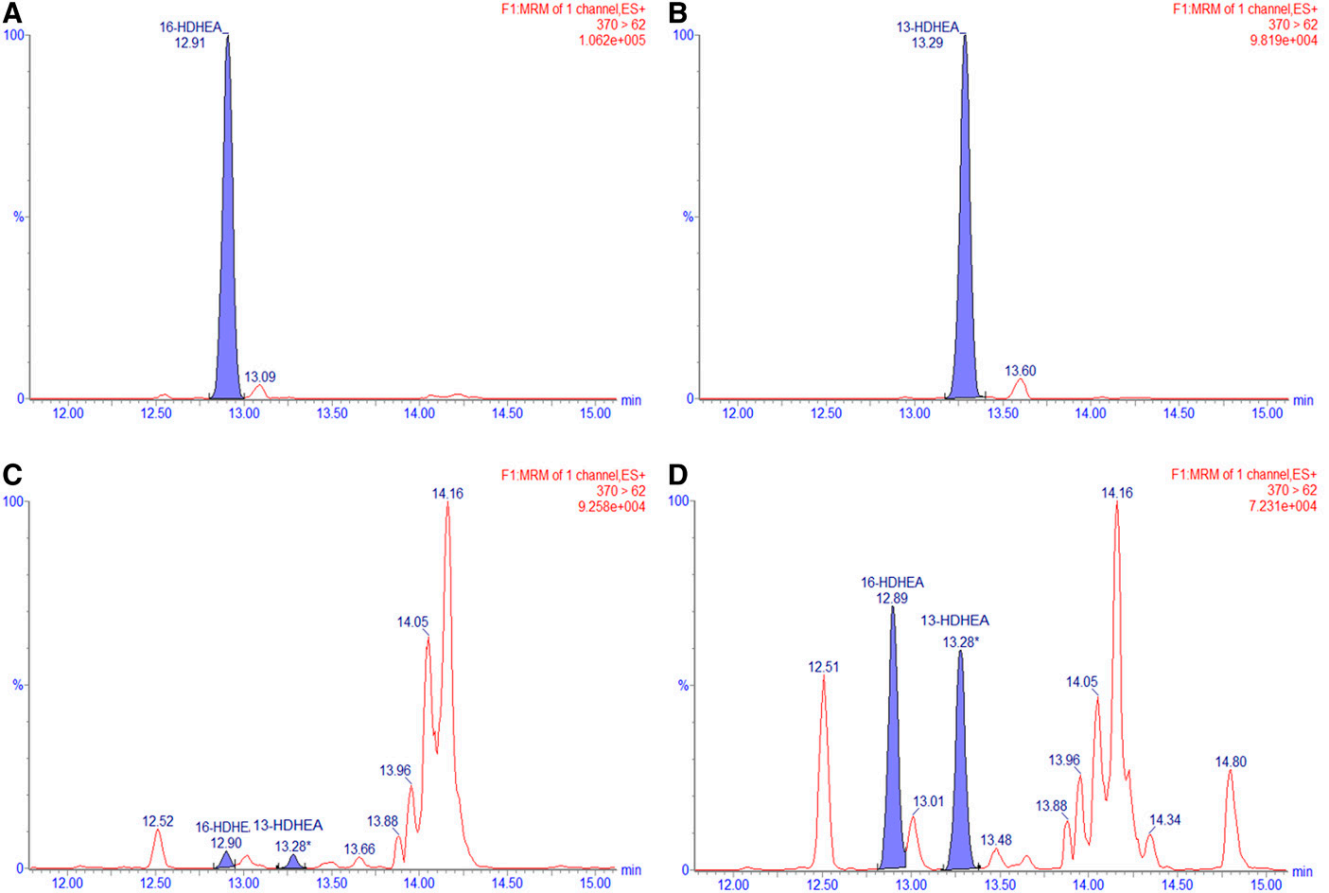
MRM chromatograms of *m/z* = 370 > 62 for the synthetic standards of 16-HDHEA (A), 13-HDHEA (B), control incubation of DHEA without hCOX-2 (C), and hCOX-2 incubation of DHEA (D).

For further investigations into the metabolism of EPEA and DHEA and to quantify the hCOX-2-derived products of EPEA and DHEA, we developed an analytical method with sufficient chromatographic separation and increased analytical sensitivity. For this, we synthesized appropriate analytical standards by coupling ethanolamine or ethanolamine-*d*_4_ to commercially available monohydroxylated fatty acid precursors (supplemental Figs. S16–S63 and accompanying material). Thereafter, a targeted UPLC-MS/MS method was developed for the quantification of PGE_3_-EA, 11- and HEPE-EAs, and 13- and 16-HDHEA (supplemental Table S1). We successfully quantified PGE_3_-EA formation at 28.5 (± 1.1) pmol (∼0.7% conversion) (**Fig. 4**, **Table 2**). Although exact quantification of HEPE-EAs was not successful due to substantial interfering auto-oxidation of EPEA, we estimate that HEPE-EAs were formed in the same order of magnitude as the PGE_3_-EA product, i.e., with ∼1% conversion because the peak areas were of the same order of magnitude. The 13- and 16-HDHEA were baseline separated by our UPLC-MS/MS method, and we were able to quantify both mono-oxygenated products: 55.9 (± 9.1) pmol (∼1.4% of DHEA) for 13-HDHEA and 52.1 (± 6.5) pmol (∼1.3% of DHEA) for 16-HDHEA (**Fig. 5**, Table 2).

**TABLE 2.** Identified products and yields of the enzymatic conversion of EPEA to PGE_3_-EA and DHEA to 13- and 16-HDHEA by hCOX-2 as quantified by UPLC-MS/MS

Substrate*^a^*	hCOX-2 Product	Amount of Product Formed (pmol)	Yield (%)
EPEA (4 nmol)	PGE_3_-EA	28.5 (± 1.1)	0.7 (± 0.02)
DHEA (4 nmol)	13-HDHEA	55.9 (± 9.1)	1.4 (± 0.05)
DHEA (4 nmol)	16-HDHEA	52.1 (± 6.5)	1.3 (± 0.03)

Data are presented as the mean from three independent experiments containing biological duplicates with standard error of the mean between parentheses.

a Amounts in parentheses in the first column are the nanomoles of substrate added in the assay.

### 13-HDHEA and 16-HDHEA are formed from DHEA in LPS-stimulated RAW264.7 macrophages by a COX-2-dependent process

**Fig. 6.**
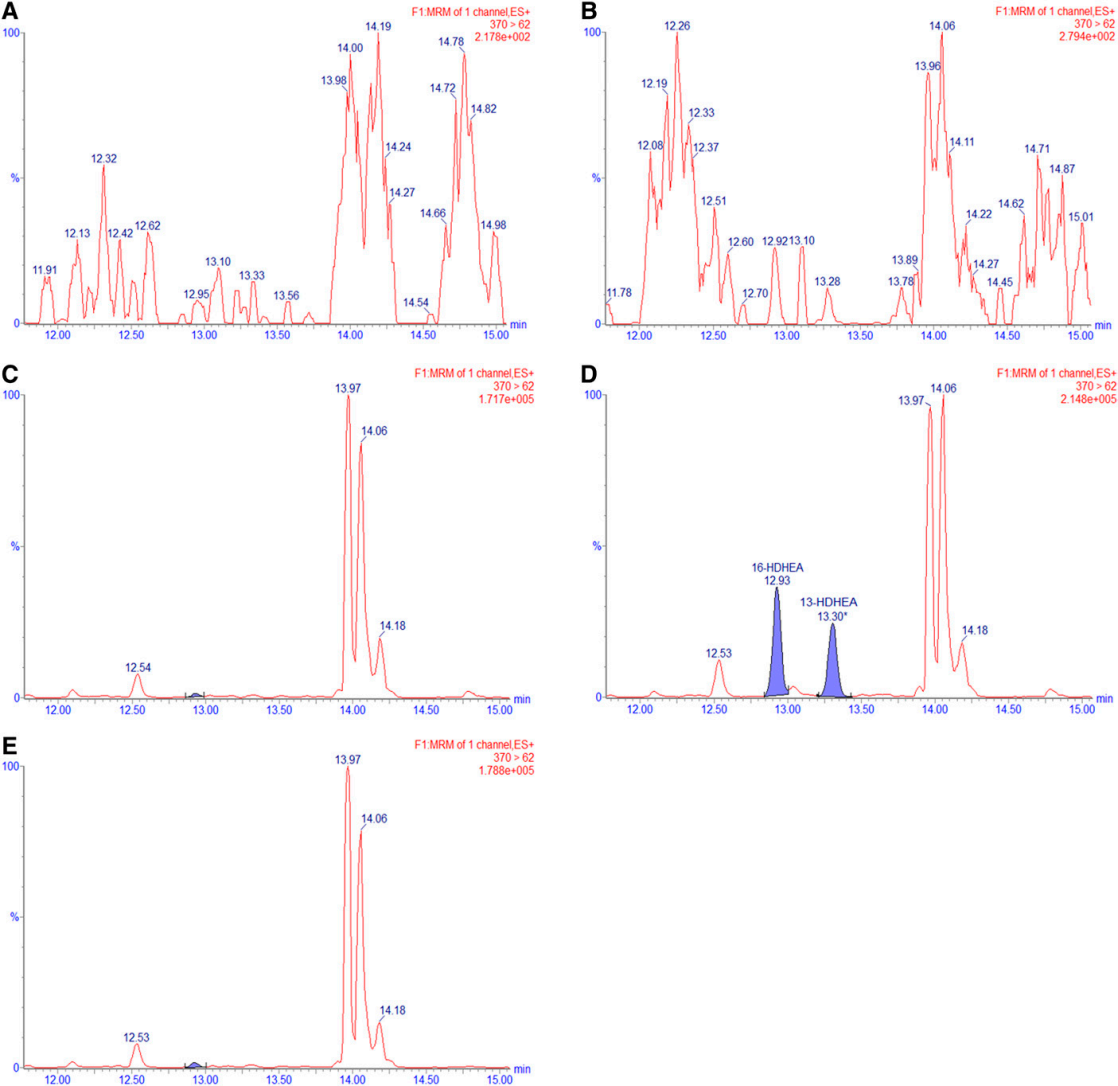
MRM chromatograms of *m/z* = 370 > 62 for the RAW264.7 macrophage extracts after 24 h of incubation. The macrophage cells were treated with a vehicle control (EtOH, PBS, and DMSO) (A), 1.0 μg/ml LPS (B), 10 μM of DHEA (C), 1.0 μg/ml LPS and 10 μM of DHEA (D), or 0.3 μM of celecoxib, 1.0 μg/ml LPS, and 10 μM of DHEA (E).

To investigate whether our findings from the hCOX-2 assay were also relevant in a murine cell-based system, we exposed LPS-stimulated RAW264.7 macrophages to DHEA and analyzed the cell lysates using our optimized UPLC-MS/MS method. After 30 min of incubation with 10 μM of DHEA and 24 h of stimulation with 1.0 μg/ml LPS, 13- and 16-HDHEA were detected as the main products in the cell extracts but not in the cell media. The products were quantified to be 43.2 (± 17.5) pmol/ml for 13-HDHEA and 36.3 (± 11.6) pmol/ml for 16-HDHEA (**Table 3**; supplemental Figs. S11, S12). No product was detected in the control incubations in which LPS and/or DHEA were not added (**Fig. 6**, Table 3). Additionally, to explore a potential time-dependent effect of LPS stimulation, we showed that the formation of 13- and 16-HDHEA was not significantly affected when the cells were first stimulated by LPS to upregulate COX-2 expression before the incubation with DHEA (supplemental Fig. S13). We also quantified the amount of DHEA that was present in the cell extracts at various time points, showing that only a limited amount of the 10 μM of DHEA was taken up by the cells but was chemically stable in medium without cells (supplemental Fig. S14) and that the highest amounts of 13- and 16-HDHEA were found after 24 h (supplemental Fig. S15).

**TABLE 3.** Concentrations of 13- and 16-HDHEA in 100 μl of the RAW264.7 macrophage extracts and concentrations of PGE_2_ in cell medium

Incubation Conditions	13-HDHEA (pmol/ml)	16-HDHEA (pmol/ml)	PGE_2_ (pmol/ml)
Vehicle control	N.D.	N.D.	0.0513 (± 0.0147)
DHEA (10 μM)	0.4 (± 0.4)	0.8 (± 0.4)	0.0899 (± 0.0126)
LPS (1.0 μg/ml)	N.D.	N.D.	2.0717 (± 0.0325)
DHEA (10 μM), LPS (1.0 μg/ml)	36.3 (± 11.6)	43.2 (± 17.5)	0.9229 (± 0.2111)
DHEA (10 μM), LPS (1.0 μg/ml), celecoxib (0.3 μM)	1.0 (± 0.5)	1.7 (± 0.4)	0.1245 (± 0.0244)

Data are presented as the mean from three independent experiments containing biological duplicates, with standard error of the mean shown in parentheses. N.D., not detected.

Clearly, 13- and 16-HDHEA formation in our macrophage model was caused by the combined incubation with 10 μM of DHEA and stimulation with 1.0 μg/ml LPS, suggesting that these products originated from a COX-2 dependent mechanism. Further evidence was provided by blocking the catalytic activity of COX-2 using 0.3 μM of the specific COX-2 inhibitor, celecoxib (IC_50_ 0.07 μM) (33, 42–44). The addition of celecoxib resulted in the complete loss of intracellular 13- and 16-HDHEA (Fig. 6) and of PGE_2_. Interestingly, PGE_2_ formation was decreased when LPS-stimulated cells were treated with DHEA (Table 3), confirming previously published data from our laboratory (18).

## DISCUSSION

In this study, we have developed an LC-HRMS assay to identify novel hCOX-2 products, which we validated using three different PUFAs (35, 36, 39, 41, 45, 46). Next, we demonstrated enzymatic conversion of neutral lipids, including the endocannabinoid, anandamide, and identified PGE_2_-EA, PGD_2_-EA, and 11- and 15-HETE-EA to further validate our methodology (2, 5, 10, 11, 13, 14). Interestingly, LC-HRMS data showed that hCOX-2 also oxygenates EPEA and DHEA. To the best of our knowledge, the present study is the first to report that DHEA and EPEA serve as COX-2 substrates, also providing evidence for a set of previously unknown lipid metabolites, such as PG_3_-EA, 11-, 14-, and 18-HEPE-EA, and 13- and 16-HDHEA. The newly detected compounds were identified based on the mass fragmentation pattern and the isotopic ratio, which were in correspondence with the proposed structure. The formation of PGE_3_-EA, 11-HEPE-EA, and 13- and 16-HDHEA was confirmed using synthetically derived congeners that displayed identical LC retention and mass fragmentation.

During validation of our LC-HRMS method, we found that both PUFA and endocannabinoid structures have the tendency to easily lose water during ionization, leading to a high abundance of [M+H–H_2_O]^+^ ions for the hydroxylated endocannabinoid products. This was found to be an inherent property associated with PUFA and endocannabinoid structures, and has been obtained in many studies before (7, 13, 36, 40, 41), especially for monohydroxylated products of the *N*-acylethanolamine derivatives. In those cases, no [M+H]^+^ ion peak was observed. Decreasing the temperature of the ion source did not decrease in-source fragmentation of the hydroxylated endocannabinoid products. Alternatively, a sodium adduct [M+Na]^+^ was detected in the positive ionization mode, and a formate adduct [M+CHO_2_]^–^ in the negative ionization mode. These ions allowed us to perform fragmentation experiments on the hydroxylated AEA, EPEA, and DHEA products.

In the LC-HRMS measurement of the hCOX-2-treated EPEA, we found three hydroxylated HEPE-EA products, i.e., 11-, 14-, and 18-HEPE-EA, whereas only two hydroxylated products of EPA were observed, i.e., 11- and 14-HEPE (35). Similarly, two DHEA-derived products were found, i.e., 13- and 16-HDHEA, whereas only one hydroxylated product of DHA was observed, i.e., 13-HDHA (35). The origin of these extra hydroxylated products in the *N*-acylethanolamine-derived PUFAs needs to be further investigated.

Next, we developed an UPLC-MS/MS method for the quantitative analysis of PGE_3_-EA, HEPE-EAs, and 13- and 16-HDHEA. Whereas our HPLC combined with Orbitrap HR-MS analysis proved to be a powerful tool to identify new COX-2-derived products, it was insufficient in achieving chromatographic separation and quantitatively analyzing the products. After achieving baseline separation of the products using UPLC, we were able to accurately quantify the enzymatic production of PGE_3_-EA and 13- and 16-HDHEA to be 0.5–1.5%. Although exact quantification of the HEPE-EAs was prohibited by the auto-oxidation of EPEA, we estimate the production of the HEPE-EAs to be in the same order as the formation of the other products. In summary, it was concluded that only a small fraction of the DHEA or EPEA is metabolized to 13- and 16-HDHEA or PGE_3_-EA, respectively.

Various earlier studies showed anti-inflammatory and anti-cancerous properties of 15-LOX or CYP450 metabolites of DHEA (23, 24, 47). Additionally, evidence was found that DHEA interacts with COX-2, resulting in interesting anti-inflammatory effects in vitro (18, 26). Therefore, we investigated to determine whether the COX-2-mediated conversion of DHEA also occurs in cells. Here, RAW264.7 macrophages were only stimulated with 1.0 μg/ml LPS, only incubated with 10 μM of DHEA, or incubated with 10 μM of DHEA 30 min prior to stimulation with 1.0 μg/ml LPS. In short, LPS activation stimulates the expression of COX-2 and induces an inflammatory response in the macrophages (18, 48–50). We used a DHEA preincubation of 30 min on the macrophages before the addition of LPS, which is similar to the incubation methodology described in Meijerink et al. (18). These conditions were chosen to ensure an identical incubation methodology compared with our previous study where we posed the hypothesis that DHEA could be a potential COX-2 substrate. To explore the time effect of LPS stimulation, control studies were performed, which showed that stimulation with 1.0 μg/ml LPS prior to the 24 h incubation with 10 μM of DHEA does not lead to higher concentrations of the products (supplemental Fig. S13). Similarly, product formation was shown to be the highest 24.5 h after DHEA incubation (which equals to 24 h after LPS stimulation) (supplemental Fig. S15).

We showed that 13- and 16-HDHEA products were formed when incubating the macrophages with both DHEA and LPS, and showed that there was no product formation when only one of the two compounds was added to the cells. In addition, when the selective COX-2 inhibitor, celecoxib, was added, no HDHEAs were detected, which further proved that 13- and 16-HDHEA are formed by a COX-2-dependent mechanism (Fig. 6, Table 3). Finally, a decrease in PGE_2_ formation was observed when treating the stimulated macrophages with 10 μM of DHEA; that PGD_2_ also decreases was already shown earlier (18). This observation indicates that the conversion of DHEA by COX-2 at least partly competes with the conversion of endogenous AA into PGs, and further supports the anti-inflammatory behavior linked to DHEA.

In the macrophage incubation experiment, we found that, after 24 h incubation, the macrophages produced 36.3 (± 11.6) pmol/ml of 13-HDHEA and 43.2 (± 17.5) pmol/ml of 16-HDHEA (Table 3). This is comparable to the amount of epoxidated products of DHEA formed by CYP450 in BV-2 microglia cells (24). Specifically, CYP450 forms between 3 and 10 pmol per 10^6^ BV-2 microglia cells of the various epoxidated isomers (24), and we find 7.3 (± 2.3) pmol of 13-HDHEA per 10^6^ RAW macrophage cells and 8.6 (± 3.5) pmol of 16-HDHEA per 10^6^ RAW macrophage cells. Although obtained by different laboratories and using different models, these apparent comparable amounts indicate that the products are formed in the same order of magnitude.

Furthermore, it must be taken into account that only 1–2% of the added 10 μM of DHEA was detected in the macrophages (supplemental Fig. S14). This is most likely due to limited uptake of the DHEA, degradation via NAAA and FAAH, and conversion into novel metabolites via various lipoxygenases and CYP450s. Studies with radiolabeled DHEA in immortalized fetal mesencephalic cells also showed that the uptake of DHEA was limited, and that the uptake of DHEA is driven by its FAAH-dependent hydrolysis (51). Therefore, we expect that the reported values of 13- and 16-HDHEA most likely are an underestimation of the real biosynthetic capacity of the macrophage model used. In addition to this, it must be pointed out that DHEA itself can also modulate several specific anti-inflammatory activities during incubation. For example, it has been shown that DHEA inhibits PG formation (18, 25) and is able to bind several endocannabinoid-related receptors, like CB2, TRPV1 (50), and GPR-110 (29); although for GPR-110, it is unknown whether this receptor is present in macrophages. Whether these indirect effects of DHEA interfere with the synthesis of 13- and 16-HDHEA in our study is not known.

Finally, the fact that 13- and 16-HDHEA were found in both the hCOX-2 assay and the murine RAW264.7 macrophages suggests that DHEA oxygenation by COX-2 is conserved between both human COX-2 and murine COX-2. Further studies are warranted to reveal the presence of the hydroxylated DHEA and EPEA products in blood and tissues and to investigate the biological effects of the novel oxygenated DHEA products. Based on the strong anti-tumorigenic and anti-inflammatory properties of previously identified epoxidated and hydroxylated DHEA-derived products (23, 24, 47), it is tempting to speculate that these newly detected compounds play a role in inflammatory processes, in already limited concentrations.

In conclusion, we have developed a new cell-free hCOX-2 metabolite identification and quantification method. Using this method, we have identified previously unknown COX-2 metabolites of DHEA and EPEA in hCOX-2 incubations and extended these findings to LPS-stimulated RAW264.7 macrophages. Future work in our laboratory will investigate the biological effects of these new DHEA and EPEA metabolites, characterize the binding properties of DHEA to COX-2, and further focus on the uptake and metabolism of DHEA.

## Supplementary Material

Supplemental Data
