# Supplementary material for: Novel COX-2 products of n-3 polyunsaturated fatty acid-ethanolamine-conjugates identified in RAW264.7 macrophages
Source: J Lipid Res. 2019 Aug 27;60(11):1829–40. doi: 10.1194/jlr.M094235 (PMC6824491; doi:10.1194/jlr.M094235)
Supplement: Supplemental Data [file supp_60_11_1829__index.html]

Novel COX-2 products of n-3 polyunsaturated fatty acid-ethanolamine-conjugates identified in RAW 264.7 macrophages — Novel COX-2 products of n-3 polyunsaturated fatty acid-ethanolamine-conjugates identified in RAW264.7 macrophages — Supplemental Data 

# Novel COX-2 products of *n*-3 polyunsaturated fatty acid-ethanolamine-conjugates identified in RAW264.7 macrophages

## Supplemental Data

- Supplemental Information (.pdf, 4.1 MB) - Extensively revised supplemental information containing synthetic procedures and characterization for the reference standards, additional characterization and analysis of metabolites.
