# Supplementary material for: Novel COX-2 products of n-3 polyunsaturated fatty acid-ethanolamine-conjugates identified in RAW264.7 macrophages
Source: J Lipid Res. 2019 Aug 27;60(11):1829–40. doi: 10.1194/jlr.M094235 (PMC6824491; doi:10.1194/jlr.M094235)
Supplement: Supplemental Data [file 10.1194_M094235_jlr.M094235-1.pdf]

Supplementary Information

for

Novel COX-2 products of *n*-3 polyunsaturated fatty acid-ethanolamine-conjugates identified in RAW264.7 macrophages

Ian de Bus[1,2], Han Zuilhof[1,3,4], Renger Witkamp[2], Michiel Balvers[2,]*, Bauke Albada[1,]*

1) Laboratory of Organic Chemistry, Wageningen University & Research,
Stippeneng 4, 6708 WE, Wageningen, the Netherlands
2) Nutrition and Pharmacology Group, Division of Human Nutrition, Wageningen University & Research,
Stippeneng 4, 6708 WE, Wageningen, the Netherlands
3) School of Pharmaceutical Sciences and Technology, Tianjin University,
92 Weijin Road, Tianjin, P.R. China.
4) Department of Chemical and Materials Engineering, King Abdulaziz University,
Jeddah, Saudi Arabia.

Synthetic Procedures for the Standards

Synthesis of the PGE_3-EA and 11-HEPE-EA standards

For the synthesis of the analytical standards of PGE_3-EA and 11-HEPE-EA, ethanolamine was coupled to PGE_3 and 11-HEPE using a slightly adapted version of a previously described coupling methodology (1). Specifically, 100 µg of PGE_3 and 11-HEPE in EtOH were evaporated to dryness by co-evaporation with 2 mL DCM, after which the compounds were dissolved in 1 mL dry DCM. Subsequently, a solution of 59.3 µL freshly distilled triethylamine in 10 mL dry DCM, and 47.3 µL isobutyl chloroformate in 10 mL dry DCM were prepared under an argon atmosphere. From these stock solutions 98 µL was added to the PGE_3 and 108 µL was added to the 11-HEPE. Both reactions were stirred at room temperature for 60 min under argon atmosphere to form mixed anhydrides. Next, the mixed anhydrides were reacted with 98 µL or 108 µL of a reaction solution containing 46.6 µL freshly distilled triethylamine and 20.2 µL ethanolamine in 10 mL dry DCM. The reaction mixtures were then stirred on ice for 16 h under argon atmosphere. Hereafter the reactions were evaporated under a stream of nitrogen and dissolved in 30/70 water/ACN mixture for preparative HPLC purification. The products were purified and quantified using preparative and analytical HPLC (*vide infra*). We obtained 28.9 µg PGE_3-EA (26% yield) and 26.6 µg of 11-HEPE-EA (23% yield).

Synthesis of 11-HEPE-EA-d_4 standard

For the synthesis of the analytical standard of 11-HEPE-EA-d_4, ethanolamine-d_4 was coupled to 11-HEPE using a slightly adapted version of a previously described coupling methodology (1). Specifically, 50 µg of 11-HEPE in EtOH was evaporated to dryness by co-evaporation with 2 mL DCM, after which the compound was dissolved in 1 mL dry DCM. Subsequently a solution of 59.3 µL freshly distilled triethylamine in 10 mL dry DCM, and 47.3 µL isobutyl chloroformate in 10 mL dry DCM were prepared under an argon atmosphere. From these stock solutions 54 µL was added to the reaction mixture, and the reaction was stirred at room temperature for 60 min under argon atmosphere to form the mixed anhydride. Next, the mixed anhydride was reacted with 54 µL of a reaction solution containing 23.3 µL freshly distilled triethylamine and 10.1 µL ethanolamine in 5 mL dry DCM. The reaction mixture was stirred on ice for 16 h under argon atmosphere. Hereafter the reaction mixture was evaporated under a stream of nitrogen and dissolved in 30/70 water/ACN mixture for preparative HPLC purification. The product was purified and quantified using preparative and analytical HPLC (*vide infra*). We obtained 4.4 µg of 11-HEPE-EA (8% yield).

Synthesis of the 13-HDHEA(-d_4) and 16-HDHEA standards

For the synthesis of the analytical standards 13-HDHEA and 16-HDHEA, and the internal standard 13-HDHEA-d_4 ethanolamine or ethanolamine-d_4 was coupled to 13-HDHA and/or 16-HDHA, using an optimised coupling methodology (1). This resulted in quantitative coupling of ethanolamine to the fatty acids, and limited the loss of product during the workup. 100 µg of 13-HDHA or 16-HDHA in EtOH were evaporated to dryness by co-evaporation with 2 mL DCM. The HDHA compounds were then re-dissolved in 1 mL dry DCM, and subsequently a solution of 59.3 µL freshly distilled triethylamine in 10 mL dry DCM, and 47.3 µL isobutyl chloroformate in 10 mL dry DCM were prepared under an argon

atmosphere. From these stock solutions 100 µL was added to the reaction mixture, and the reaction was stirred at room temperature for 60 min under argon atmosphere to form the mixed anhydride. Next, the mixed anhydride was reacted with 100 µL of a reaction solution of 46.6 µL freshly distilled triethylamine and 20.2 µL ethanolamine or ethanolamine-d_4 in 10 mL dry DCM. The reaction mixture was stirred on ice for 16 h under argon atmosphere, after which the product was evaporated to dryness and reconstituted in 30/70 water/ACN for preparative HPLC purification and quantification with analytical HPLC (*vide infra*). After purification we obtained 27.5 µg of 13-HDHEA-d_4 (24% yield), 13.0 µg 13-HDHEA (12% yield), and 7.8 µg 16-HDHEA (7% yield).

HPLC purification and quantification of synthesised standards

The products were purified using preparative HPLC on a Zorbax Eclipse C18 semi preparative column, 5 µ column of 9.4 × 250 mm (Agilent, the Netherlands) using an isocratic run of 30/70 Ultrapure water/ACN with 0.1% FA for the HEPE-EA and HDHEA standards and an isocratic run of 70/30 Ultrapure water/ACN with 0.1% FA for the PGE_3-EA standard. The same HPLC methodology was used to quantify the standards, by measuring the area under the curve of the UV-absorption at 210 or 240 nm on an analytical Zorbax Eclipse C18 column, 5 µ column of 4.6 × 250 mm. The UV-absorption of was found not be influenced by the ethanolamine group that was attached during the synthesis.

NMR spectroscopy

The reference compounds and synthesised standards were evaporated to dryness using a speedyvac concentrator (Salm and Kipp, Breukelen, the Netherlands), and again dissolved in 200 µL $CDCl_3$. The spectra were recorded on a 600 MHz NMR machine *(vide infra)*.

Additional MS-analysis of COX-2 related oxidation products

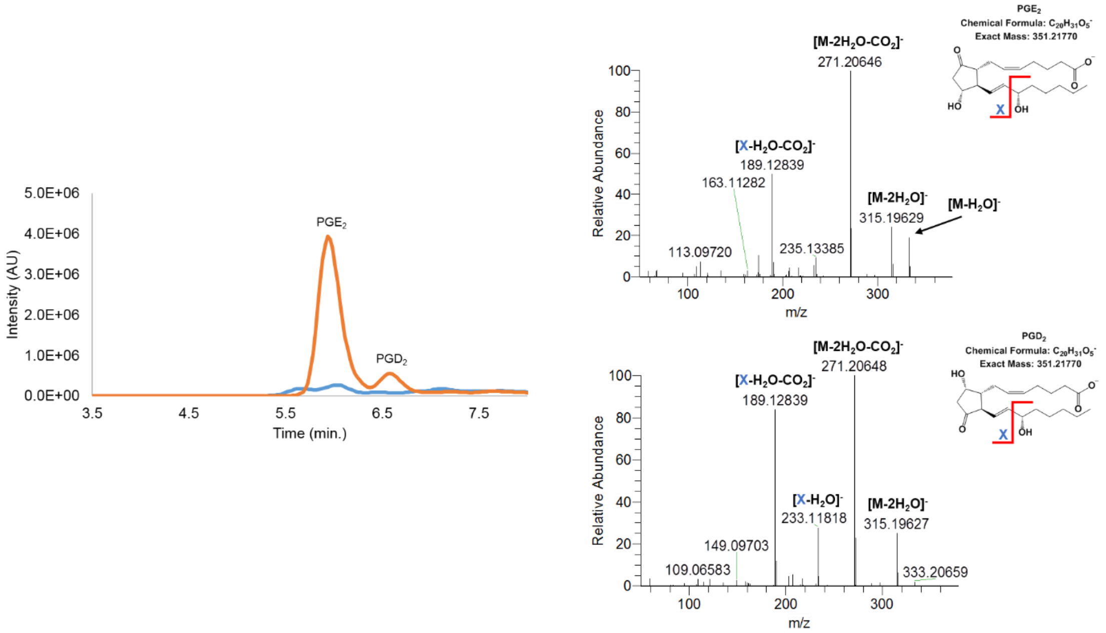

Supplemental Figure S1 – Extracted ion chromatogram of *m/z* = 351.21–351.22, which is assigned to [M-H]⁻ of PGE_2 and PGD_2. The orange trace in the chromatogram shows the products of the hCOX-2 assay with AA, the blue trace shows the auto-oxidation products of AA when no enzyme is present. Mass fragmentation spectra of both hCOX-2 products were obtained using a collision energy of 20.0 eV in the HCD chamber. PGE_2 fragmentation spectrum was the average from 5.82–6.33 min. and PGD_2 fragmentation spectrum is the average from 6.60–7.19 min.

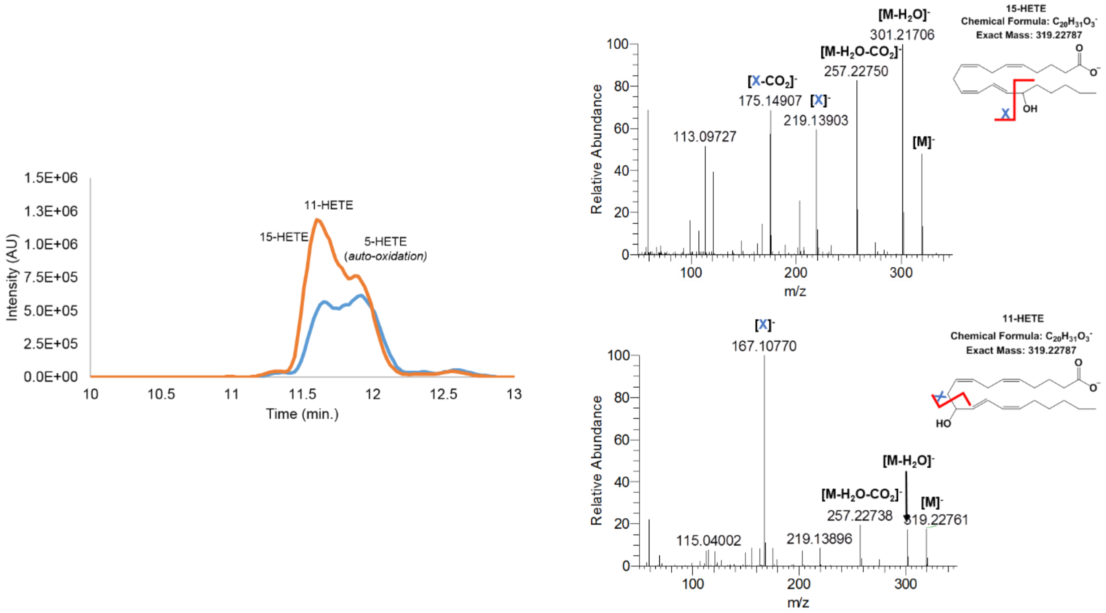

Supplemental Figure S2 – Extracted ion chromatogram of *m/z* = 319.21–319.23, which is assigned to [M-H]⁻ of HETEs. The orange trace in the chromatogram shows the products of the hCOX-2 assay with AA, the blue trace shows the auto-oxidation products of AA when no enzyme is present. Mass fragmentation spectra of both hCOX-2 products were obtained using a collision energy of 20.0 eV in the HCD chamber. 15-HETE fragmentation spectrum was the average from 11.19–11.33 min. and 11-HETE fragmentation spectrum is the average from 11.47–11.97 min.

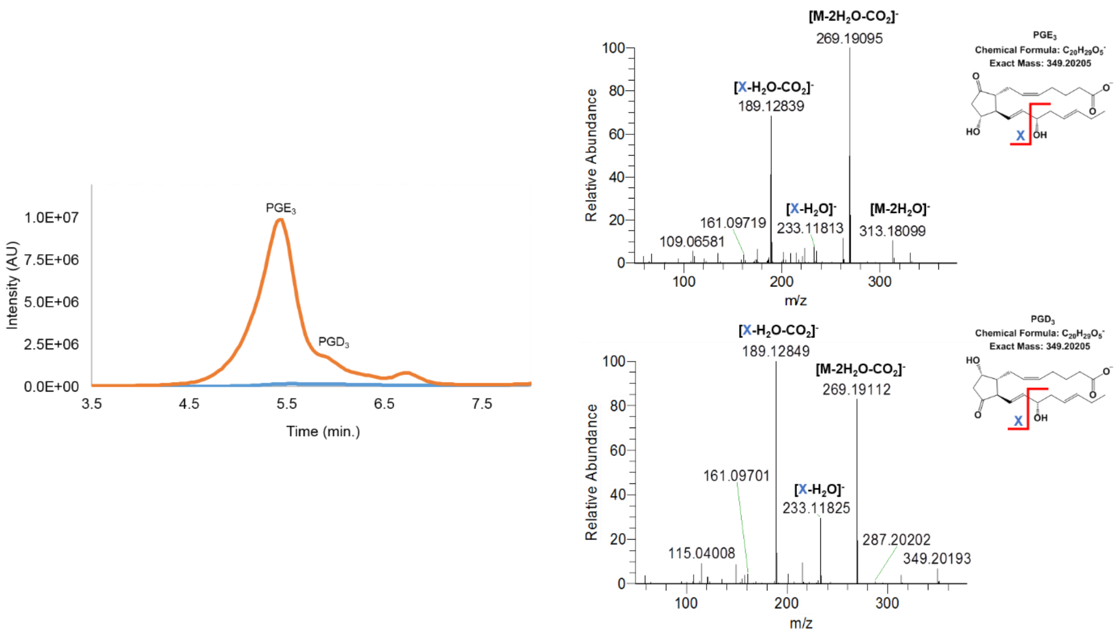

Supplemental Figure S3 – Extracted ion chromatogram of *m/z* = 349.19–349.21, which is assigned to [M-H]⁻ of PGE_3 and PGD_3. The orange trace in the chromatogram shows the products of the hCOX-2 assay with EPA, the blue trace shows the auto-oxidation products of EPA when no enzyme is present. Mass fragmentation spectra of both hCOX-2 products were obtained using a collision energy of 20.0 eV in the HCD chamber. PGE_3 fragmentation spectrum was the average from 5.03–5.52 min. and PGD_3 fragmentation spectrum is the average from 5.57–5.78 min.

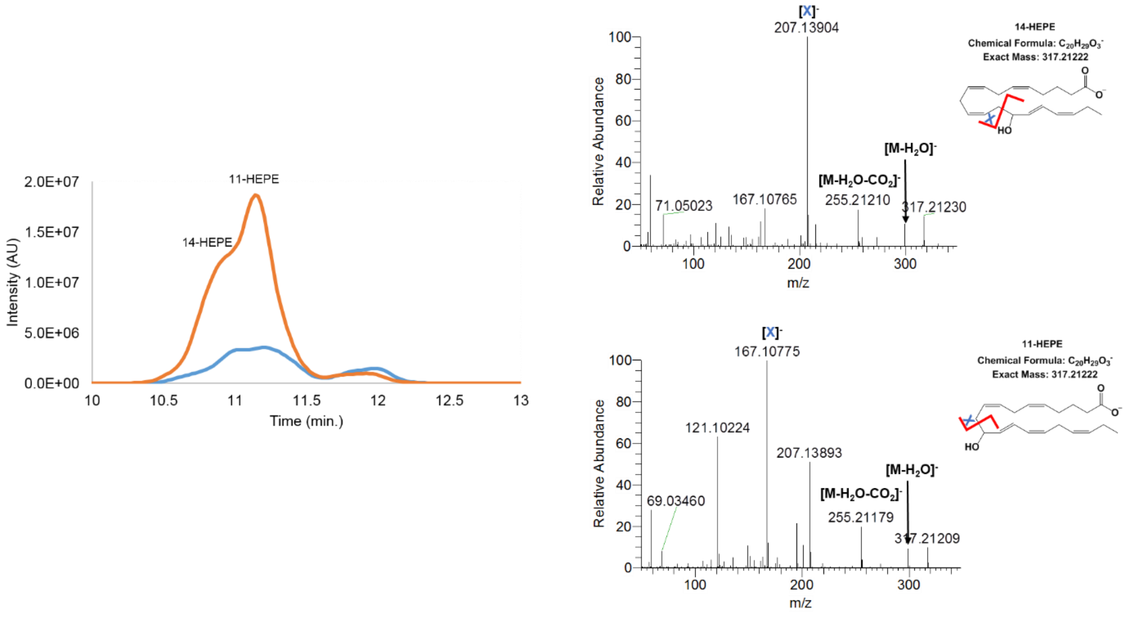

Supplemental Figure S4 – Extracted ion chromatogram of *m/z* = 317.21–317.22, which is assigned to [M-H]⁻ of HEPEs. The orange trace in the chromatogram shows the products of the hCOX-2 assay with EPA, the blue trace shows the auto-oxidation products of EPA when no enzyme is present. Mass fragmentation spectra of both hCOX-2 products were obtained using a collision energy of 20.0 eV in the HCD chamber. 14-HEPE fragmentation spectrum was the average from 10.93–10.98 min. and 11-HEPE fragmentation spectrum is the average from 11.14–11.28 min.

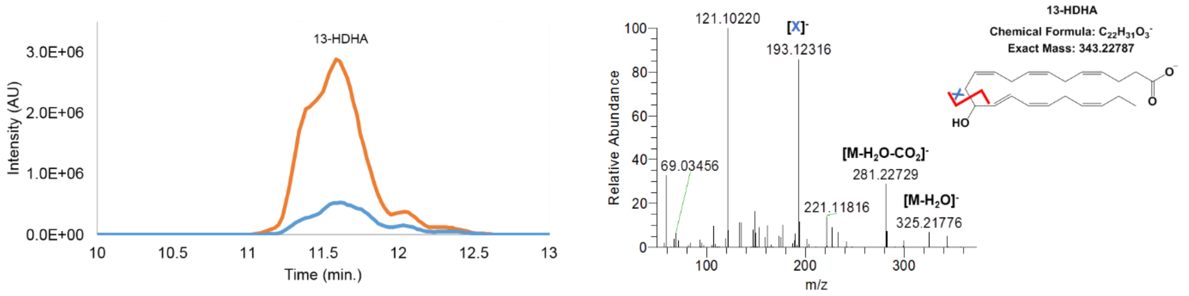

Supplemental Figure S5 – Extracted ion chromatogram of *m/z* = 343.22–343.24, which is assigned to $[M\text{-}H]^-$ of PGE_3 and PGD_3. The orange trace in the chromatogram shows the products of the hCOX-2 assay with DHA, the blue trace shows the auto-oxidation products of DHA when no enzyme is present. Mass fragmentation spectra of the hCOX-2 product was obtained using a collision energy of 20.0 eV in the HCD chamber. 13-HDHA fragmentation spectrum was the average from 11.43–11.70 min.

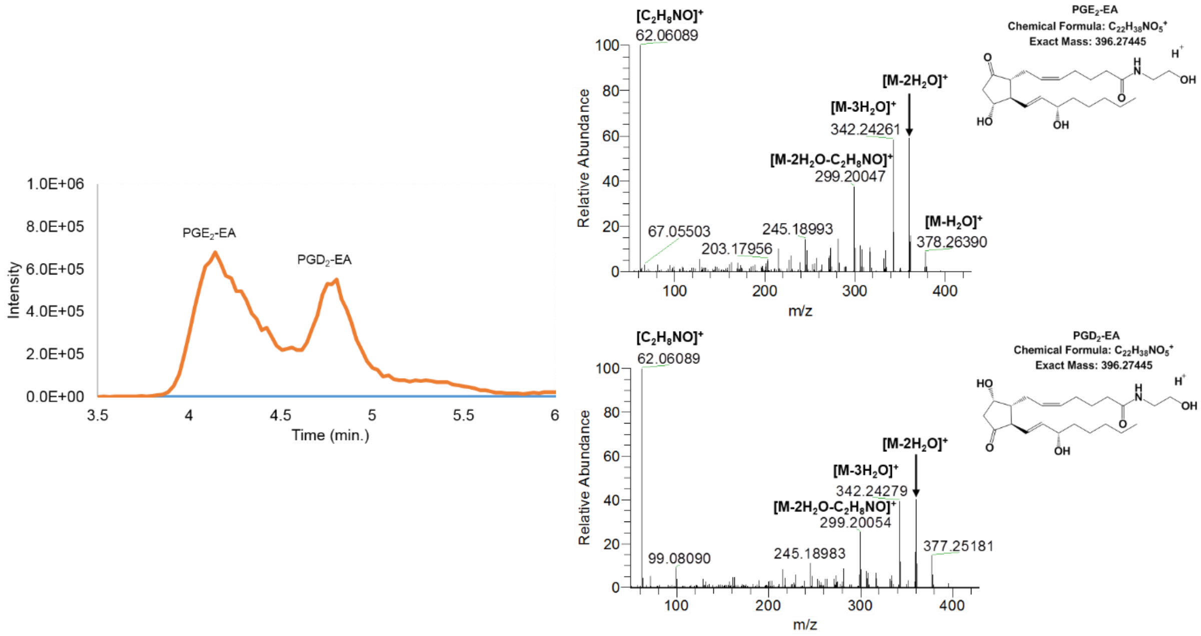

Supplemental Figure S6 – Extracted ion chromatogram of *m/z* = 396.26–396.29, which is assigned to $[M+H]^+$ of PGE_2-EA and PGD_2-EA. The orange trace in the chromatogram shows the products of the hCOX-2 with AEA, the blue trace shows the auto-oxidation products of AEA when no enzyme is present.. Mass fragmentation spectra of both hCOX-2 products were obtained using a collision energy of 20.0 eV in the HCD chamber. PGE_2-EA fragmentation spectrum was the average from 4.02–4.47 min. and PGD_2-EA fragmentation spectrum is from 4.80 min.

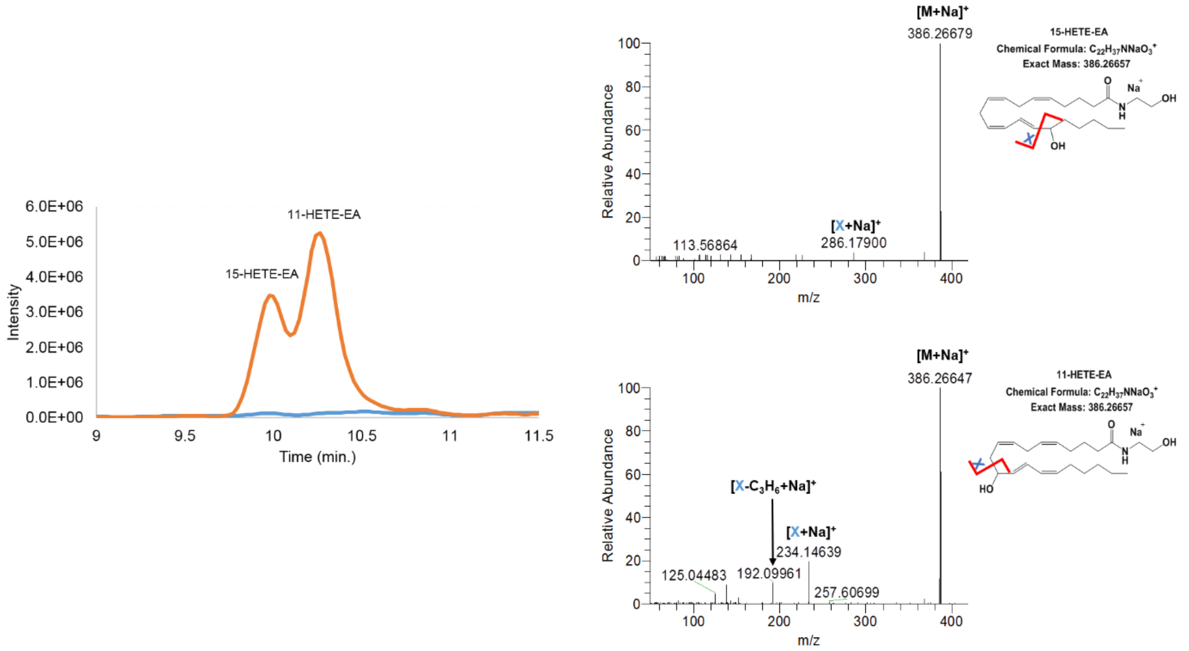

Supplemental Figure S7 – Extracted ion chromatogram of *m/z* = 346.26–346.29, which is assigned to $[M+H-H_2O]^+$ of HETE-EAs. The orange trace in the chromatogram shows the products of the hCOX-2 assay with AEA, the blue trace shows the auto-oxidation products of AEA when no enzyme is present.. Mass fragmentation spectra of both hCOX-2 products were obtained on the $[M+Na]^+$ adduct ion using a collision energy of 30.0 eV in the HCD chamber. 15-HETE-EA fragmentation spectrum was from 9.97 min. and 11-HETE-EA fragmentation spectrum is the average from 10.17–10.35 min.

LC-MS and MS/MS analysis of synthesized mono-hydroxylated ethanolamide standards

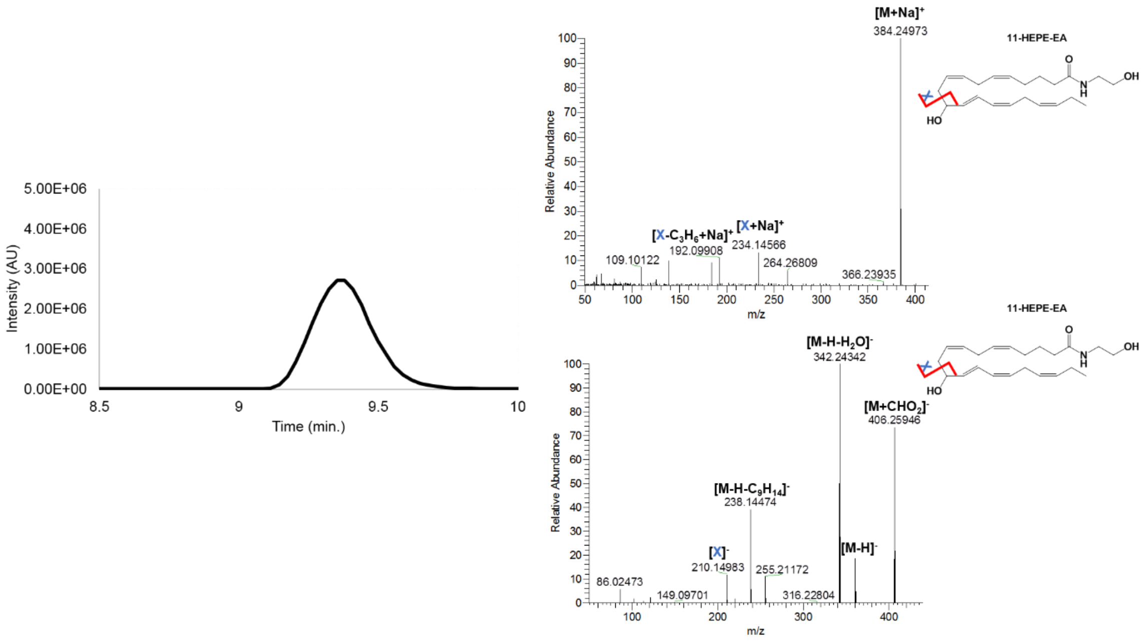

Supplemental Figure S8 – Extracted ion chromatogram of the chemically synthesized 11-HEPE-EA. The extracted ion chromatogram in black shows *m/z* = 344.25–344.27, which is assigned to $[M+H-H_2O]^+$. Mass fragmentation spectra of both the $[M+Na]^+$ adduct ion using a collision energy of 30.0 eV in the HCD chamber *(top)*, and the $[M+HCO_2]^-$ using a collision energy of 10.0 eV in the HCD chamber *(bottom)* are depicted. Mass fragmentation spectra depicted are averages of the entire chromatographic peak.

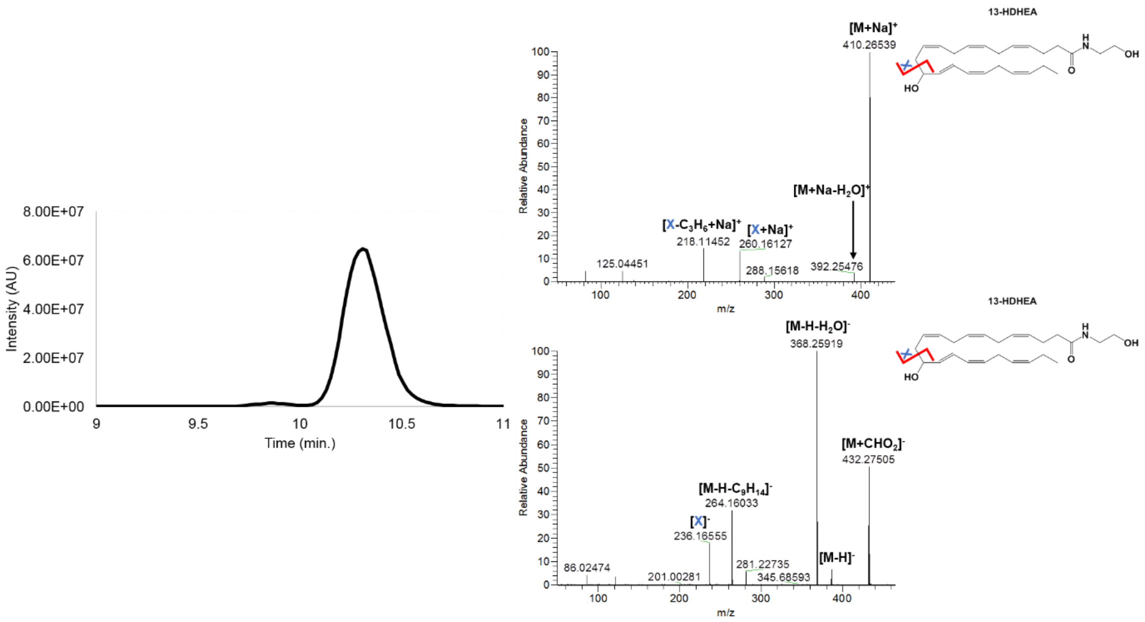

Supplemental Figure S9 – Extracted ion chromatogram of the chemically synthesized 13-HDHEA. The extracted ion chromatogram in black shows *m/z* = 370.26–370.28, which is assigned to $[M+H-H_2O]^+$. Mass fragmentation spectra of both the $[M+Na]^+$ adduct ion using a collision energy of 30.0 eV in the HCD chamber *(top)*, and the $[M+HCO_2]^-$ using a collision energy of 10.0 in the HCD chamber *(bottom)* are depicted. Mass fragmentation spectra depicted are averages of the entire chromatographic peak.

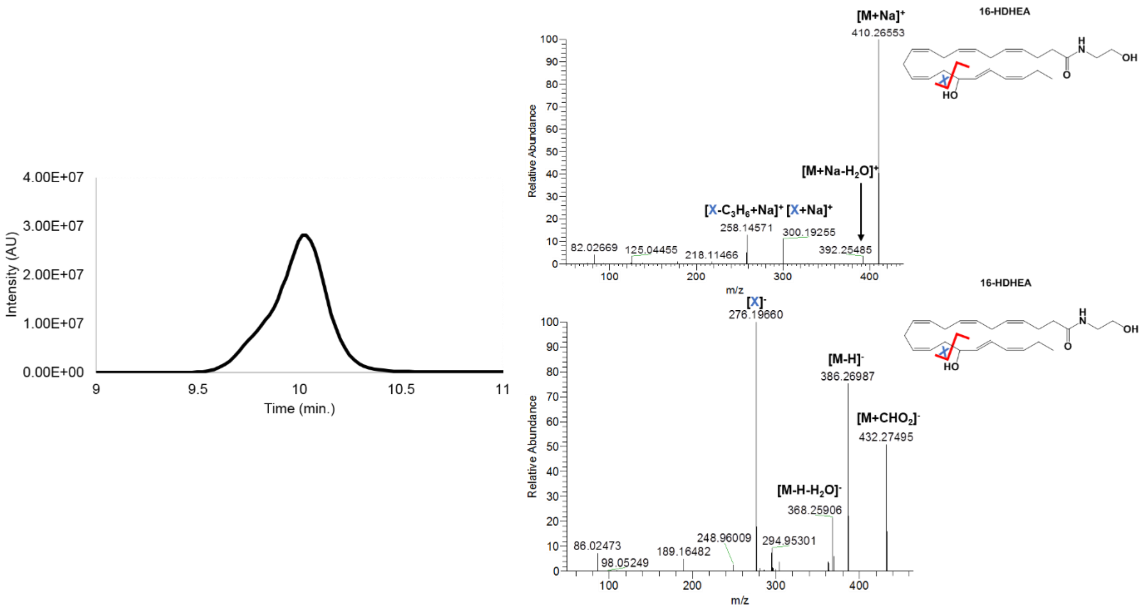

Supplemental Figure S10 – Extracted ion chromatogram of the chemically synthesized 16-HDHEA. The extracted ion chromatogram in black shows *m/z* = 370.26–370.28, which is assigned to $[M+H\text{-}H_2O]^+$. Mass fragmentation spectra of both the $[M+Na]^+$ adduct ion using a collision energy of 30.0 eV in the HCD chamber *(top)*, and the $[M+HCO_2]^-$ using a collision energy of 10.0 eV in the HCD chamber *(bottom)* are depicted. Mass fragmentation spectra depicted are averages of the entire chromatographic peak.

Calibration curves for 16- and 13-HDHEA

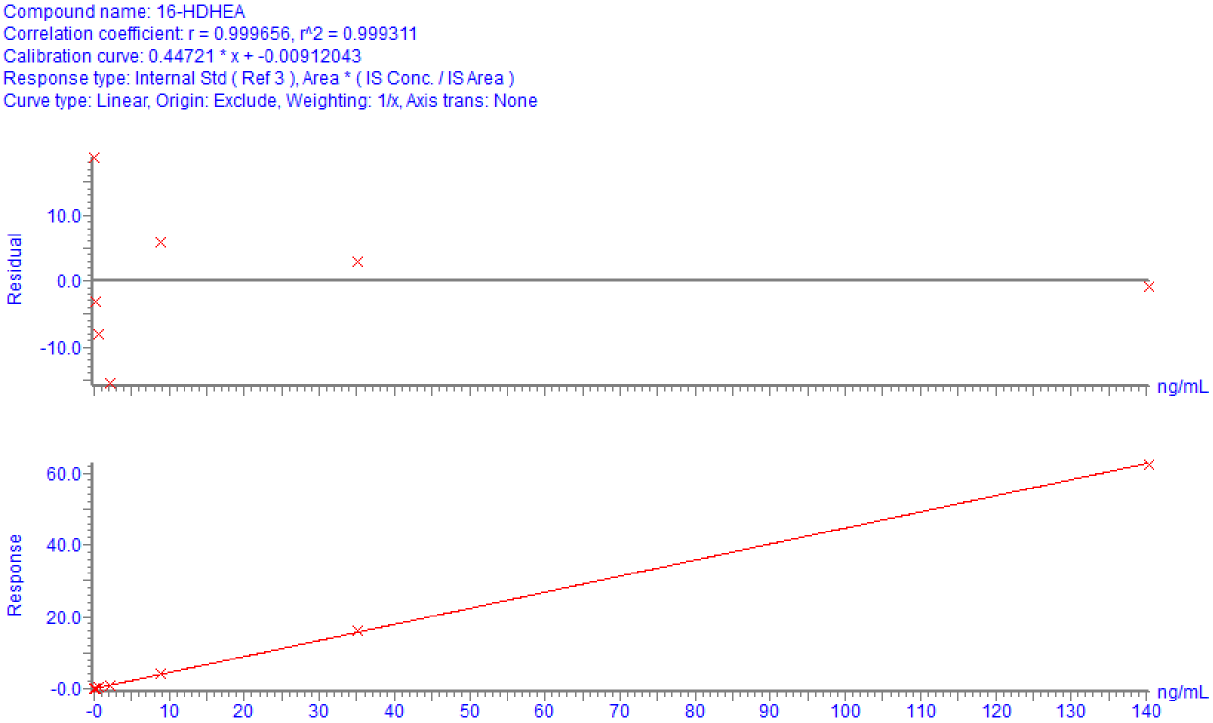

Supplemental Figure S11 – Representative calibration curve of 16-HDHEA, used for the quantification of 16-HDHEA compounds in the RAW264.7 assay.

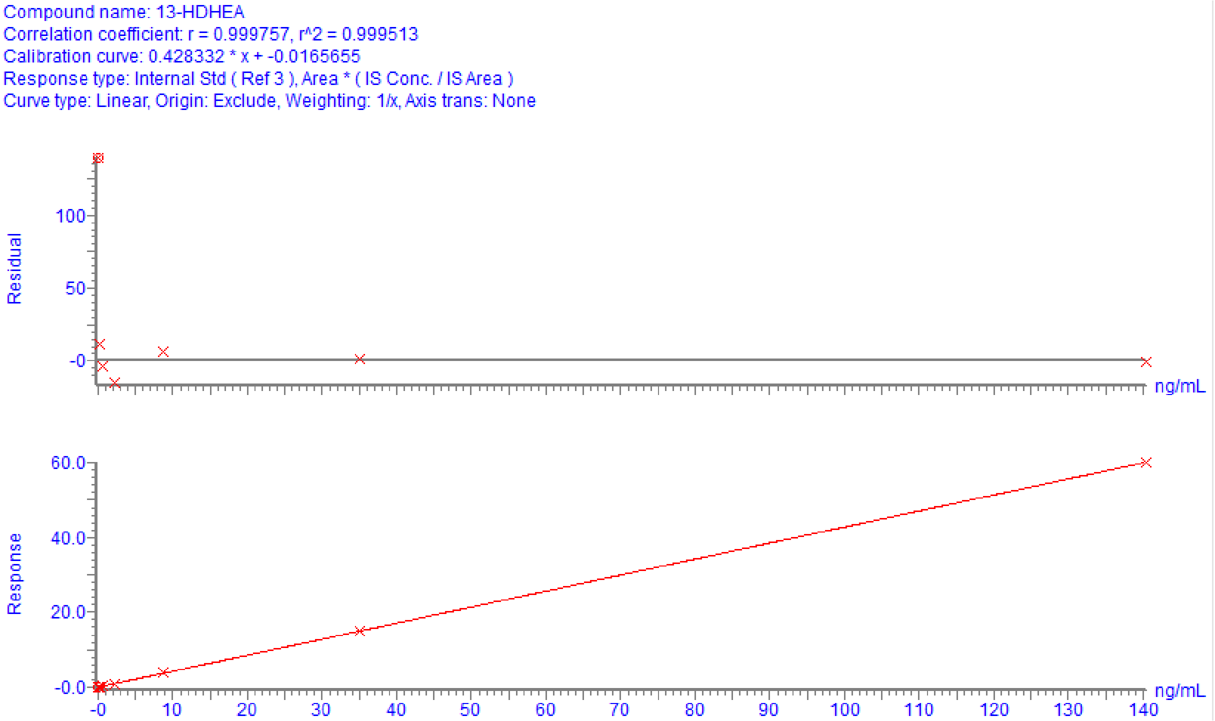

Supplemental Figure S12 – Representative calibration curve of 13-HDHEA, used for the quantification of 13-HDHEA compounds in the RAW264.7 assay.

Control experiments on RAW264.7 macrophages

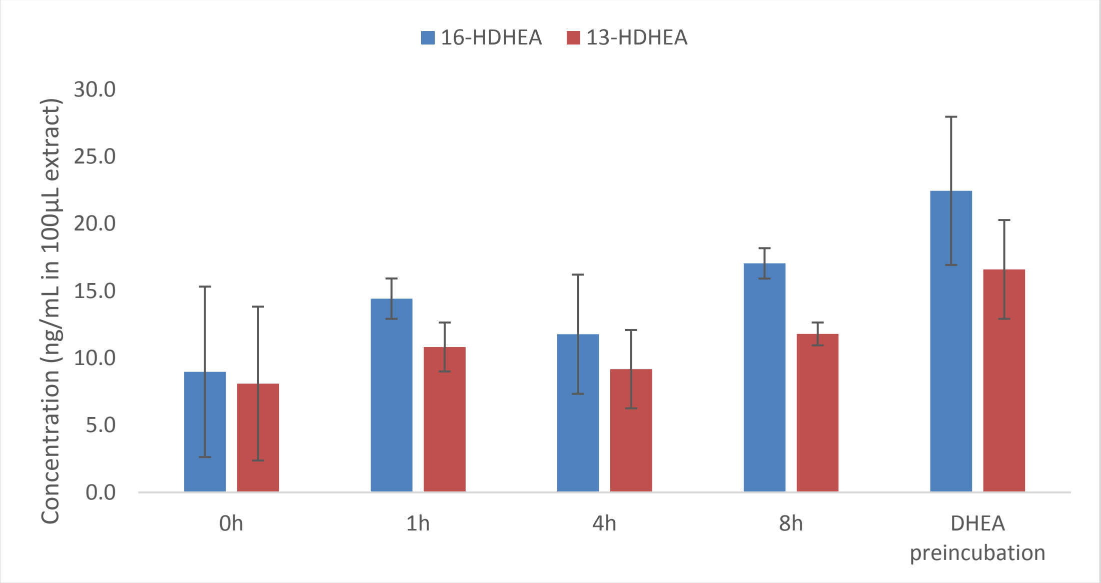

Supplemental Figure S13 – Production of 13- and 16-HDHEA by RAW264.7 macrophages 24 h after incubation with 10 µM DHEA. Stimulation was performed by adding 1.0 µg/mL LPS 0 h, 1 h, 4 h or 8 h to the cells prior to the DHEA incubation, or 30 min. after DHEA incubation for the DHEA pre-incubation experiment. Standard error bars obtained from biological duplicates.

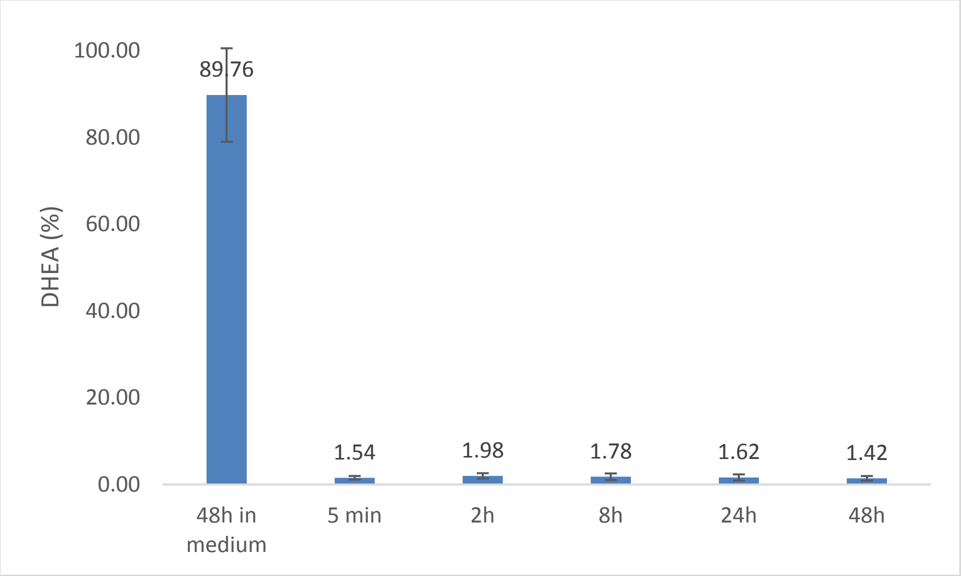

Supplemental Figure S14 – Quantification of DHEA (in % relative to the 10 µM DHEA that was added) in the RAW264.7 macrophage extracts at various time points. As control 10 µM DHEA was incubated for 48 h in medium without cells. All the cells were incubated with 10 µM DHEA 30 min. before stimulation with 1.0 µg/mL LPS. Standard error bars obtained from biological duplicates.

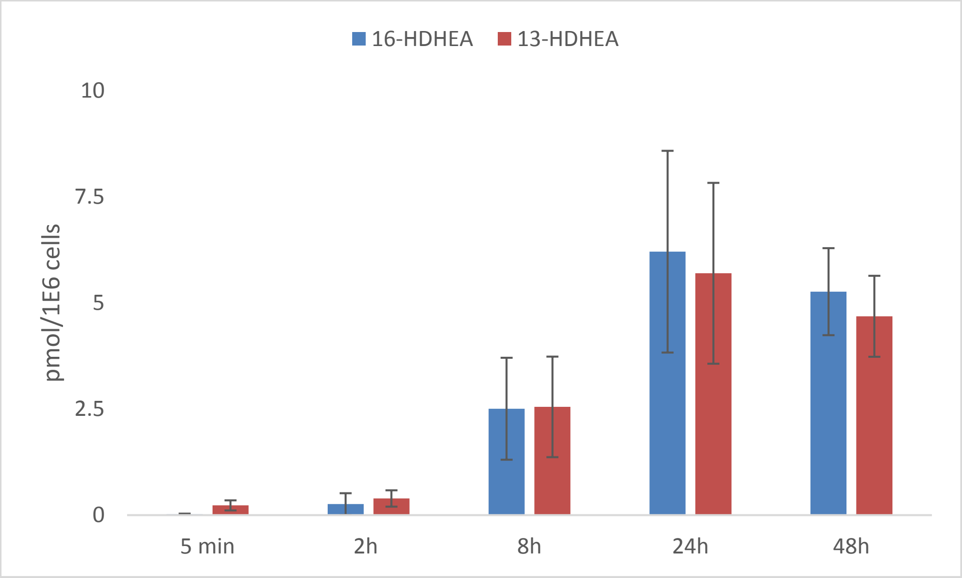

Supplemental Figure S15 – Production of 13- and 16-HDHEA by RAW264.7 macrophages as function of time. The cells were incubated with 10 µM DHEA 30 min. before the stimulation with 1.0 µg/mL LPS. 13- and 16-HDHEA were quantified at various times. Standard error bars obtained from biological duplicates.

Supplemental Table S1 – Optimised MRM transitions for the UPLC-MS/MS quantification of the compounds.

Compound	MRM transition	CE (eV)
DHEA	m/z = 372 $[M+H]^+ \rightarrow m/z$ = 62 $[C_2H_8NO]^+$	15
DHEA-d_4	m/z = 376 $[M+H]^+ \rightarrow m/z$ = 66 $[C_2H_4D_4NO]^+$	15
13-HDHEA	m/z = 370 $[M–H_2O+H]^+ \rightarrow m/z$ = 62 $[C_2H_8NO]^+$	15
13-HDHEA-d_4	m/z = 374 $[M–H_2O+H]^+ \rightarrow m/z$ = 66 $[C_2H_4D_4NO]^+$	15
EPEA	m/z = 346 $[M+H]^+ \rightarrow m/z$ = 62 $[C_2H_8NO]^+$	15
11-HEPE-EA	m/z = 344 $[M–H_2O+H]^+ \rightarrow m/z$ = 62 $[C_2H_8NO]^+$	15
11-HEPE-EA-d_4	m/z = 388 $[M+Na]^+ \rightarrow m/z$ = 238 $[C_{12}H_{18}D_4NO_2Na]^+$	25
PGE_3-EA	m/z = 416 $[M+Na]^+ \rightarrow m/z$ = 398 $[M–H_2O+Na]^+$	25
PGE_2-EA-d_4	m/z = 422 $[M+Na]^+ \rightarrow m/z$ = 404 $[M–H_2O+Na]^+$	25

Analysis of hydroxylated PUFA reference standards

A) UV-absorption spectrum of 100 µg/mL of the reference compounds.
B) ^{1}H-NMR of the references.
C) TOCSY-NMR of the references.
D) HSQC-NMR of the references.

11-HEPE

A)

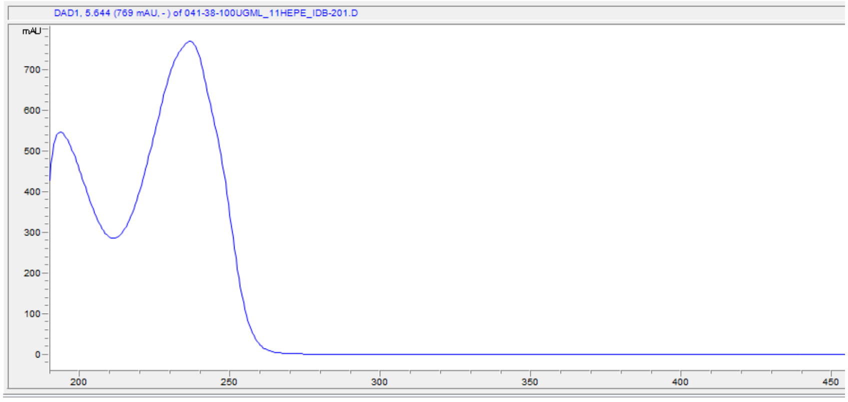

Supplemental Figure S16 – UV-absorption spectrum of 100 µg/mL 11-HEPE.

B)

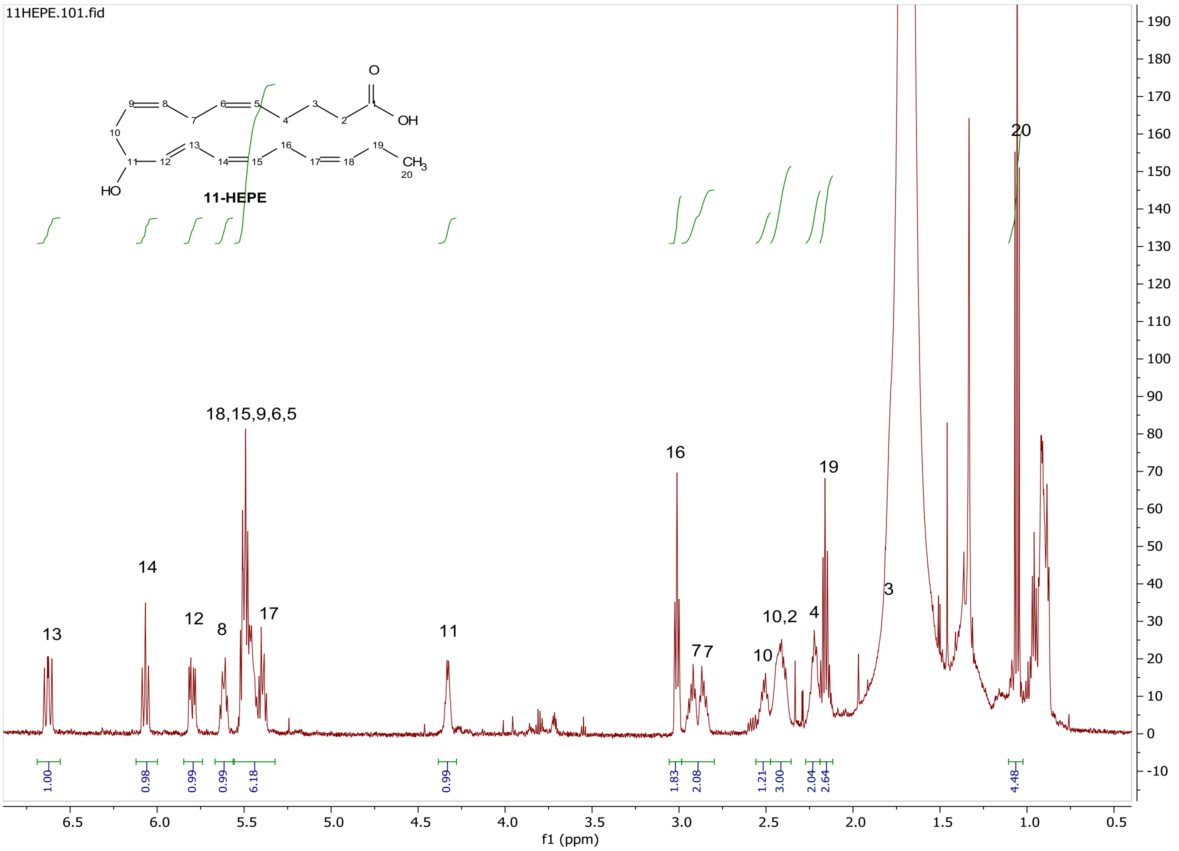

Supplemental Figure S17 – ^{1}H NMR of the reference compound 11-HEPE (600 MHz, chloroform-*d*) δ 6.63 (dd, *J* = 15.2, 11.0 Hz, 1H), 6.07 (t, *J* = 10.9 Hz, 1H), 5.80 (dd, *J* = 15.3, 6.3 Hz, 1H), 5.69–5.56 (m, 1H), 5.55–5.34 (m, 6H), 4.33 (q, *J* = 6.5 Hz, 1H), 3.01 (t, *J* = 7.5 Hz, 2H), 2.89 (ddt, *J* = 44.8, 15.3, 7.2 Hz, 2H), 2.51 (dt, *J* = 14.6, 7.4 Hz, 1H), 2.47–2.36 (m, 3H), 2.23 (q, *J* = 7.7 Hz, 1H), 2.16 (p, *J* = 7.7 Hz, 2H), 1.06 (t, *J* = 7.5 Hz, 3H).

C)

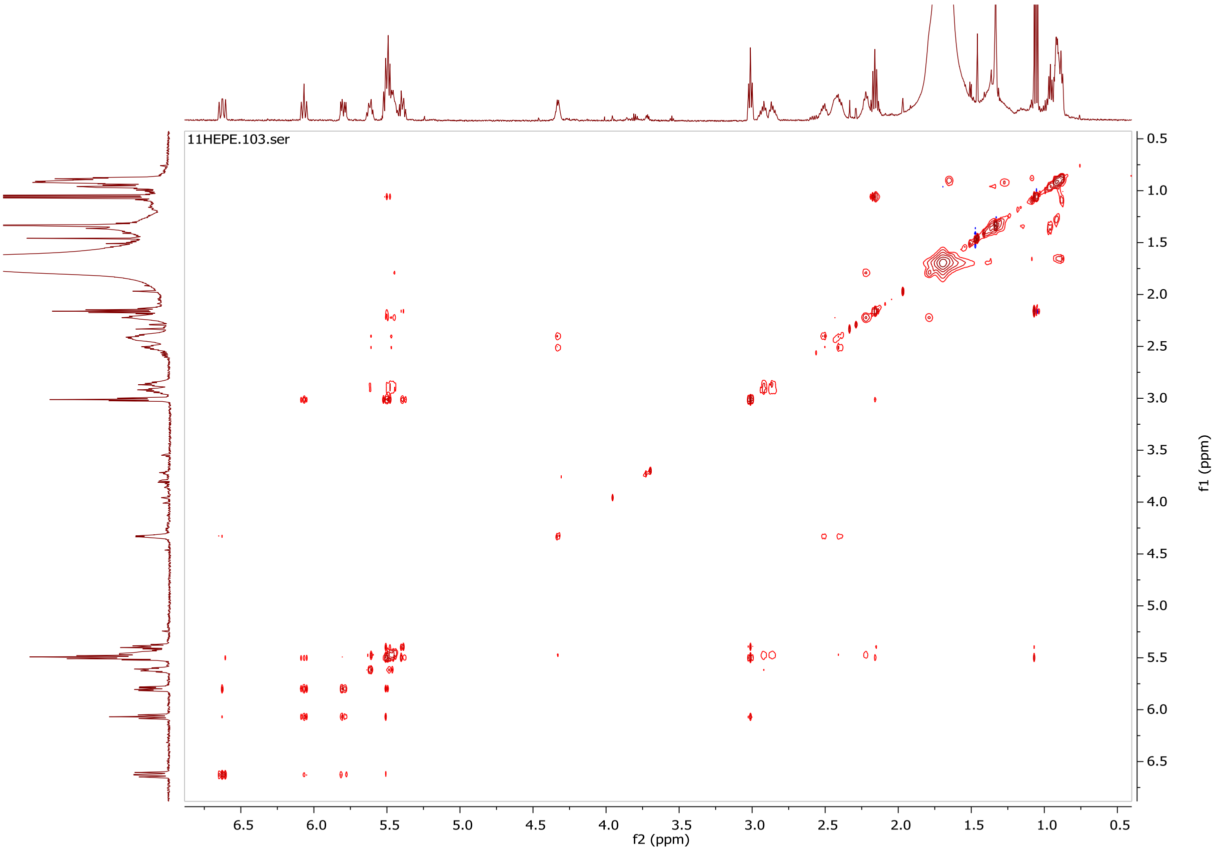

Supplemental Figure S18 – TOCSY NMR spectrum of the reference compound 11-HEPE.

D)

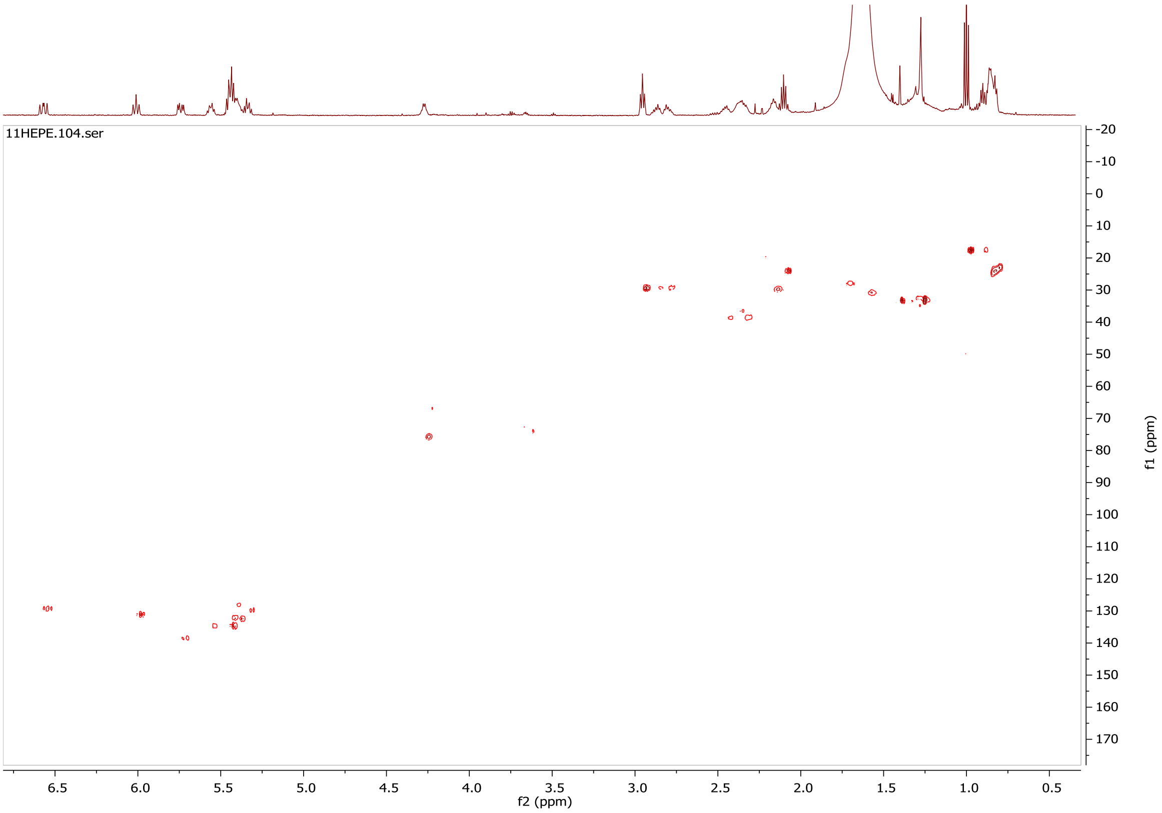

Supplemental Figure S19 – HSQC spectrum of the reference compound 11-HEPE.

13-HDHA

A)

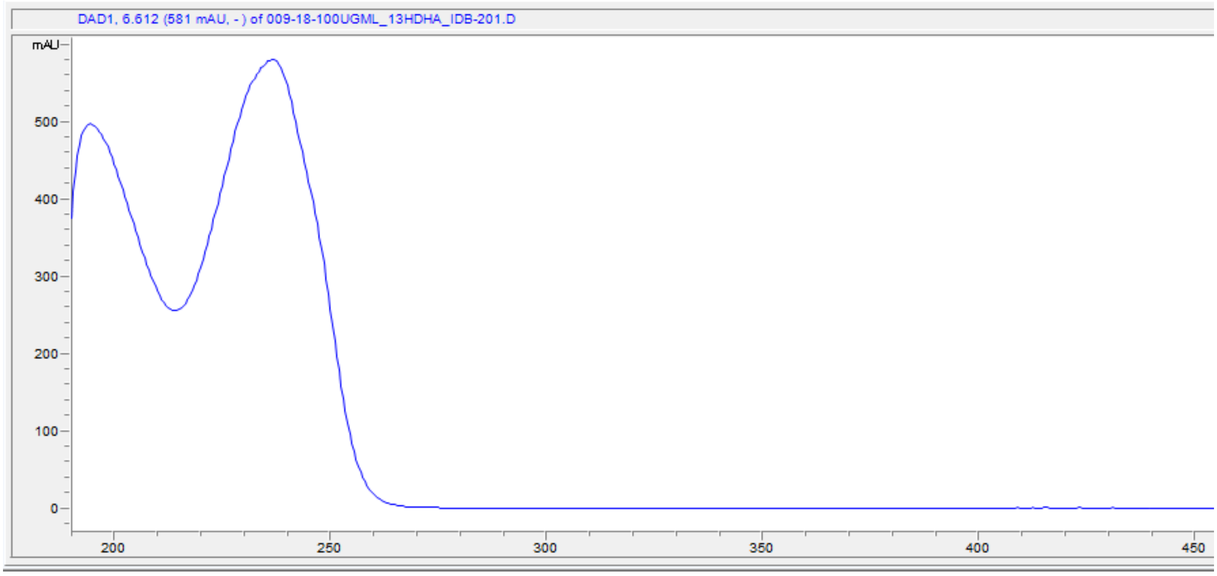

Supplemental Figure S20 – UV-absorption spectrum of 100 µg/mL 13-HDHA.

B)

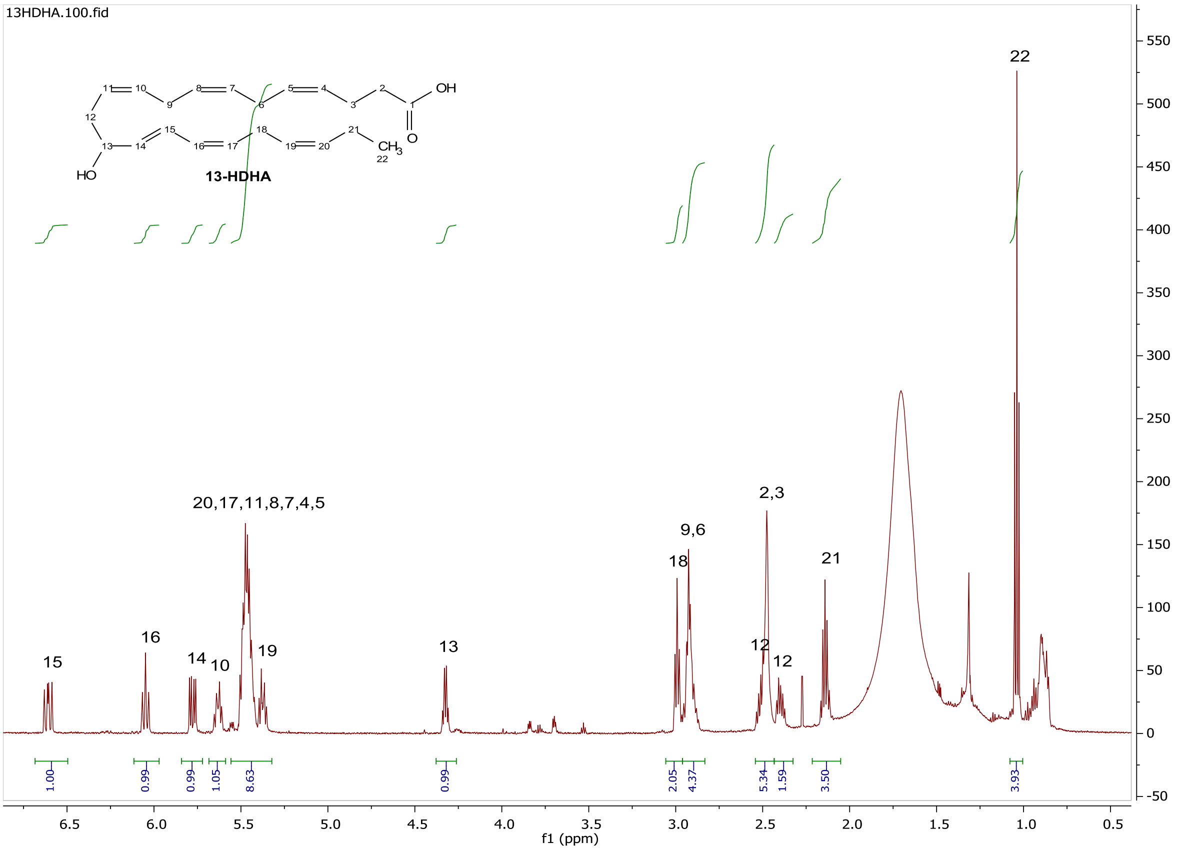

Supplemental Figure S21 – ^{1}H NMR of the reference compound 13-HDHA (600 MHz, chloroform-*d*) δ 6.61 (dd, *J* = 15.2, 11.0 Hz, 1H), 6.05 (dd, *J* = 11.8, 10.0 Hz, 1H), 5.78 (dd, *J* = 15.2, 6.6 Hz, 1H), 5.63 (dt, *J* = 9.2, 7.3 Hz, 1H), 5.53–5.31 (m, 8H), 4.32 (q, *J* = 6.5 Hz, 1H), 2.99 (t, *J* = 7.5 Hz, 2H), 2.95–2.86 (m, 4H), 2.58–2.43 (m, 5H), 2.40 (dt, *J* = 13.9, 6.5 Hz, 1H), 2.21–2.06 (m, 2H), 1.04 (t, *J* = 7.5 Hz, 3H).

C)

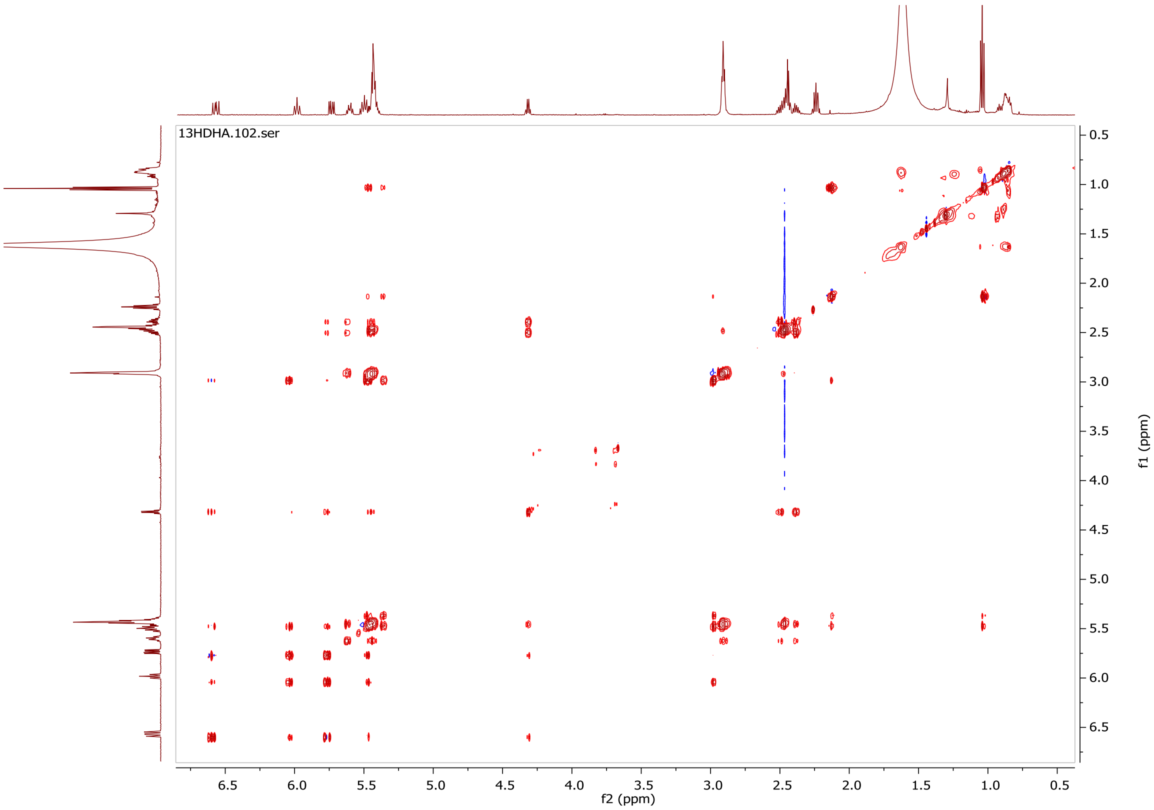

Supplemental Figure S22 – TOCSY NMR spectrum of the reference compound 13-HDHA.

D)

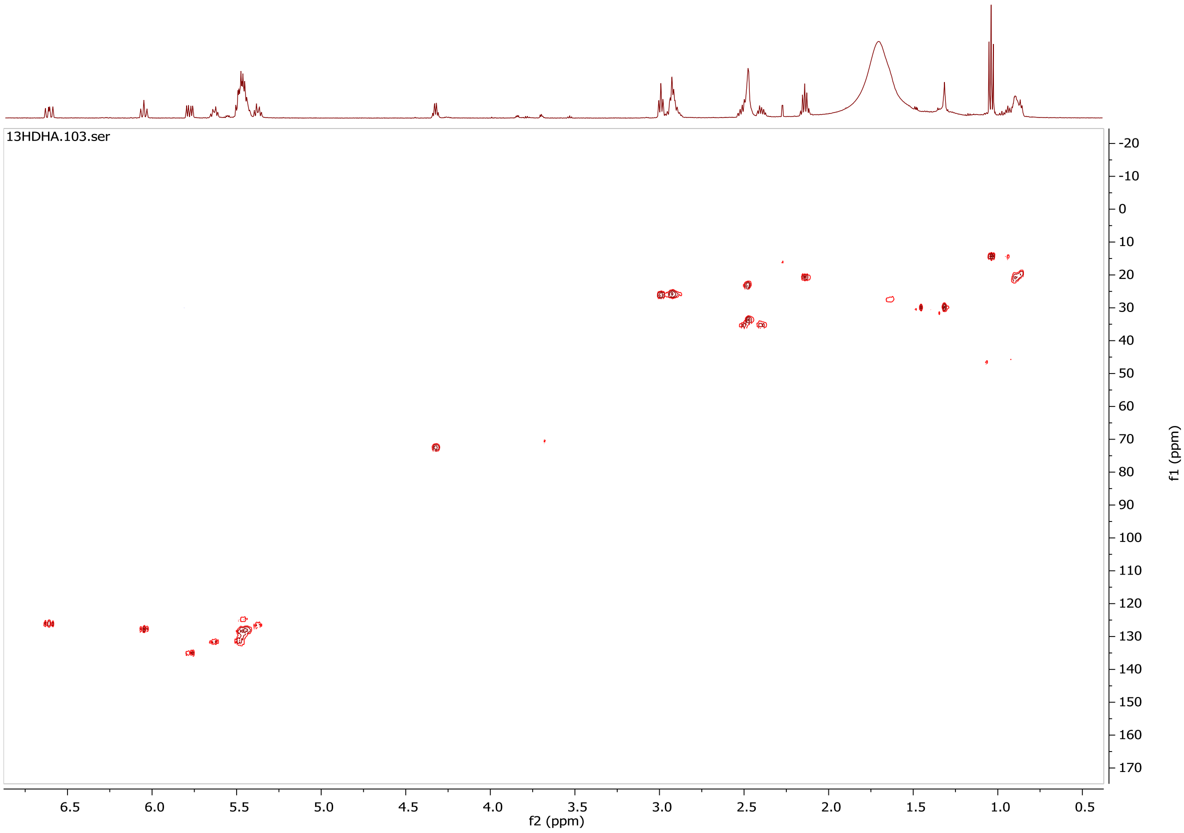

Supplemental Figure S23 – HSQC spectrum of the reference compound 13-HDHA.

16-HDHA

A)

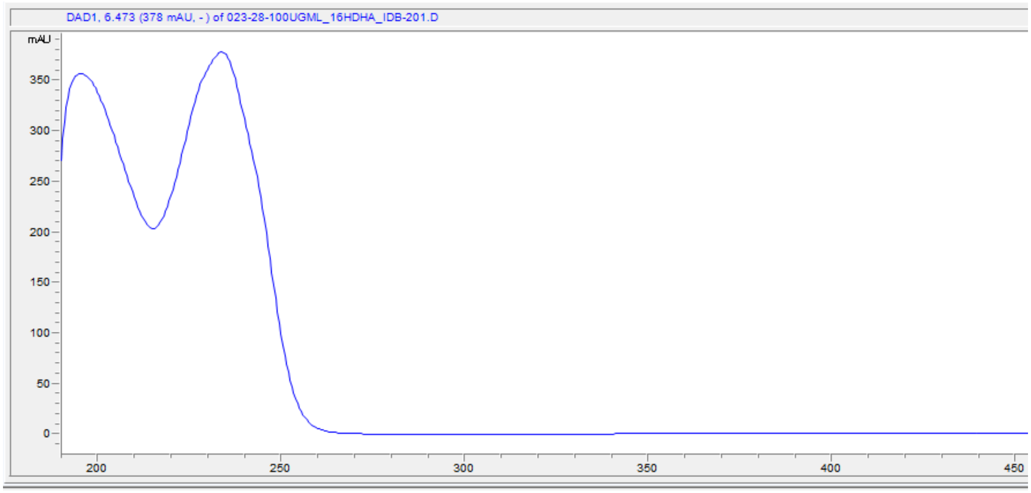

Supplemental Figure S24 – UV-absorption spectrum of 100 µg/mL 16-HDHA.

B)

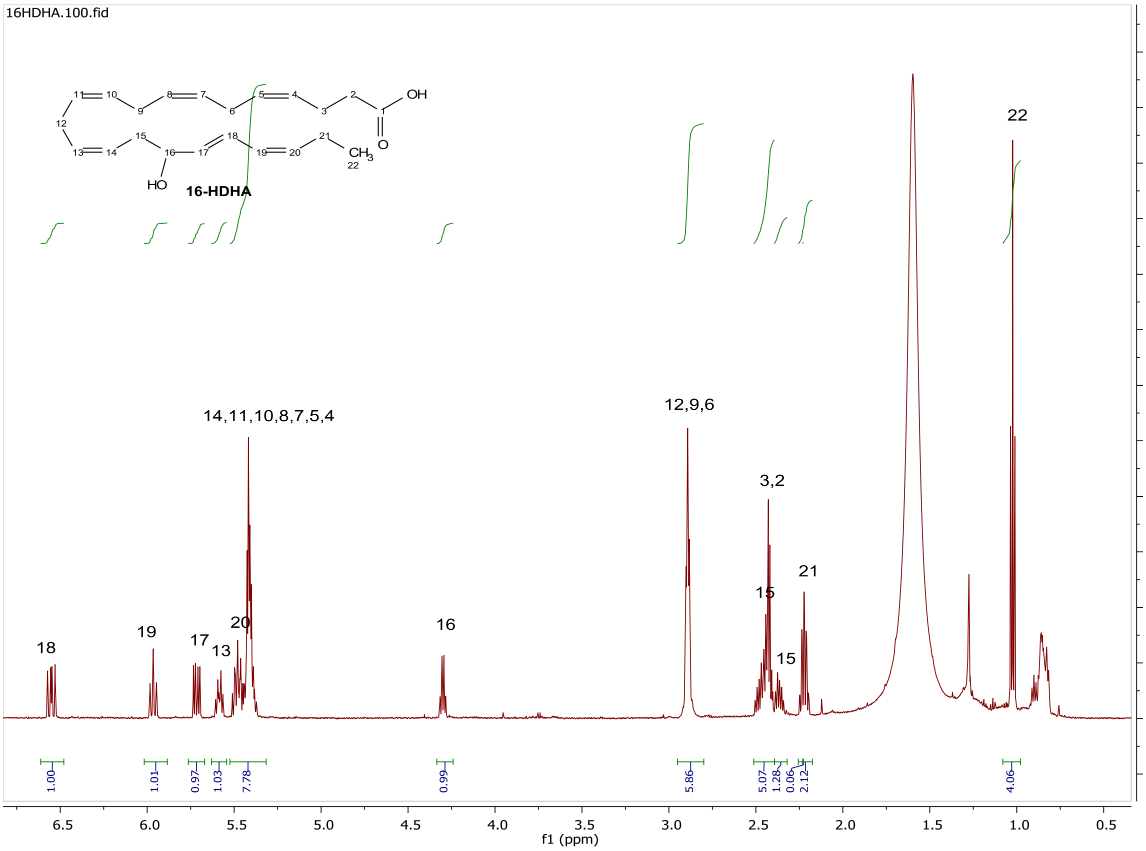

Supplemental Figure S25 – ^{1}H NMR of the reference compound 16-HDHA (600 MHz, chloroform-*d*) δ 6.62–6.50 (m, 1H), 6.04–5.92 (m, 1H), 5.73 (dd, *J* = 15.2, 6.7 Hz, 1H), 5.64–5.55 (m, 1H), 5.54–5.36 (m, 8H), 4.32 (q, *J* = 6.5 Hz, 1H), 2.91 (t, *J* = 6.0 Hz, 6H), 2.56–2.41 (m, 5H), 2.38 (dt, *J* = 13.7, 6.6 Hz, 1H), 2.24 (pd, *J* = 7.6, 1.6 Hz, 2H), 1.04 (t, *J* = 7.5 Hz, 3H).

C)

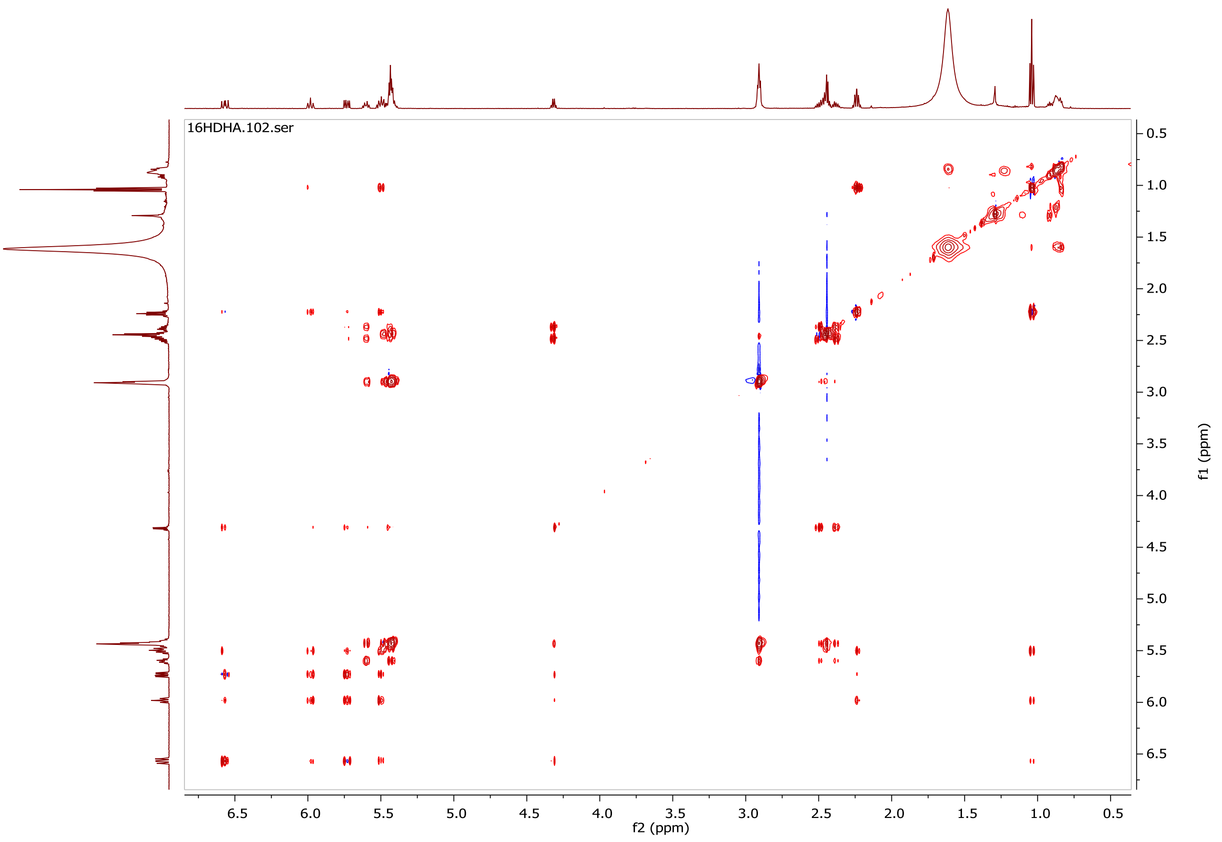

Supplemental Figure S26 – TOCSY spectrum of the reference compound 16-HDHA.

D)

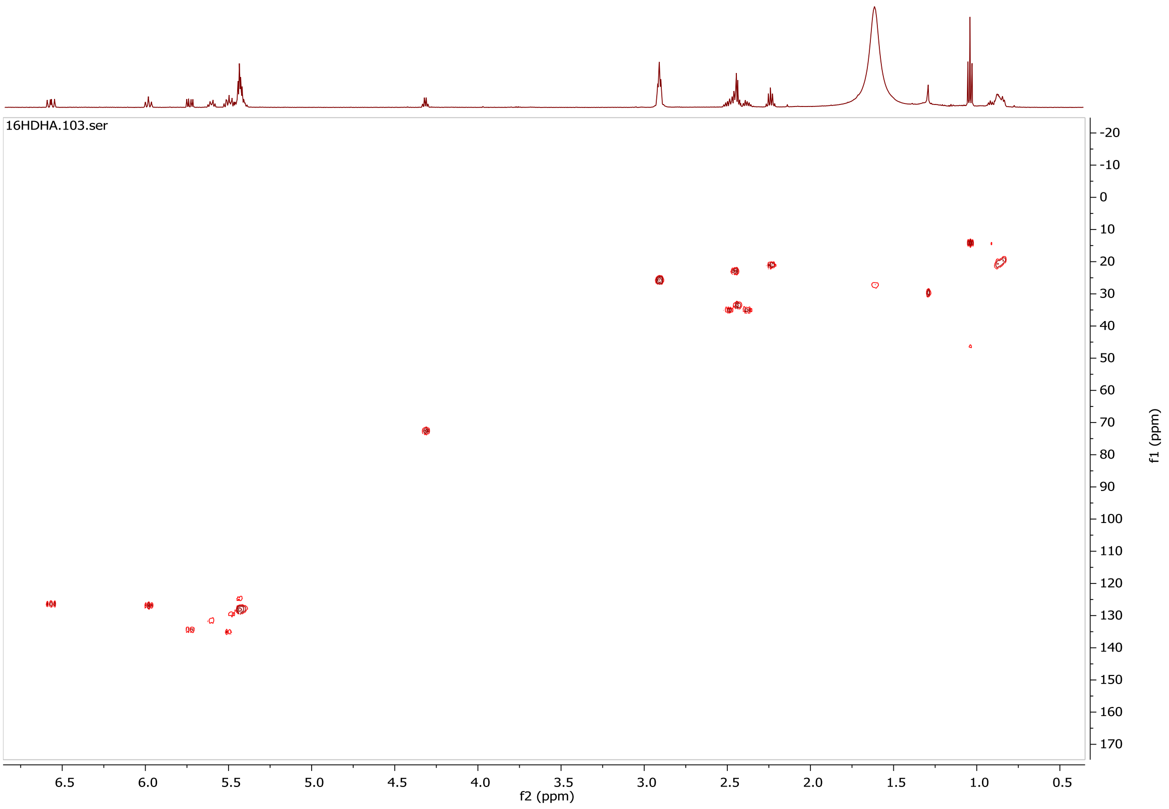

Supplemental Figure S27 – HSQC spectrum of the reference compound 16-HDHA.

PGE$_2$-EA-*d*$_4$

A)

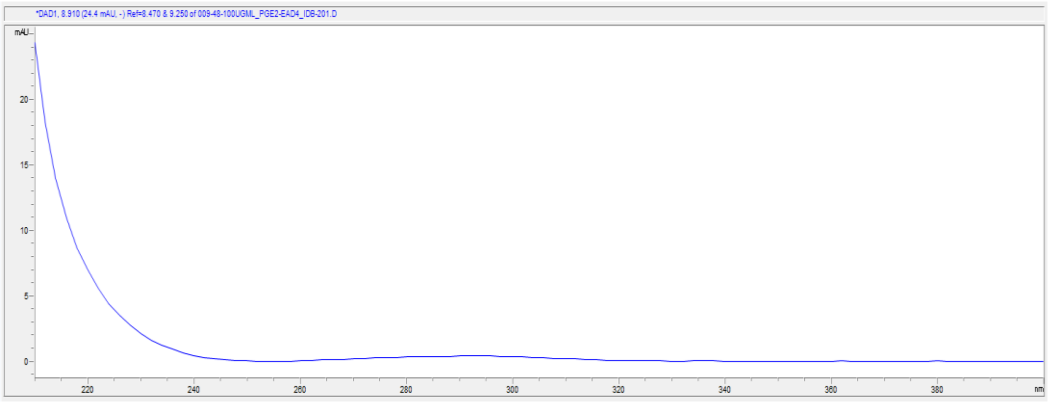

Supplemental Figure S28 – UV-absorption spectrum of 100 µg/mL PGE$_2$-EA-*d*$_4$ used for the quantification of PGE$_3$-EA.

In most ^{1}H NMR spectra of the standards depicted below, which were measured on ver low amounts of products that were obtained after lyophilisation of the preparative HPLC fractions that contained the products, we found unexpected signals in the 0.75–1.65 ppm. Analysis of a lyophilised representative amount of MilliQ/ACN as was obtained by pooling the prep-HPLC fractions indicated that these signals originated from this solvent mixture (Figure S29). Therefore, these signals are found in all synthesized standards.

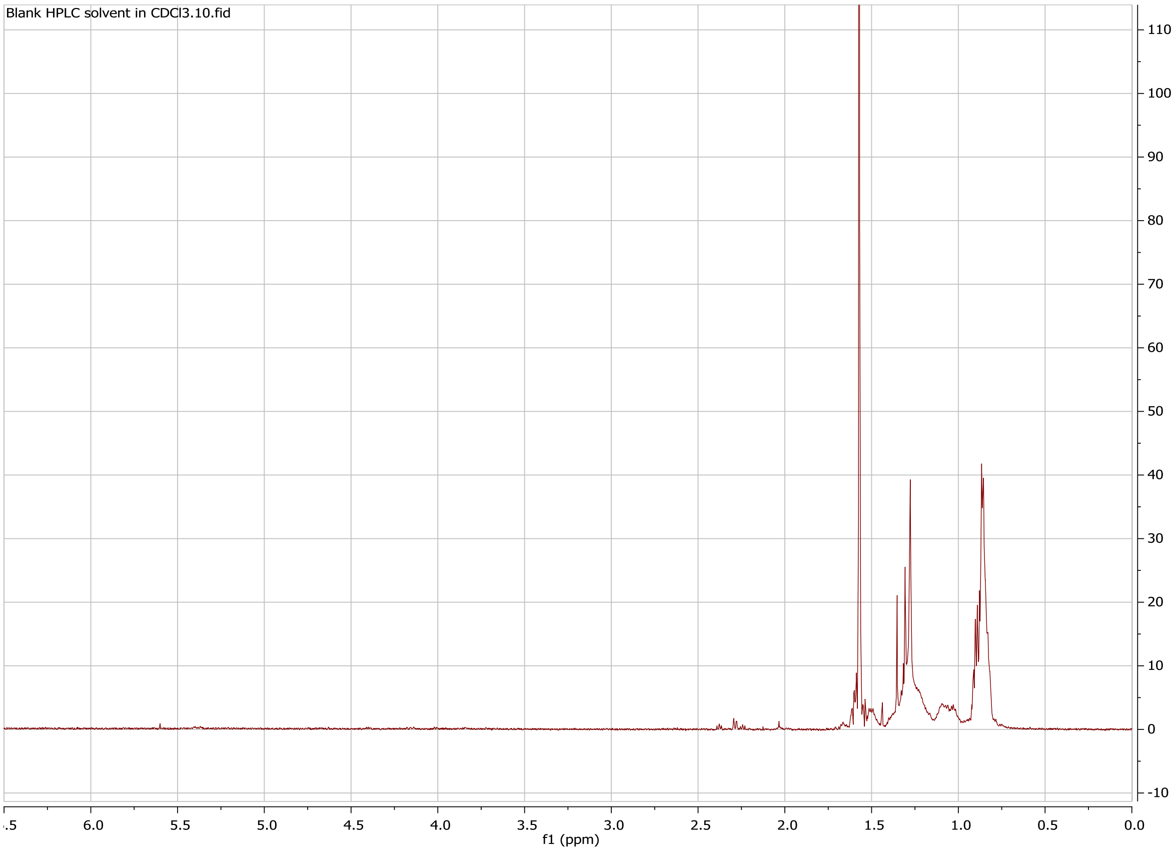

Supplemental Figure S29 – ^{1}H NMR of a concentrated sample of the ACN/Ultrapure water mixture used for the preparative purification of the synthesised standards (600 MHz, chloroform-*d*).

Analysis of synthesised *N*-acylethanolamide controls

A) Chromatograms of the preparative HPLC purifications.
B) Mass spectrum (positive mode) of the isolated product as indicated with a red circle in chromatogram A.
C) Absorption spectrum of the isolated product.
D) ^{1}H-NMR spectrum of the isolated product.
E) TOCSY-NMR spectrum of the isolated product.
F) HSQC-NMR spectrum of the isolated product.

PGE$_3$-EA

A)

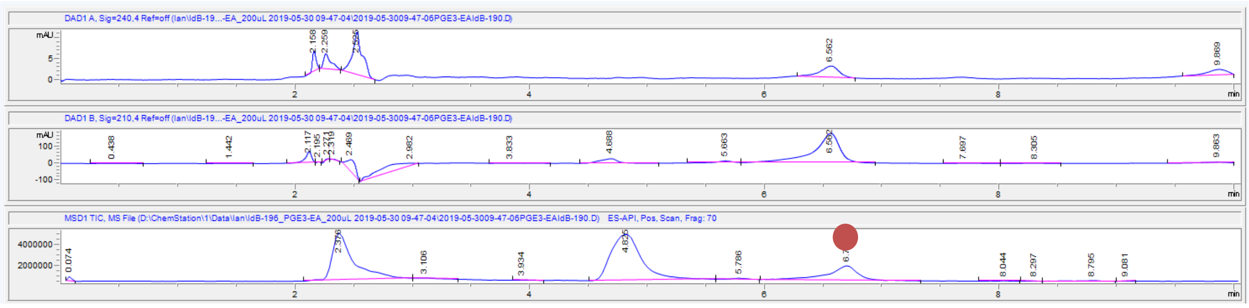

Supplemental Figure S30 – Chromatograms of the preparative HPLC purification of PGE$_3$-EA (top trace: absorption at 240 nm, middle trace: absorption at 210 nm, bottom trace: TIC signal of the positive ionisation channel) The red circle represents the product peak.

B)

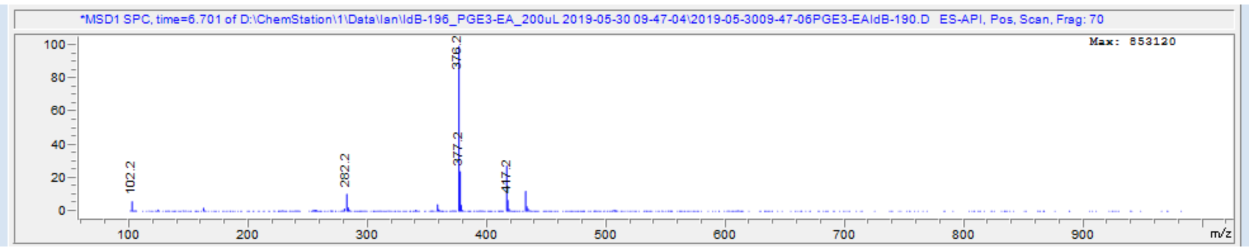

Supplemental Figure S31 – Mass spectrum of the isolated product peak of PGE$_3$-EA (positive mode). The mass spectrum displayed is from the isolated product peak as indicated by the red circle in figure A.

C)

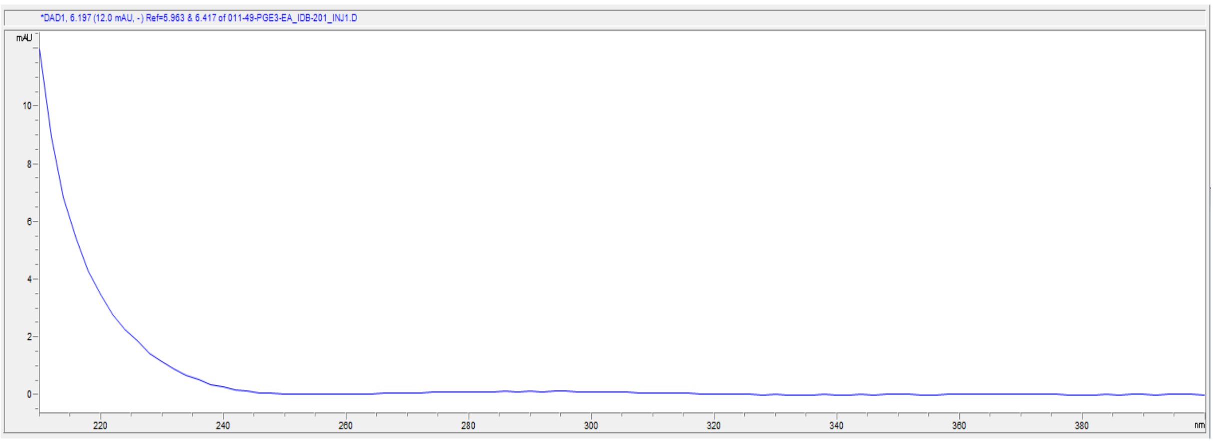

Supplemental Figure S32 – UV-absorption spectrum of the purified PGE_3-EA.

D)

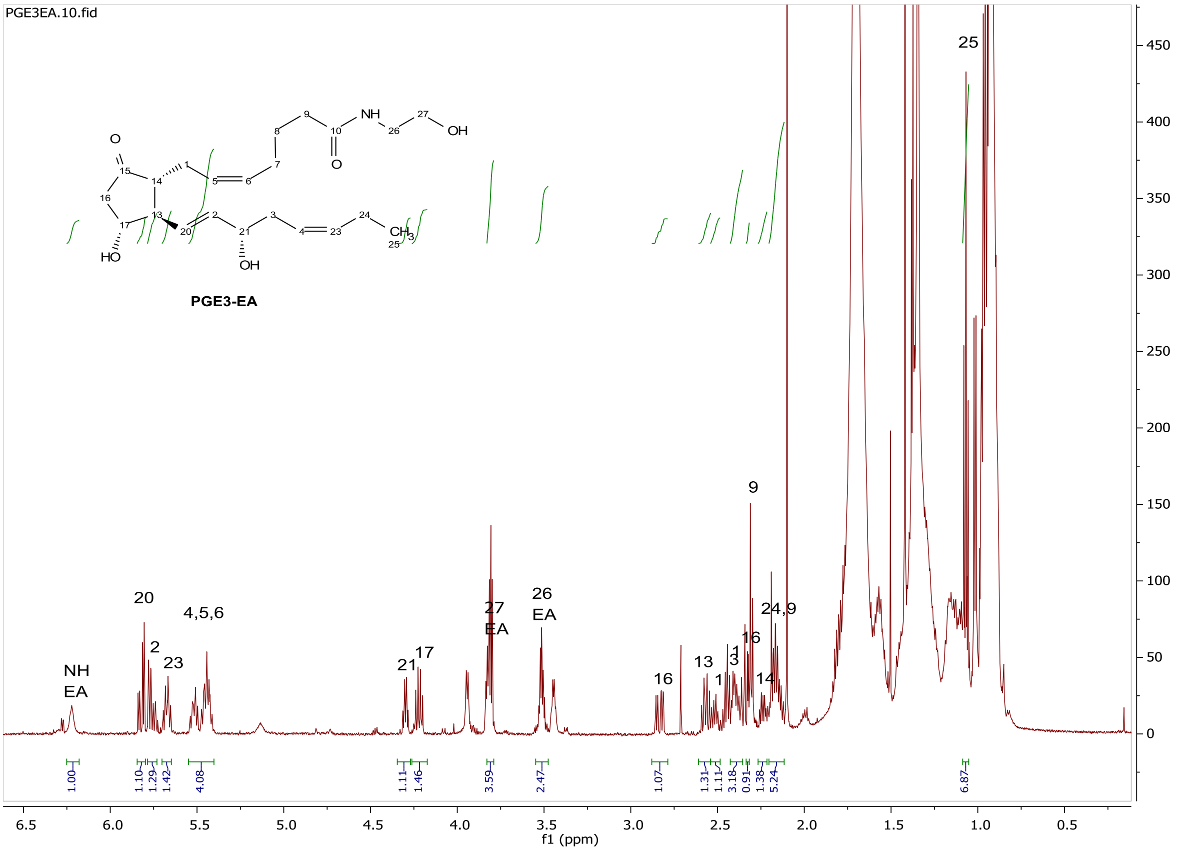

Supplemental Figure S33 – ^{1}H NMR of the PGE_3-EA standard (600 MHz, chloroform-*d*) δ 6.22 (s, 1H), 5.82 (dd, *J* = 15.5, 5.4 Hz, 1H), 5.77 (dd, *J* = 8.3, 7.3 Hz, 1H), 5.67 (dddd, *J* = 10.7, 8.8, 5.2, 1.6 Hz, 1H), 5.56–5.39 (m, 3H), 4.30 (dt, *J* = 7.6, 5.6 Hz, 1H), 4.25–4.19 (m, 1H), 3.82–3.79 (m, 2H), 3.51 (qd, *J* = 5.2, 3.6 Hz, 2H), 2.83 (ddd, *J* = 18.5, 7.3, 1.4 Hz, 1H), 2.58 (dd, *J* = 11.2, 7.9 Hz, 1H), 2.54–2.49 (m, 1H), 2.43–2.37 (m, 2H), 2.33–2.32 (m, 1H), 2.32–2.28 (m, 2H), 2.26–2.21 (m, 1H), 2.20–2.11 (m, 4H), 1.07 (t, *J* = 7.5 Hz, 3H).

Peak assignment was performed by using 2D TOCSY spectrum and the reference spectra of PGE_2 and PGE_3 as described in the literature (2, 3).

E)

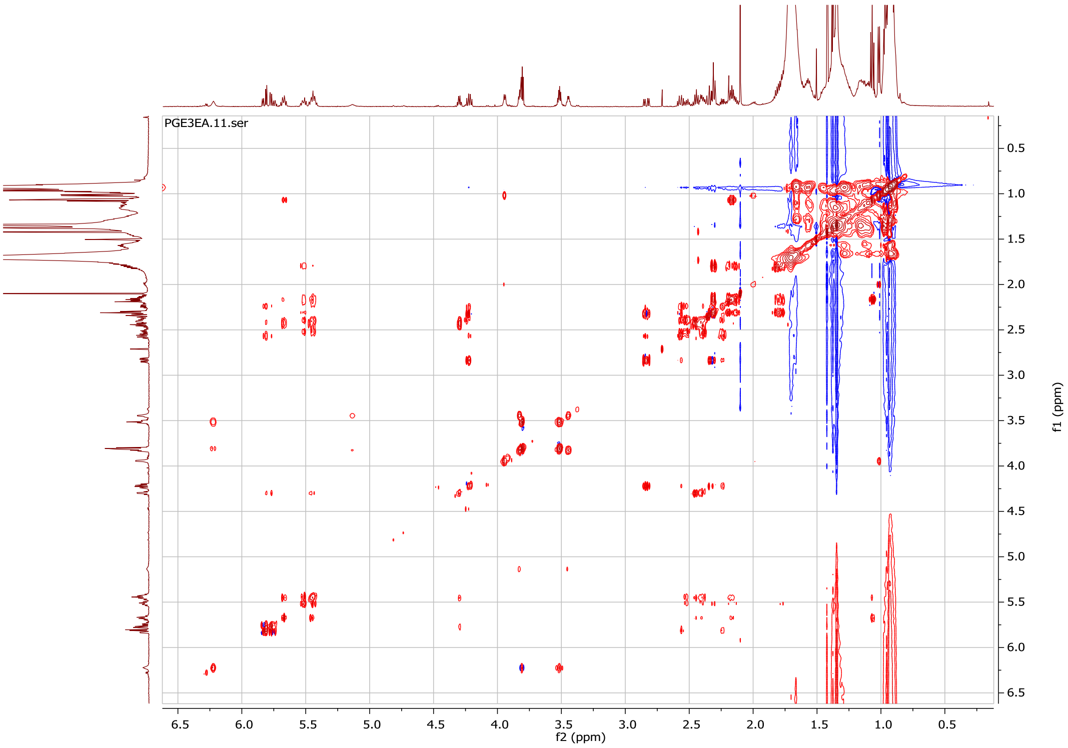

Supplemental Figure S34 – TOCSY NMR spectrum of the PGE$_3$-EA standard.

F)

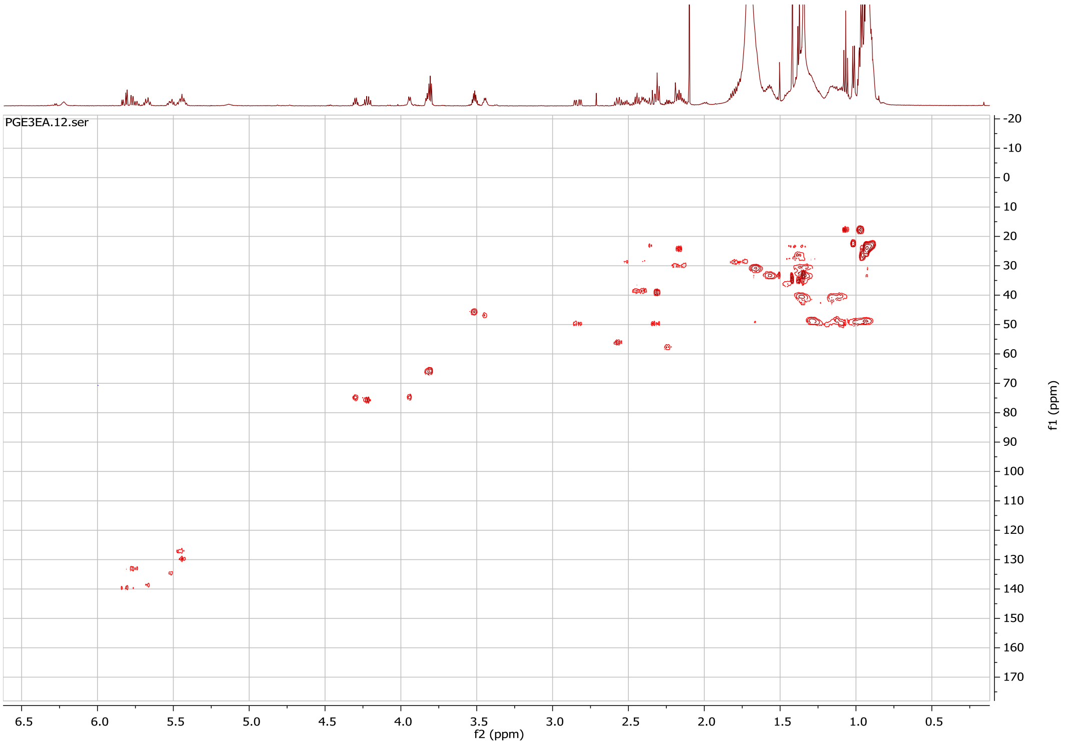

Supplemental Figure S35 – HSQC NMR spectrum of the PGE$_3$-EA standard.

11-HEPE-EA

A)

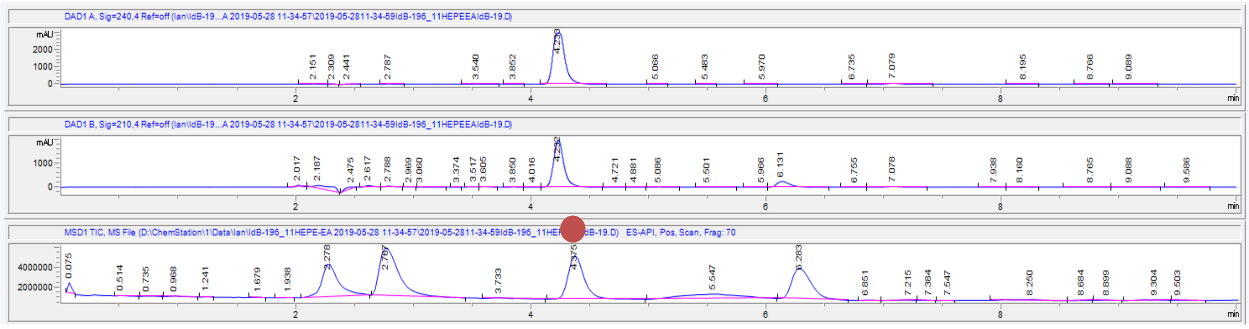

Supplemental Figure S36 – Chromatograms of the preparative HPLC purification of 11-HEPE-EA. (top trace: absorption at 240 nm, middle trace: absorption at 210 nm, bottom trace: TIC signal of the positive ionisation channel) The red circle represents the product peak.

B)

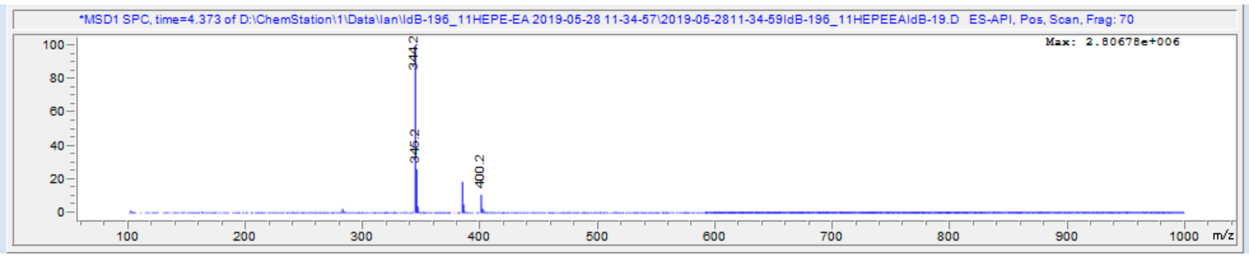

Supplemental Figure S37 – Mass spectrum of the isolated product peak of 11-HEPE-EA (positive mode). The mass spectrum displayed is from the isolated product peak as indicated by the red circle in figure A.

C)

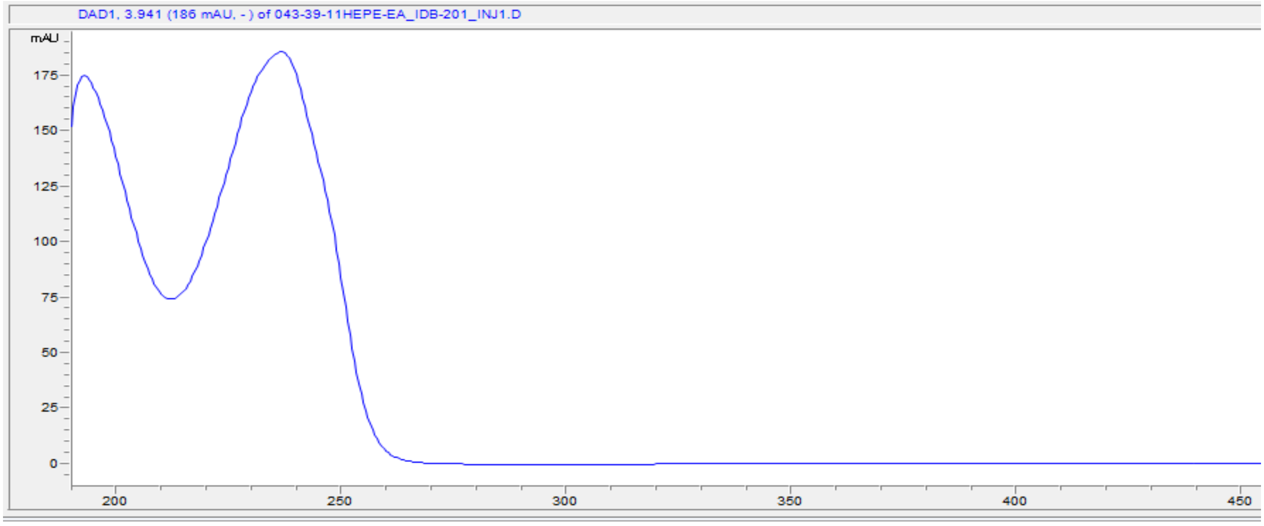

Supplemental Figure S38 – UV-absorption spectrum of the purified 11-HEPE-EA.

D)

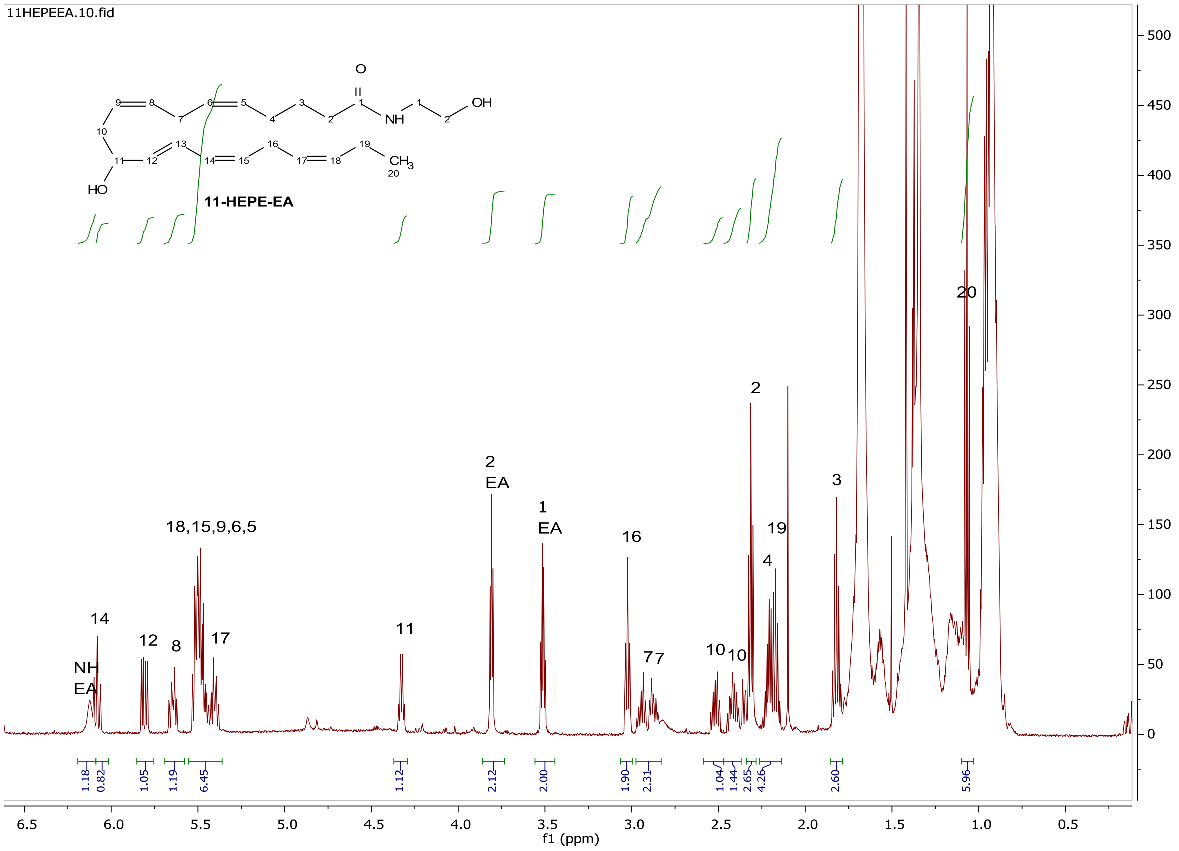

Supplemental Figure S39 – ^{1}H NMR of 11-HEPE-EA standard (600 MHz, chloroform-*d*) δ 6.12 (s, 1H), 6.11–6.04 (m, 1H), 5.81 (dd, *J* = 15.2, 6.4 Hz, 1H), 5.68–5.60 (m, 1H), 5.55–5.36 (m, 6H), 4.33 (q, *J* = 6.4 Hz, 1H), 3.81 (t, *J* = 5.0 Hz, 2H), 3.51 (td, *J* = 5.7, 4.1 Hz, 2H), 3.02 (t, *J* = 7.5 Hz, 2H), 2.98–2.84 (m, 2H), 2.56–2.47 (m, 1H), 2.46–2.38 (m, 1H), 2.31 (t, *J* = 7.3 Hz, 2H), 2.24–2.14 (m, 4H), 1.82 (p, *J* = 7.3 Hz, 2H), 1.07 (t, *J* = 7.5 Hz, 3H).

E)

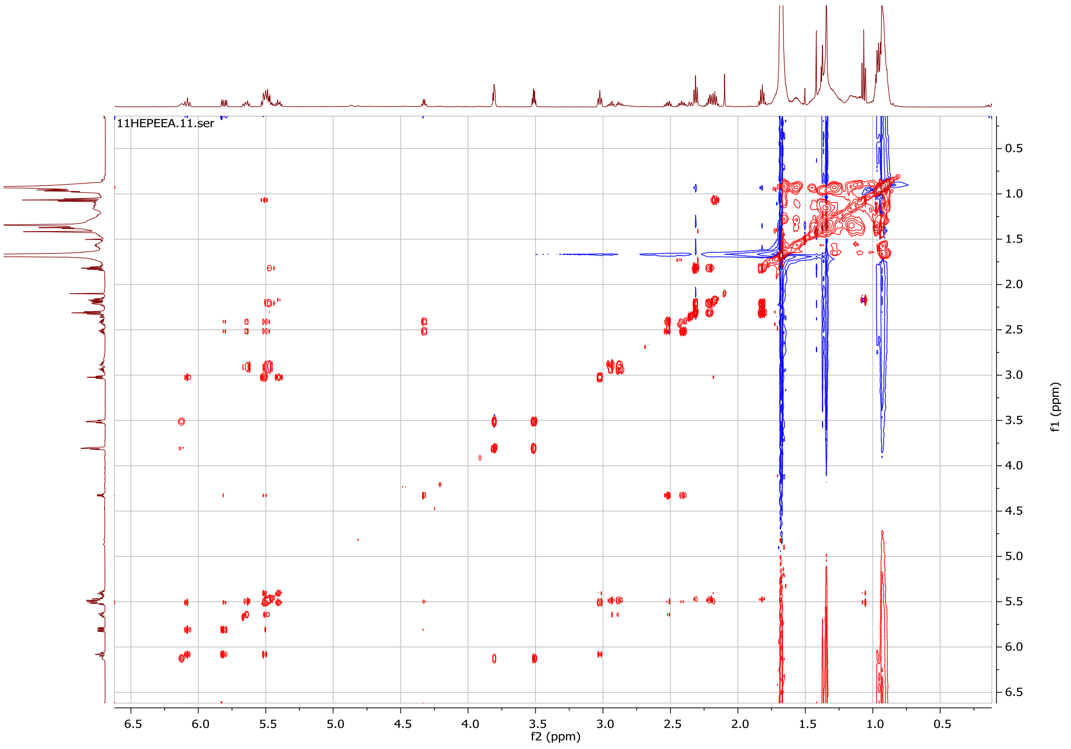

Supplemental Figure S40 – TOCSY NMR spectrum of the 11-HEPE-EA standard.

F)

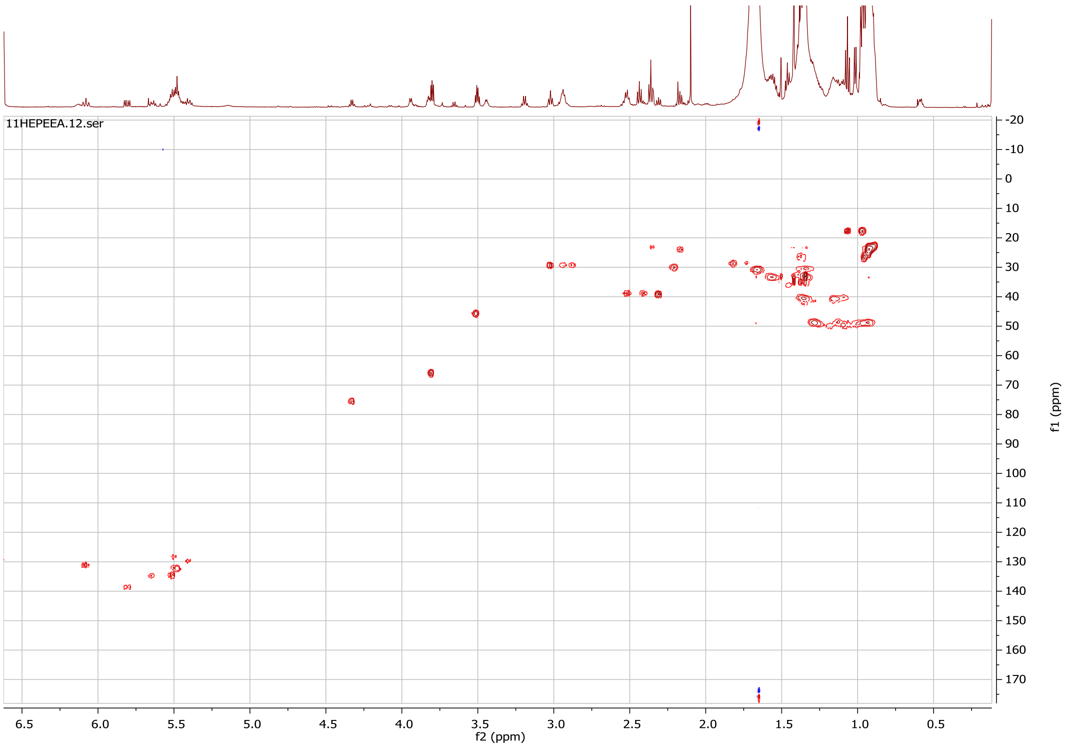

Supplemental Figure S41 – HSQC NMR of the 11-HEPE-EA standard.

11-HEPE-EA-d_4

A)

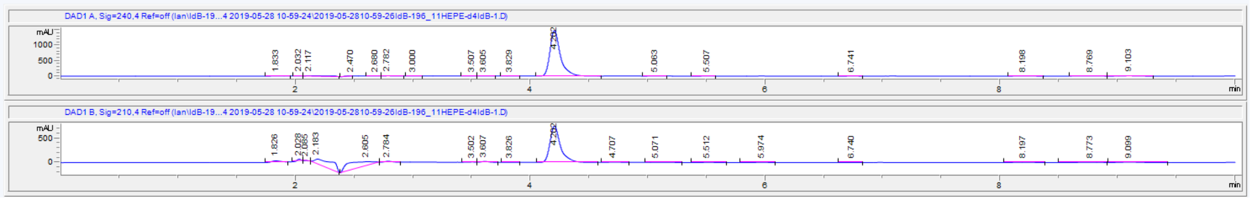

Supplemental Figure S42 – Chromatograms of the preparative HPLC purification of 11-HEPE-EA-d_4 (top trace: absorption at 240 nm, middle trace: absorption at 210 nm).

B)

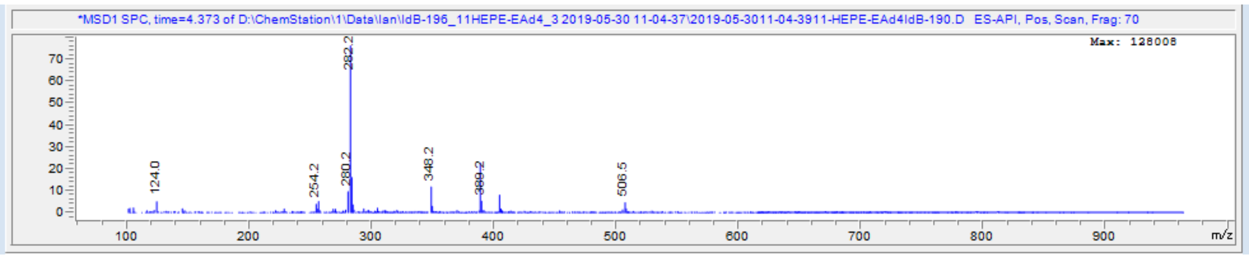

Supplemental Figure S43 – Mass spectrum of the product peak of 11-HEPE-EA-d4 (positive mode).

C)

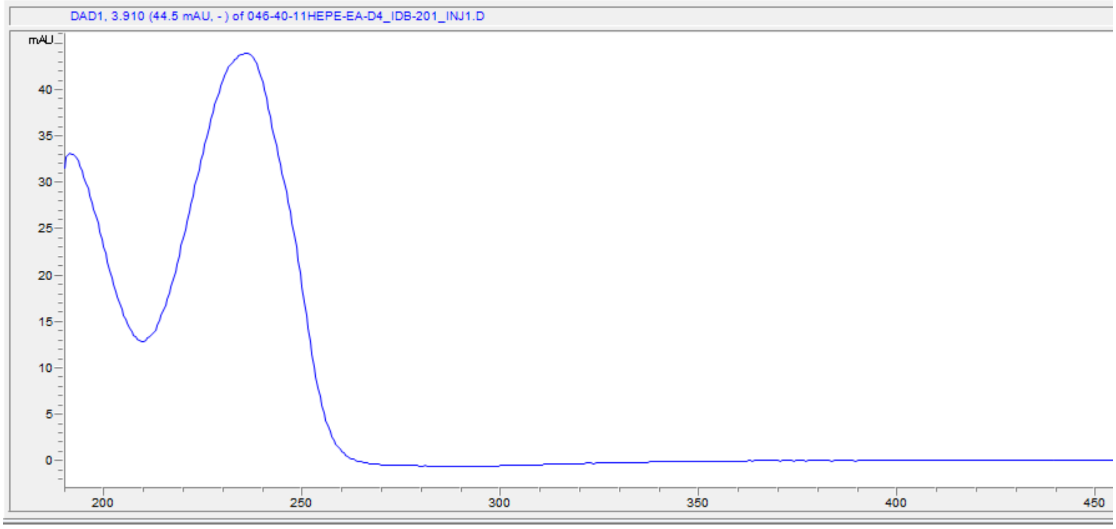

Supplemental Figure S44 – UV-absorption spectrum of the purified 11-HEPE-EA-d_4.

D)

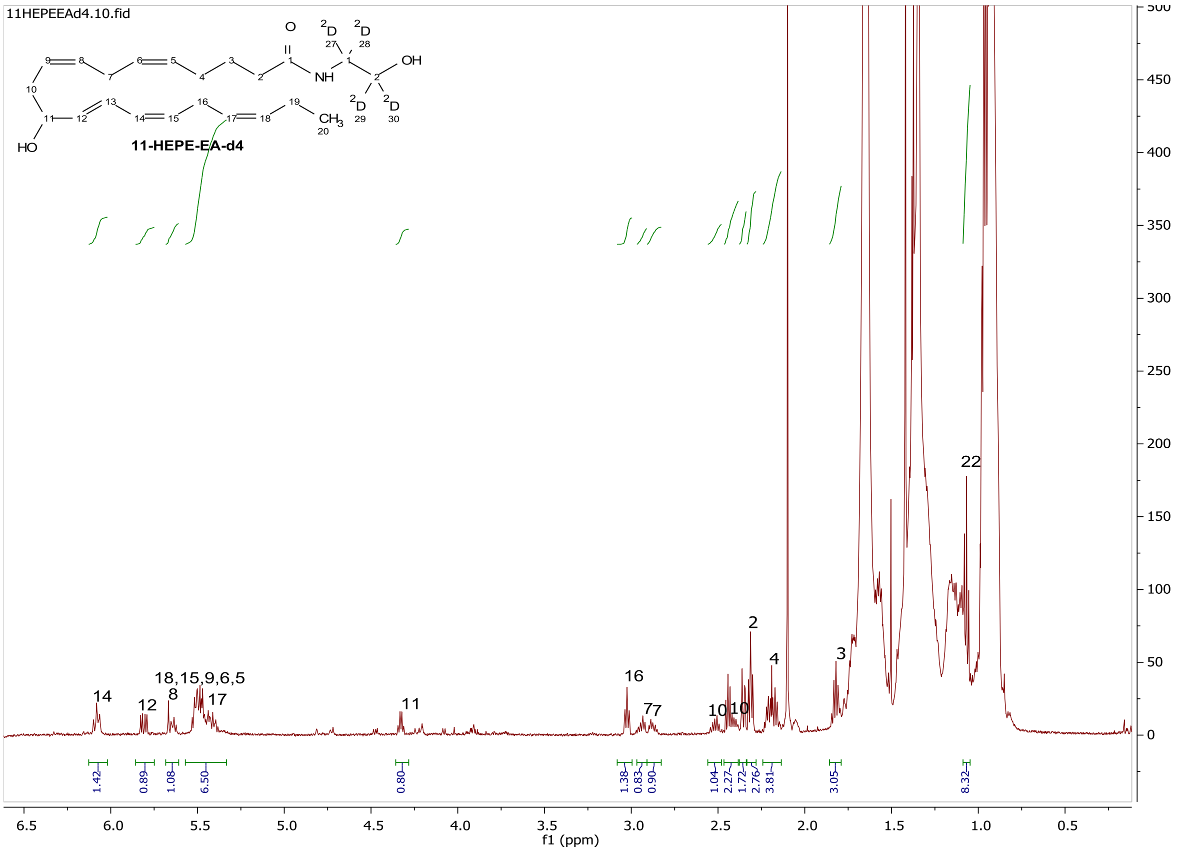

Supplemental Figure S45 – ^{1}H NMR of the 11-HEPE-EA-d_4 standard (600 MHz, chloroform-d) δ 6.08 (t, J = 10.6 Hz, 1H), 5.81 (dd, J = 15.2, 6.4 Hz, 1H), 5.71–5.60 (m, 1H), 5.57–5.34 (m, 6H), 4.33 (q, J = 6.6 Hz, 1H), 3.02 (t, J = 7.5 Hz, 2H), 2.91 (ddd, J = 35.9, 15.6, 8.1 Hz, 2H), 2.52 (dt, J = 15.0, 7.5 Hz, 1H), 2.42 (dq, J = 20.4, 6.8, 6.1 Hz, 1H), 2.31 (td, J = 7.4, 1.8 Hz, 2H), 2.27–2.13 (m, 2H), 1.82 (p, J = 7.1 Hz, 1H), 1.07 (t, J = 7.5 Hz, 3H).

E)

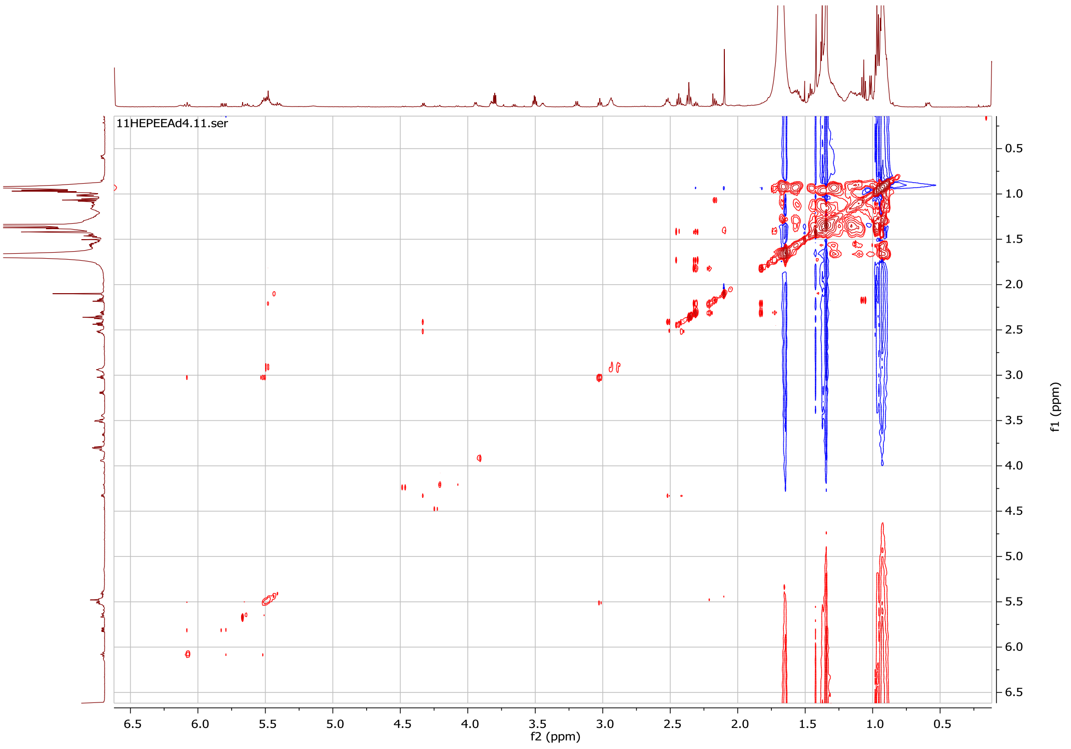

Supplemental Figure S46 – TOCSY NMR spectrum of the 11-HEPE-EA-*d*$_4$ standard.

F)

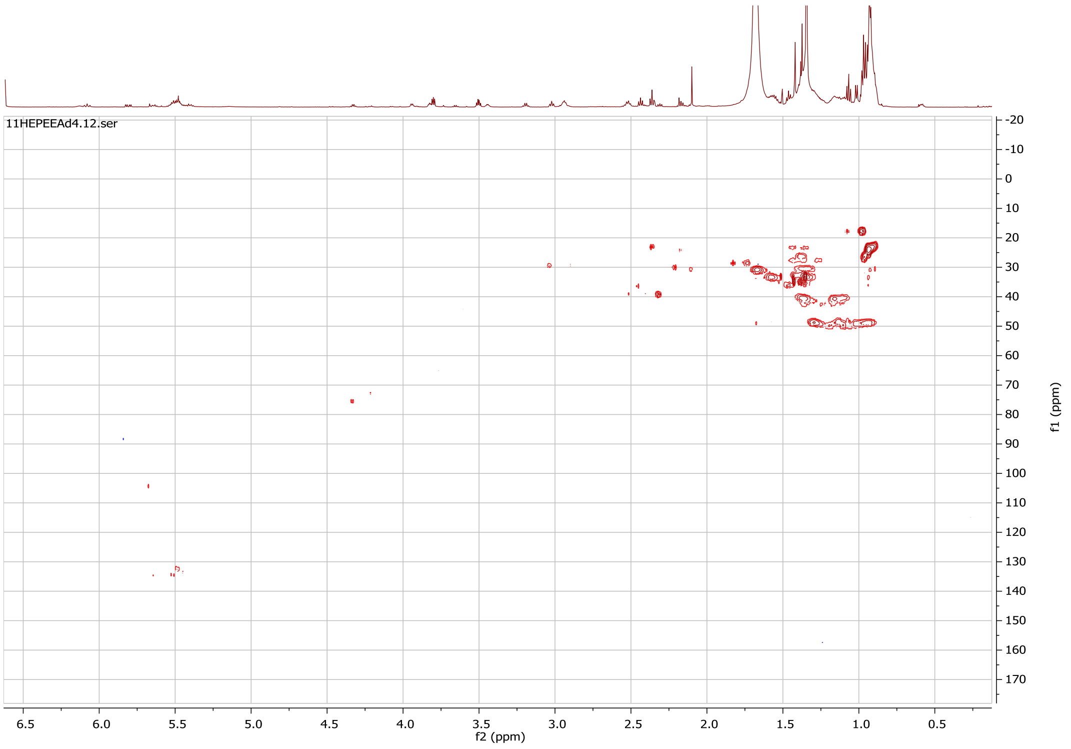

Supplemental Figure S47 – HSQC NMR spectrum of the 11-HEPE-EA-*d*$_4$ standard.

13-HDHEA

A)

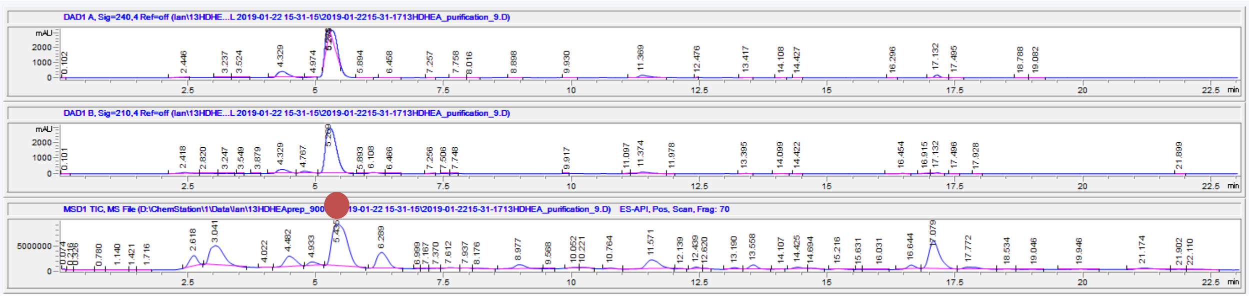

Supplemental Figure S48 – Chromatograms of the preparative HPLC purification of 13-HDHEA (top trace: absorption at 240 nm, middle trace: absorption at 210 nm, bottom trace: TIC signal of the positive ionisation channel). The red circle represents the product peak.

B)

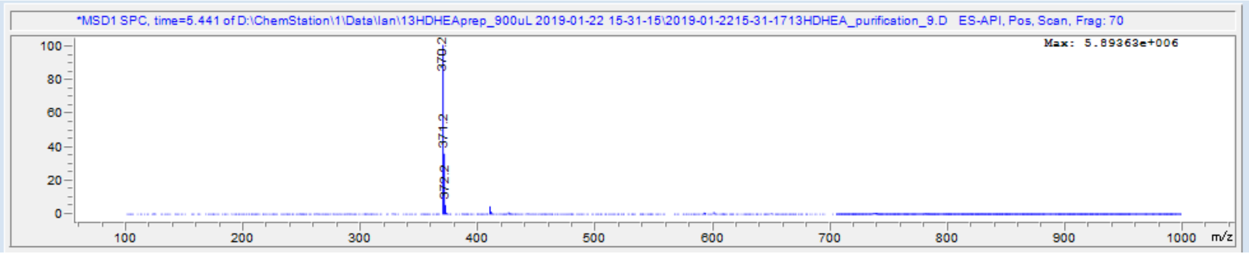

Supplemental Figure S49 – Mass spectrum of the isolated product peak of 13-HDHEA (positive mode). The mass spectrum displayed is from the isolated product peak as indicated by the red circle in figure A.

C)

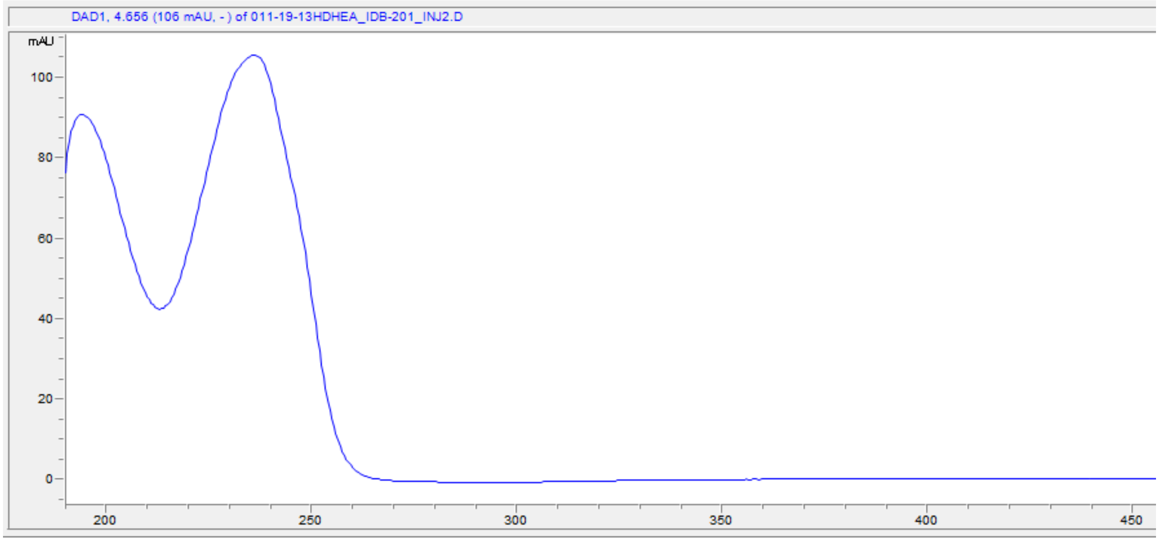

Supplemental Figure S50 – UV-absorption spectrum of the purified 13-HDHEA.

D)

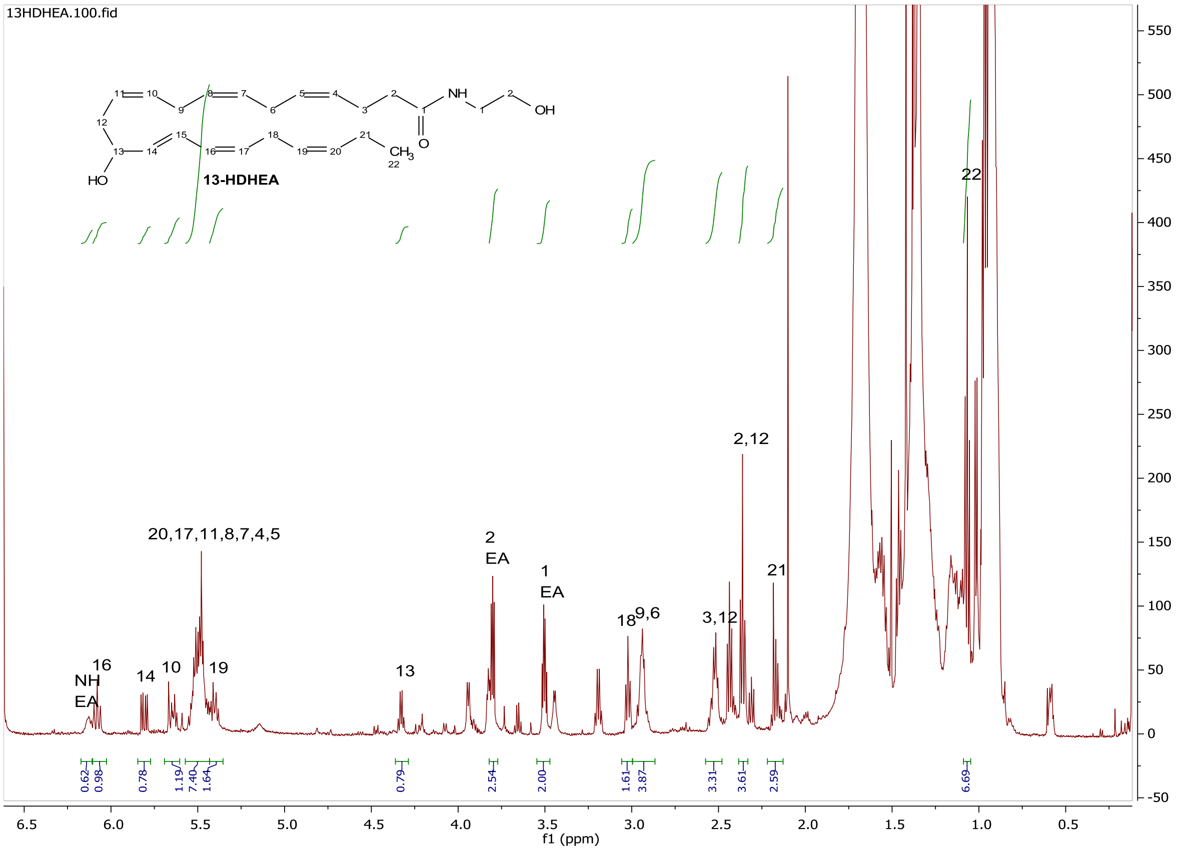

Supplemental Figure S51 – ^{1}H NMR of the standard 13-HDHEA (600 MHz, chloroform-*d*) δ 6.13 (s, 1H), 6.08 (t, *J* = 12.0, 10.1 Hz, 1H), 5.81 (dd, *J* = 15.2, 6.5 Hz, 1H), 5.69–5.61 (m, 1H), 5.56–5.37 (m, 8H), 4.33 (q, *J* = 6.5 Hz, 1H), 3.81 (dt, *J* = 14.8, 5.0 Hz, 2H), 3.50 (q, *J* = 5.3 Hz, 2H), 3.02 (t, *J* = 7.5 Hz, 2H), 2.95 (dt, *J* = 12.3, 6.3 Hz, 4H), 2.58–2.47 (m, 3H), 2.39–2.33 (m, 3H), 2.17 (dq, *J* = 8.1, 6.6 Hz, 3H), 1.07 (t, *J* = 7.5 Hz, 4H).

E)

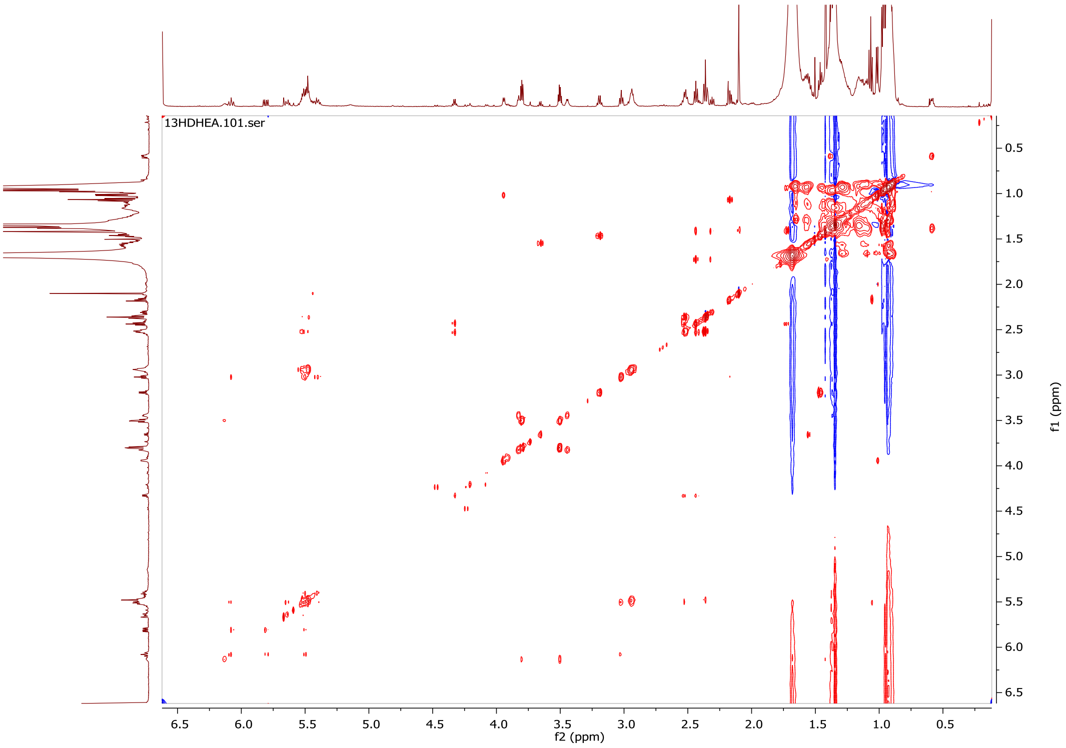

Supplemental Figure S52 – TOCSY NMR spectrum of the 13-HDHEA standard.

F)

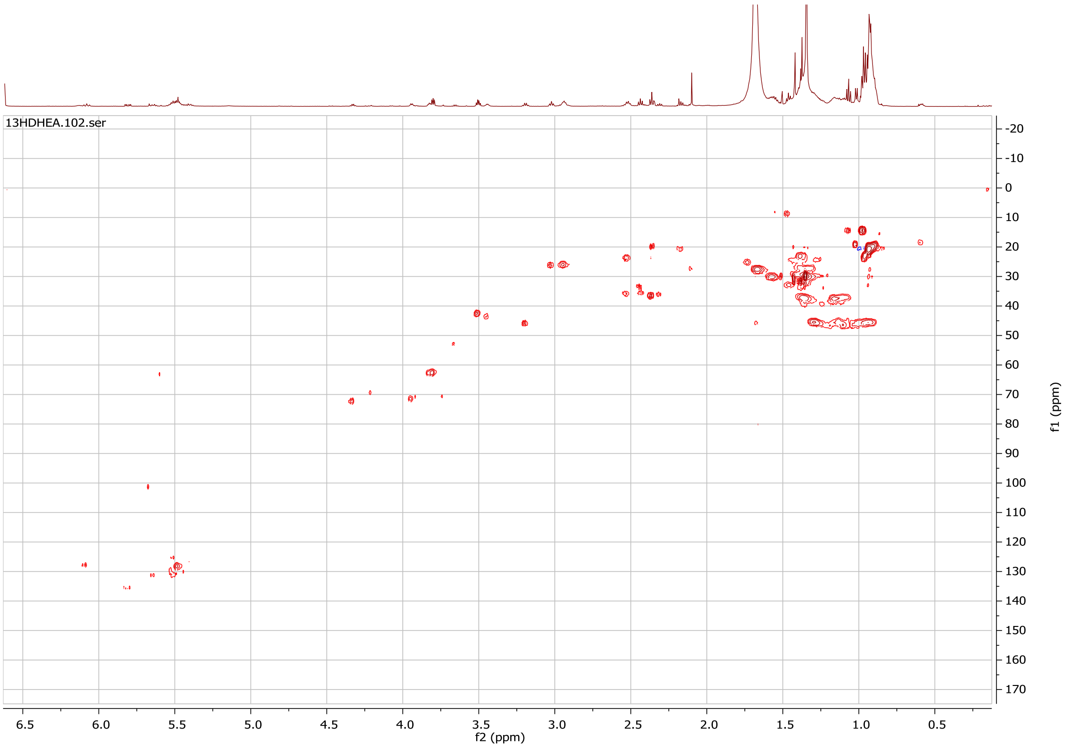

Supplemental Figure S53 – HSQC NMR spectrum of the 13-HDHEA standard.

16-HDHEA

A)

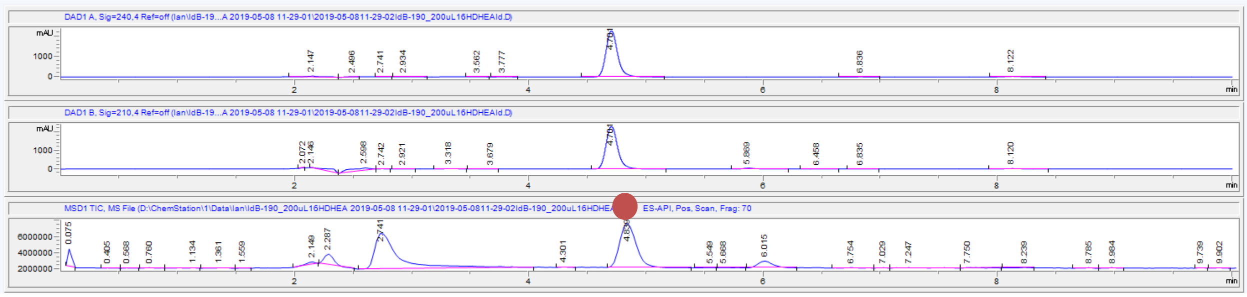

Supplemental Figure S54 – Chromatograms of the preparative HPLC purification of 16-HDHEA (top trace: absorption at 240 nm, middle trace: absorption at 210 nm, bottom trace: TIC signal of the positive ionisation channel). The red circle represents the product peak.

B)

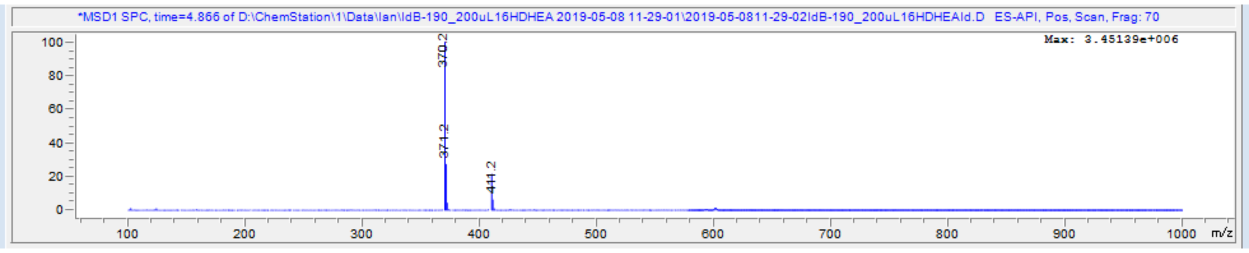

Supplemental Figure S55 – Mass spectrum of the isolated product peak of 16-HDHEA (positive mode). The mass spectrum displayed is from the isolated product peak as indicated by the red circle in figure A.

C)

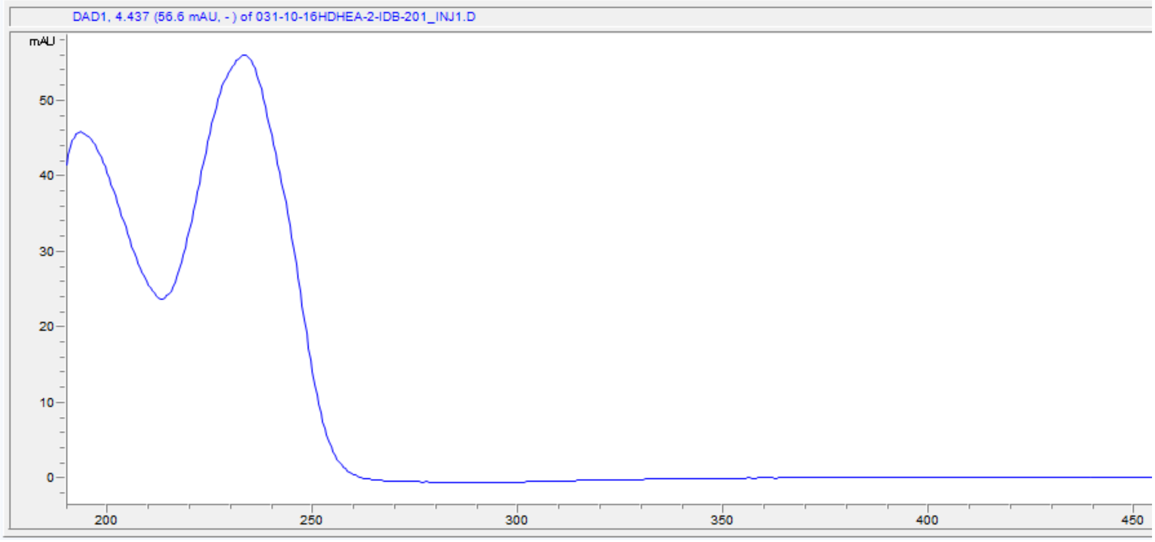

Supplemental Figure S56 – UV-absorption spectrum of the purified 16-HDHEA.

D)

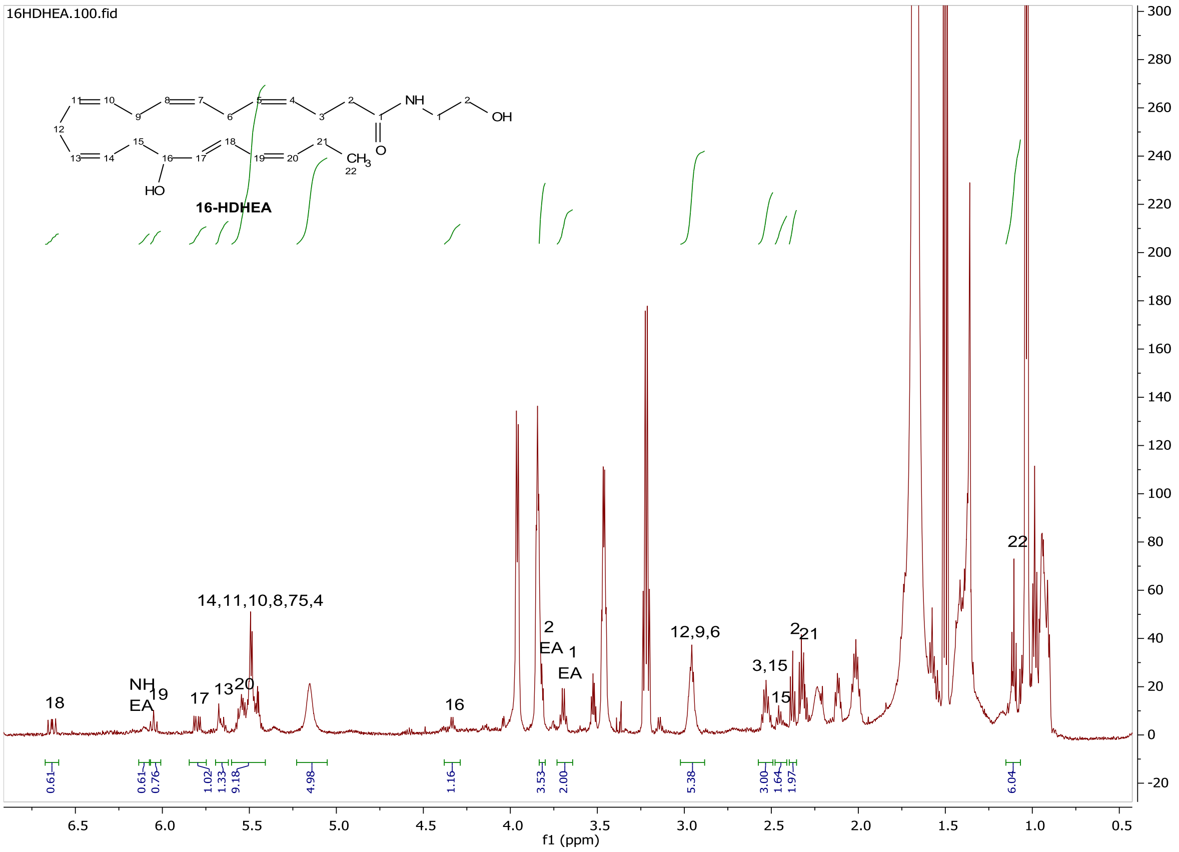

Supplemental Figure S57 – ^{1}H NMR of the 16-HDHEA standard (600 MHz, chloroform-*d*) δ 6.63 (dd, *J* = 15.1, 11.1 Hz, 1H), 6.10 (s, 1H), 6.05 (t, *J* = 11.0 Hz, 1H), 5.80 (dd, *J* = 15.3, 6.5 Hz, 1H), 5.71–5.62 (m, 1H), 5.57–5.41 (m, 8H), 4.34 (d, *J* = 6.5 Hz, 1H), 3.83 (d, *J* = 7.5 Hz, 2H), 3.70 (q, *J* = 7.4 Hz, 2H), 3.02–2.88 (m, 6H), 2.53 (p, *J* = 8.5, 7.9 Hz, 3H), 2.46 (t, *J* = 7.5 Hz, 1H), 2.38 (t, *J* = 7.5 Hz, 2H), 2.33–2.30 (m, 2H), 1.11 (td, *J* = 7.5, 6.7, 3.8 Hz, 3H).

The ^{1}H NMR spectrum of 16-HDHEA showed that this batch, which was specifically synthesised for the NMR analysis, still contains excess ethanolamine and ethanol.

E)

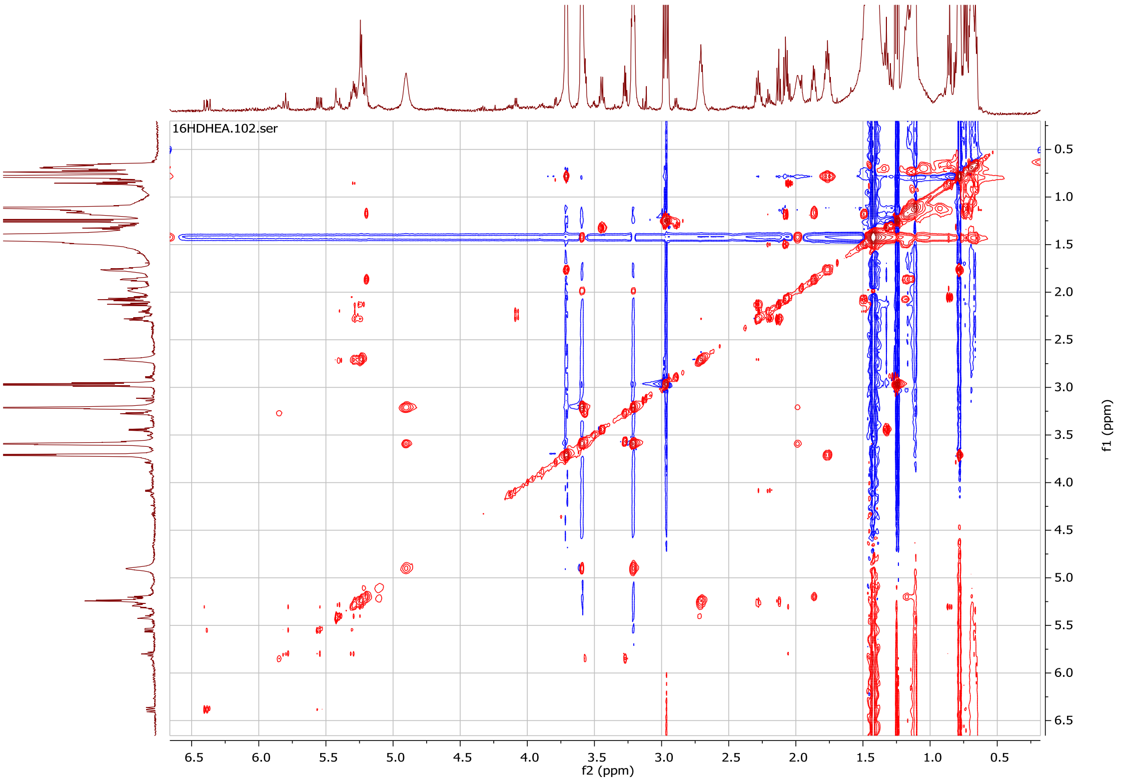

Supplemental Figure S58 – TOCSY NMR spectrum of the 16-HDHEA standard.

F)

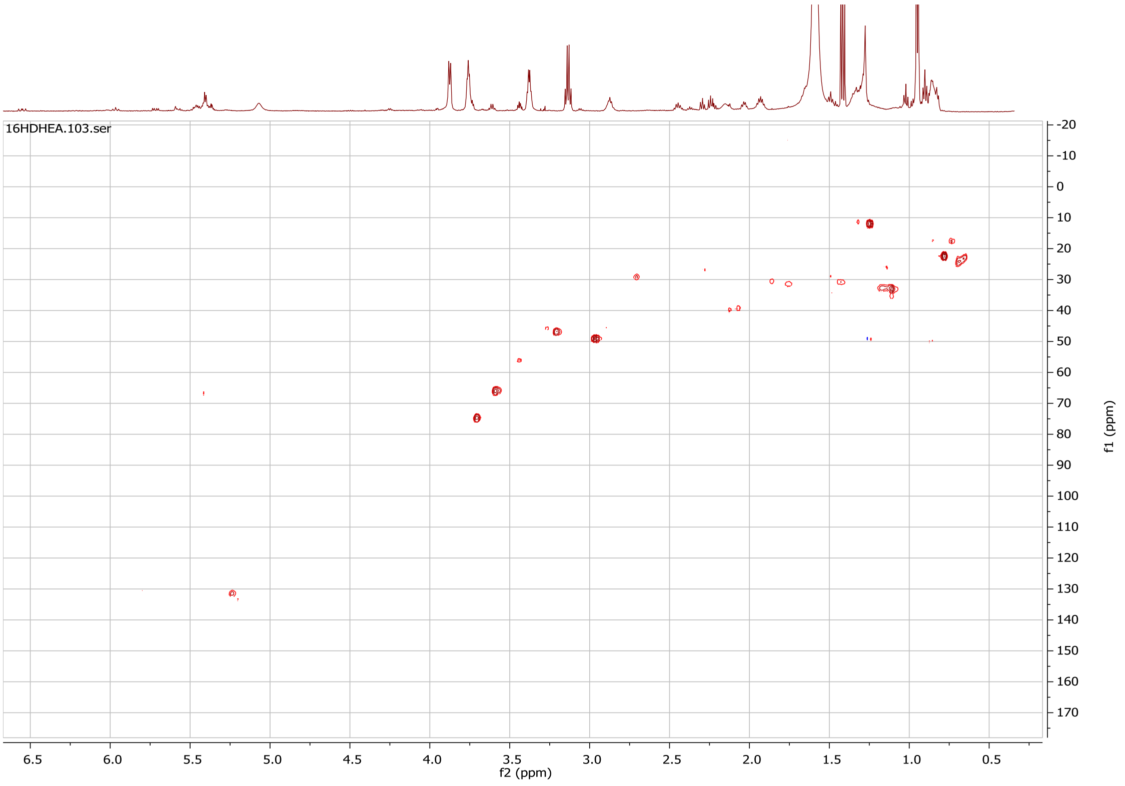

Supplemental Figure S59 – TOCSY NMR spectrum of the 16-HDHEA standard.

13-HDHEA-d_4

A)

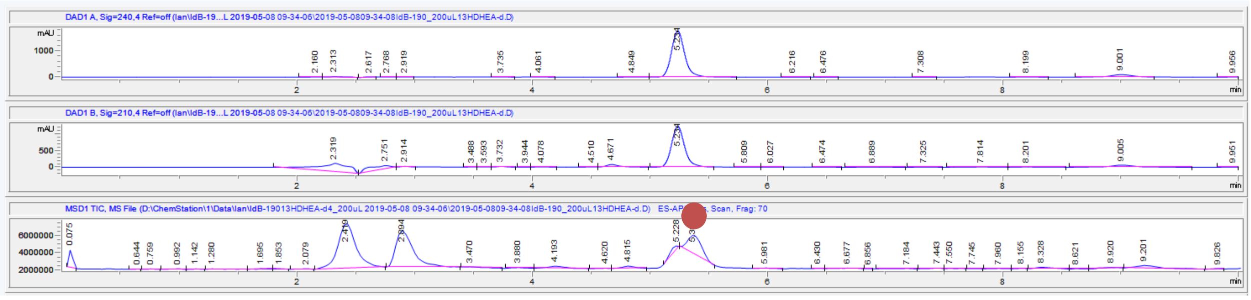

Supplemental Figure S60 – Chromatograms of the preparative HPLC purification of 13-HDHEA- d_4 (top trace: absorption at 240 nm, middle trace: absorption at 210 nm, bottom trace: TIC signal of the positive ionisation channel). The red circle represents the product peak.

B)

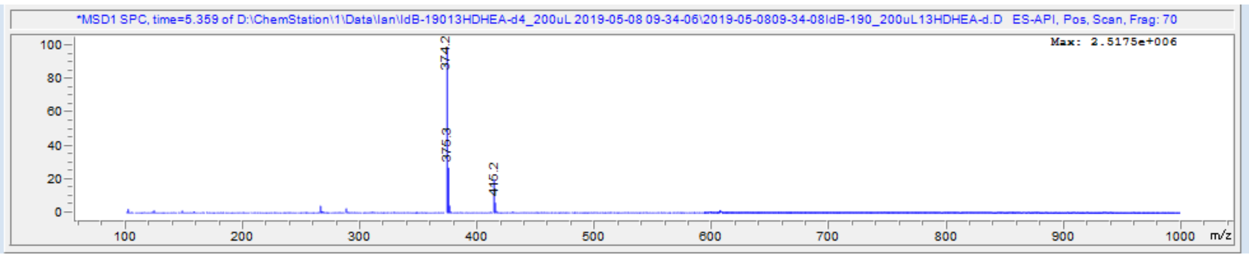

Supplemental Figure S61 – Mass spectrum of the isolated product peak of 13-HDHEA- d_4 (positive mode). The mass spectrum displayed is from the isolated product peak as indicated by the red circle in figure A.

C)

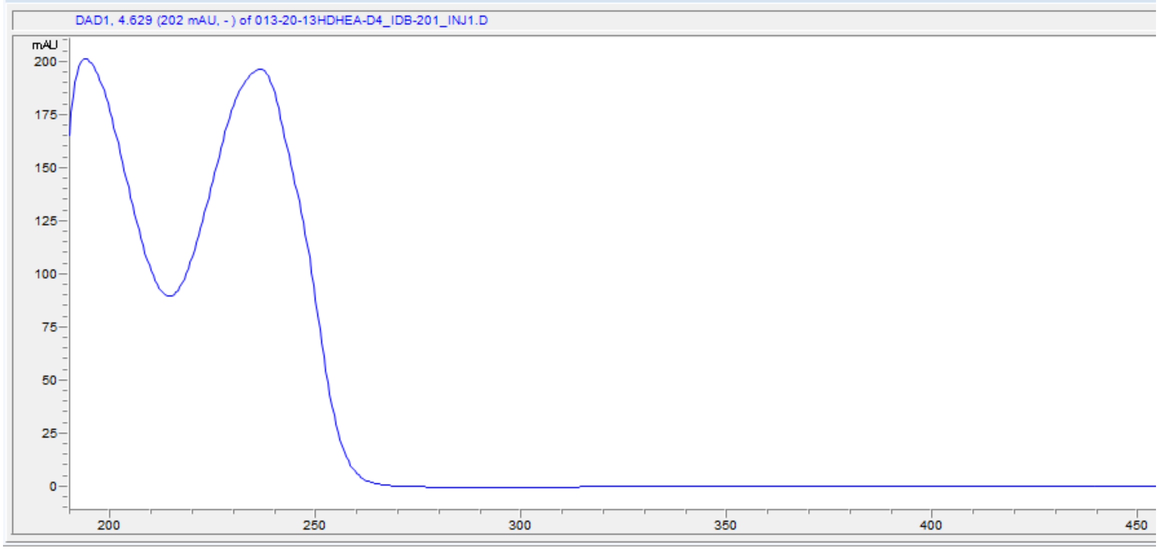

Supplemental Figure S62 – UV-absorption spectrum of the purified 13-HDHEA-d_4.

D)

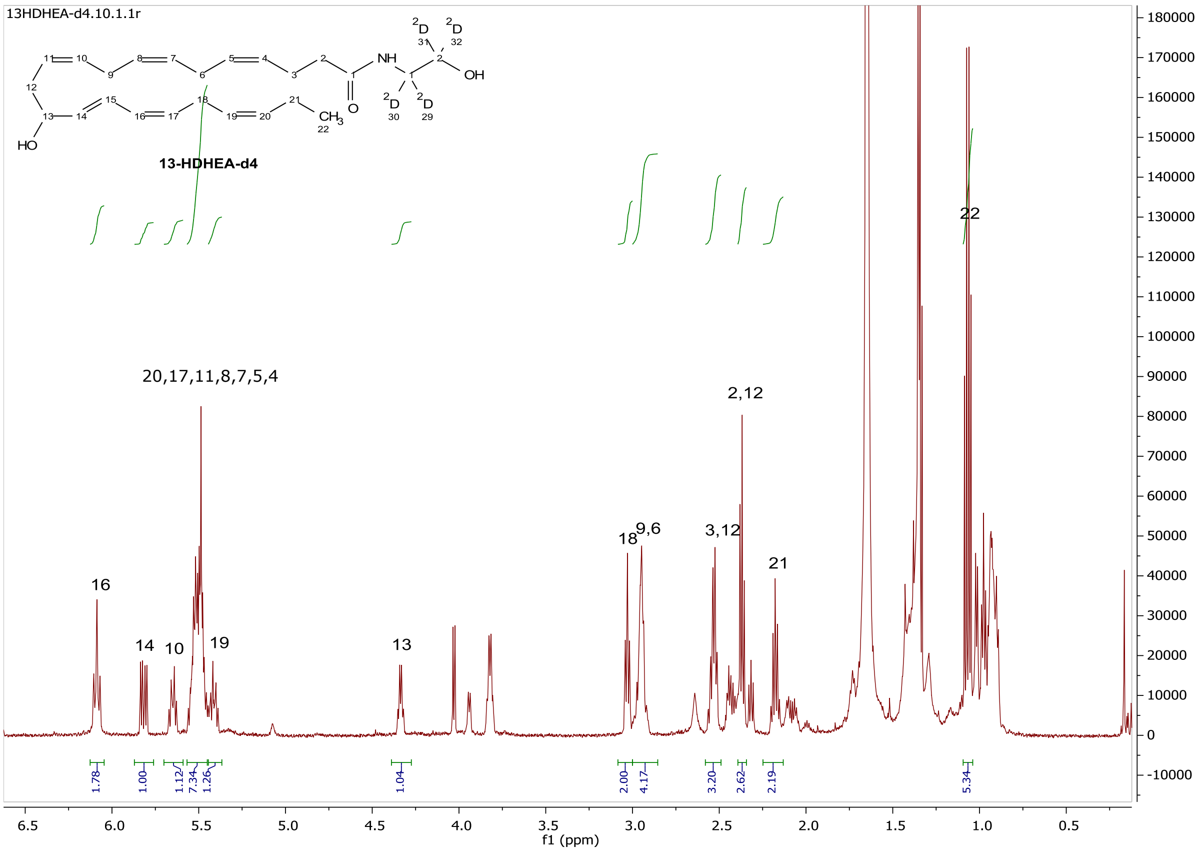

Supplemental Figure S63 – ^{1}H NMR of 13-HDHEA-*d*$_4$ standard (600 MHz, chloroform-*d*) δ 6.14–6.04 (m, 1H), 5.82 (dd, *J* = 15.2, 6.5 Hz, 1H), 5.69–5.60 (m, 1H), 5.57–5.32 (m, 8H), 4.34 (q, *J* = 6.5 Hz, 1H), 3.03 (t, *J* = 7.5 Hz, 2H), 2.96 (dt, *J* = 12.2, 6.3 Hz, 2H), 2.53 (q, *J* = 7.4, 7.0 Hz, 3H), 2.45–2.34 (m, 3H), 2.23–2.12 (m, 2H), 1.12–1.03 (m, 3H).

References belonging to the SI

1. Bezuglov, V., M. Bobrov, N. Gretskaya, A. Gonchar, G. Zinchenko, D. Melck, T. Bisogno, V. Di Marzo, D. Kuklev, J.-C. Rossi, J.-P. Vidal, and T. Durand. 2001. Synthesis and biological evaluation of novel amides of polyunsaturated fatty acids with dopamine. *Bioorg. Med. Chem. Lett.* **11**: 447–449.
2. Kotovych, G., G. H. M. Aarts, and G. Bigam. 1980. A high-field proton magnetic resonance and nuclear Overhauser effect study of prostaglandin E2. *Can. J. Chem.* **58**: 1577–1583.
3. Samuelsson, B. 1963. Prostaglandins and Related Factors. 17. The Structure of Prostaglandin E3. *J. Am. Chem. Soc.* **85**: 1878–1879.
